# siRNA conjugate with high albumin affinity and degradation resistance for delivery and treatment of arthritis in mice and guinea pigs

**DOI:** 10.1038/s41551-025-01376-x

**Published:** 2025-05-16

**Authors:** Juan M. Colazo, Megan C. Keech, Veeraj Shah, Ella N. Hoogenboezem, Justin H. Lo, Nora Francini, Nina T. Cassidy, Fang Yu, Alexander G. Sorets, Joshua T. McCune, Carlisle R. DeJulius, Hongsik Cho, Danielle L. Michell, Tristan Maerz, Kacey C. Vickers, Katherine N. Gibson-Corley, Karen A. Hasty, Leslie J. Crofford, Rebecca S. Cook, Craig L. Duvall

**Affiliations:** 1 Medical Scientist Training Program, Vanderbilt University School of Medicine, Nashville, TN, USA.; 2 Department of Biomedical Engineering, Vanderbilt University, Nashville, TN, USA.; 3 Department of Orthopaedic Surgery, Washington University in St Louis, St Louis, MO, USA.; 4 Department of Medicine, Vanderbilt University Medical Center, Nashville, TN, USA.; 5 Vanderbilt-Ingram Cancer Center, Vanderbilt University Medical Center, Nashville, TN, USA.; 6 Department of Orthopaedic Surgery and Biomedical Engineering, UTHSC, Memphis VA Medical Center, Memphis, TN, USA.; 7 Department of Orthopaedic Surgery, University of Michigan, Ann Arbor, MI, USA.; 8 Department of Pathology, Microbiology, and Immunology, Division of Comparative Medicine, Vanderbilt University Medical Center, Nashville, TN, USA.; 9 Department of Medicine, Division of Rheumatology, Vanderbilt University Medical Center, Nashville, TN, USA.

## Abstract

Osteoarthritis and rheumatoid arthritis are debilitating joint diseases marked by pain, inflammation and cartilage destruction. Current osteoarthritis treatments only relieve symptoms, while rheumatoid arthritis therapies can cause immune suppression and provide variable efficacy. Here we developed an optimized small interfering RNA targeting matrix metalloproteinase 13 for preferential delivery to arthritic joints. Chemical modifications in a stabilizing ‘zipper’ pattern improved RNA resistance to degradation, and two independent linkers with 18 ethylene glycol repeats connecting to tandem C18 lipids enhanced albumin binding and targeted delivery to inflamed joints following intravenous administration. In preclinical models of post-traumatic osteoarthritis and rheumatoid arthritis, a single intravenous injection of the albumin-binding small interfering RNA achieved long-term joint retention, sustained gene silencing and reduced matrix metalloproteinase 13 activity over 30 days, resulting in decreased cartilage erosion and improved clinical outcomes, including reduced joint swelling and pressure sensitivity.

This approach demonstrated superior efficacy over corticosteroids and small-molecule MMP inhibitors, highlighting the therapeutic promise of albumin ‘hitchhiking’ for targeted, systemic delivery of gene-silencing therapeutics to treat osteoarthritis and rheumatoid arthritis.

Osteoarthritis (OA) is a degenerative joint disease that can be idiopathic or secondary to injury, in the case of post-traumatic osteoarthritis (PTOA). OA is characterized by cartilage loss, synovial inflammation and formation of osteophytes, all of which contribute to pain and lost joint function^1^. Patients often develop broader multijoint osteoarthritis (MJOA), which can have debilitating impact on quality of life^2^. The autoimmune disease rheumatoid arthritis (RA) has independent aetiology and is characterized by severe synovial and systemic inflammation, bone erosion and cartilage damage^3,4^. Current OA treatment options are palliative and include non-steroidal anti-inflammatory drugs (NSAIDS) or corticosteroids, approaches that neither counteract the root causes of disease nor prevent OA progression; corticosteroids may in fact worsen cartilage thinning^5-7^. Current treatments for RA include synthetic disease-modifying antirheumatic drugs (DMARDs) and biologic DMARDs such as tumour necrosis factor (TNF) inhibitors, interleukin (IL) inhibitors and B-cell inhibitors. These treatments are efficacious, although they can suppress the immune system, and their benefits often wane over the long term^8^.

There is extensive overlap between OA and RA in terms of the downstream molecular effectors that drive joint degeneration^9^. Both are associated with inflammation and upregulation of extracellular matrix (ECM)-degrading matrix metalloproteinases (MMPs)^10^. MMPs erode cartilage and the degradation byproducts have inflammatory signalling properties that further induce expression of inflammatory cytokines and MMPs, perpetuating a chronic catabolic cycle that leads to deep cartilage erosion, chondrocyte loss and subchondral bone exposure^11-13^.

Therapeutic MMP inhibition is a logical approach for OA and RA treatment. Development of MMP-selective antagonists is critical, as broad spectrum MMP inhibitors cause musculoskeletal toxicities in humans, probably due to universal disruption of normal tissue homeostasis^14-16^. Among MMPs, MMP13 is perhaps the most efficient at cleaving the key ECM components collagen II^17^ and fibronectin^18^, producing inflammatory fragments. Further, knockout mouse studies pinpoint MMP13 as a key proteolytic driver of cartilage loss in arthritis models^19^. Lastly, MMP13 expression is relatively confined to joint tissues and diseased or injured tissues characterized by fibrosis or inflammation^20^. This narrow expression pattern allows for a potentially wider therapeutic window with minimal toxicities. However, this hypothesis has yet to be tested fully, as clinical development of selective MMP inhibitors has been challenged by the extensive structural similarities between the catalytic sites of the various MMPs^21^.

RNA interference (RNAi) provides an ideal technique for gene-selective inhibition, as specificity is conferred by the genetic sequence. The translatability of small interfering (si)RNA therapeutics is also now well established based on the FDA approval of lipid nanoparticle and GalNAc–siRNA conjugate drugs, both of which have been successfully applied in humans for gene silencing in the liver^22,23^. Selective MMP13 gene knockdown using siRNA sequences would overcome the hurdle of structural similarity between MMPs, thus providing an opportunity to target MMP13 in the context of OA and RA. This notion is supported by recent PTOA model studies in which intra-articular delivery of MMP13 silencing siRNA nanoparticles (siNPs) protected against knee joint load-induced cartilage loss^24,25^.

To improve on these relatively complex nano- and microtechnologies for intra-articular MMP13 siRNA delivery, we sought an approach in which carrier-free siRNA conjugates delivered intravenously might preferentially accumulate within arthritic joints. A systemically delivered approach avoids the potential joint damage that can occur from repeated intra-articular injections and better enables treatment of MJOA and RA. Relative to siNPs, a molecularly defined conjugate is also simpler to manufacture and does not come with the added risk of carrier toxicities that often narrow the therapeutic window of cationic lipid or polymer siNP formulations.

Development of carrier-free siRNA conjugates for rheumatic diseases is an unchartered but high-impact endeavour^26^. Toward this goal, experimental siRNA–lipid conjugates that commandeer circulating plasma albumin for delivery to arthritic joints were engineered and tested in models of OA and RA. Albumin has unique promise as a therapeutic siRNA carrier, given its long circulation half-life (20 days)^27^ and natural tendency to accumulate in inflamed tissues, including arthritic joints^28-31^. Albumin has natural binding sites for long-chain fatty acids^27,32-36^ that can be co-opted by drugs or fatty acid-modified exogenous molecules, as exemplified by clinically approved albumin-based/binding formulations (for example, Abraxane, Semaglutide, Levemir, Optison and Tirzepatide)^37-39^. Binding to these sites yields the potential for increasing drug pharmacokinetic properties, as albumin accesses natural recycling and kidney reabsorption mechanisms that prevent its degradation and urinary clearance, thus prolonging its half-life^27,40-43^. Hitchhiking on albumin also can promote preferential accumulation in arthritic joints because their inflamed state causes leaky vasculature and permits local drug extravasation and sequestration (the ‘ELVIS’ effect^44,45^). Albumin is one of the most prominent proteins in synovial fluid (SF)^46,47^, and it also shows cartilage penetration, being 15-fold more concentrated in the deep layers of arthritic than healthy cartilage and having 2-fold higher penetration into human cartilage than IgG antibodies^28,29^. Recombinant fusion to albumin promotes protein accumulation in inflamed knees^31,48^, further motivating our approach.

Although covalent conjugation of siRNA to albumin has been explored to some extent^49,50^, lipid–siRNA conjugates that enable reversible in situ association with native circulating albumin has been proof-of-concept tested mostly in cancer studies^32,51,52^. We show here that systemic delivery of an albumin-hitchhiking siRNA–lipid conjugate targeting MMP13 achieves robust delivery, MMP13 silencing and therapeutic efficacy in joints afflicted by OA and RA.

## Results and discussion

### Circulating albumin robustly accumulates in PTOA joints

**Fig. 1 ∣:**
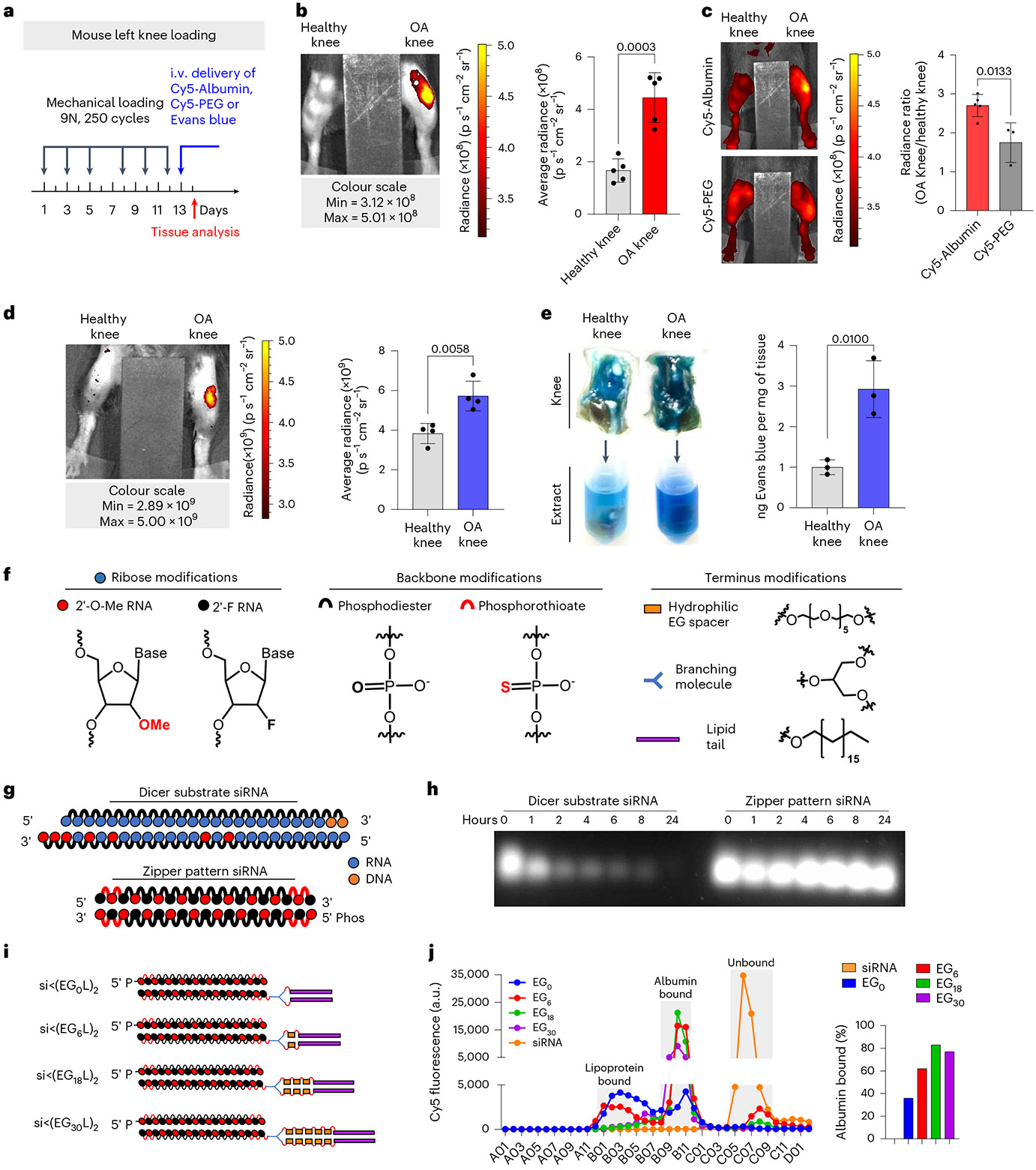
Lipid conjugated siRNA constructs for albumin-mediated delivery to PTOA joints. **a**–**d**, Left-knee mechanical loading was used to induce unilateral PTOA in mice, followed by i.v. delivery of 3 mg kg^−1^ MSA-Cy5 (*N* = 5), 3 mg kg^−1^ PEG-Cy5 (*N* = 3) and 200 μl of 2% w/v Evans blue (*N* = 4). Cy5 and Evans blue were measured by fluorescence imaging 24 h after delivery. Representative images are shown. **a**, Experimental timeline. **b**, MSA-Cy5 fluorescence in paired PTOA and healthy knees. **c**, Ratio of MSA-Cy5 and PEG-Cy5 fluorescence in PTOA and healthy knees for each treated mouse. **d**, Intravital Evans blue fluorescence in paired contralateral PTOA and contralateral non-loaded knees. **e**, PTOA and contralateral non-loaded knees were excised 24 h after Evans blue delivery. Evans blue was extracted and measured by densitometry (*N* = 3). Representative images of joints and resulting joint extracts are shown. **f**, Chemical modifications of siRNA ribose, backbone and terminus. **g**, Schematic of synthetic dicer-substrate siRNA and alternating 2′F and 2′OMe modified ‘zipper’ siRNA. **h**, siRNA stability in OA-derived synovial fluid: representative gel electrophoresis of dicer substrate and zipper siRNA sequences after incubation in synovial fluid collected from an untreated OA patient. **i**, Schematic representation of the si<(EG_X_L)_2_ series of zipper-modified siRNAs with divalent lipid end-modifications with variations in EG content. **j**, Left: FPLC chromatograph of elution of si<(EG_X_L)_2_ variants pre-incubated with human synovial fluid form normal joints. Right: percentage of total si<(EG_X_L)_2_ bound to albumin fractions. All error bars indicate s.d. of biological replicates. Knees labelled healthy indicate a non-loaded contralateral limb (right).

To determine the extent to which circulating albumin accumulates in arthritic joints, we intravenously (i.v.) delivered Cy5-labelled mouse serum albumin (MSA-Cy5) into mice with induced PTOA. In this model, the left knee undergoes repeated loading to create damage and inflammation, while the right knee remains unchallenged (Fig. 1a). At 24 h after delivery, abundant MSA-Cy5 was observed in the synovium and cartilage of PTOA knees but not in contralateral control knees (Fig. 1b and Supplementary Fig. 1). In contrast, Cy5-conjugated poly(ethylene glycol) (PEG-Cy5) did not accumulate preferentially in PTOA knees (Fig. 1c). Further, i.v. delivery of the albumin binding dye Evans blue resulted in preferential accumulation in synovia and cartilage of PTOA knees, but not contralateral healthy knees (Fig. 1d,e, and Supplementary Figs. 2 and 3). Interestingly, mRNAs of genes encoding the albumin delivery/transport factors caveolin-1 and secreted protein acidic and rich in cysteine (SPARC) were elevated in PTOA joint tissues (Supplementary Fig. 3d), consistent with previous reports of SPARC upregulation in arthritic joints^53-56^. Further, interstitial SPARC expression in tumours correlates with efficacy of albumin-based therapeutics in head and neck cancers^57^. These findings support the idea that hitchhiking on endogenous circulating albumin might be used to skew the biodistribution of siRNA conjugates to arthritic joints following i.v. delivery.

### Enhanced stability, albumin binding and PTOA joint accumulation of optimized siRNA–lipid conjugates

Carrier-free siRNA conjugates must be chemically modified to protect against degradation by endo- and exonucleases. To stabilize the siRNAs, we engineered blunt-ended 19-mer RNA strands with alternating 2′-O-Me and 2′-F ribose modifications in a ‘zipper’ pattern and replaced phosphodiesters with phosphorothioate (PS) linkages for the last two bases of the 5′- and 3′-termini of the sense and antisense strands (Fig. 1f,g). Stability of the zipper-modified siRNA was assessed in synovial fluid from a treatment-naive OA patient, revealing siRNA stability through at least 24 h at 37 °C, whereas a more lightly modified dicer-substrate siRNA was >50% degraded within 1 h (Fig. 1h). Given the stability of the zipper-modified siRNA, all further siRNA modifications were done using this modification pattern.

The zipper siRNAs were end modified with bivalent C18 lipids intended to promote binding of the siRNA into the natural fatty acid (FA) binding pockets of albumin (Fig. 1i)^32^. A library of bivalent lipid structures was synthesized as recently reported^51^. Briefly, a splitter phosphoramidite (<) was added at the 5′ end of the sense strand, followed by addition of 0 (EG_0_), 1 (EG_6_), 3 (EG_18_) or 5 (EG_30_) hexa-ethylene glycol (EG_6_) phosphoramidite spacers to each branch. Both branches were terminally appended with a C_18_ lipid, thus generating siRNA<(EG_x_L)_2_, where X is the number of EG repeats linking the splitter to each terminal C18.

Binding of the different siRNA structures to albumin within human synovial fluid was assessed using fast protein liquid chromatography (FPLC). A proportion of each of the siRNA<(EG_X_L)_2_ conjugates was found in the albumin-containing synovial fluid fractions (Fig. 1j), whereas unconjugated, zipper-pattern modified siRNA was not. Notably, >80% of the total siRNA<(EG_18_L)_2_ detected by FPLC was found in the albumin-containing fraction, higher than any other siRNA–lipid conjugate tested. This observation was consistent with the previously measured high affinity binding of siRNA<(EG_18_L)_2_ to albumin (*K*_D_ = 30 nM)^51^.

**Fig. 2 ∣:**
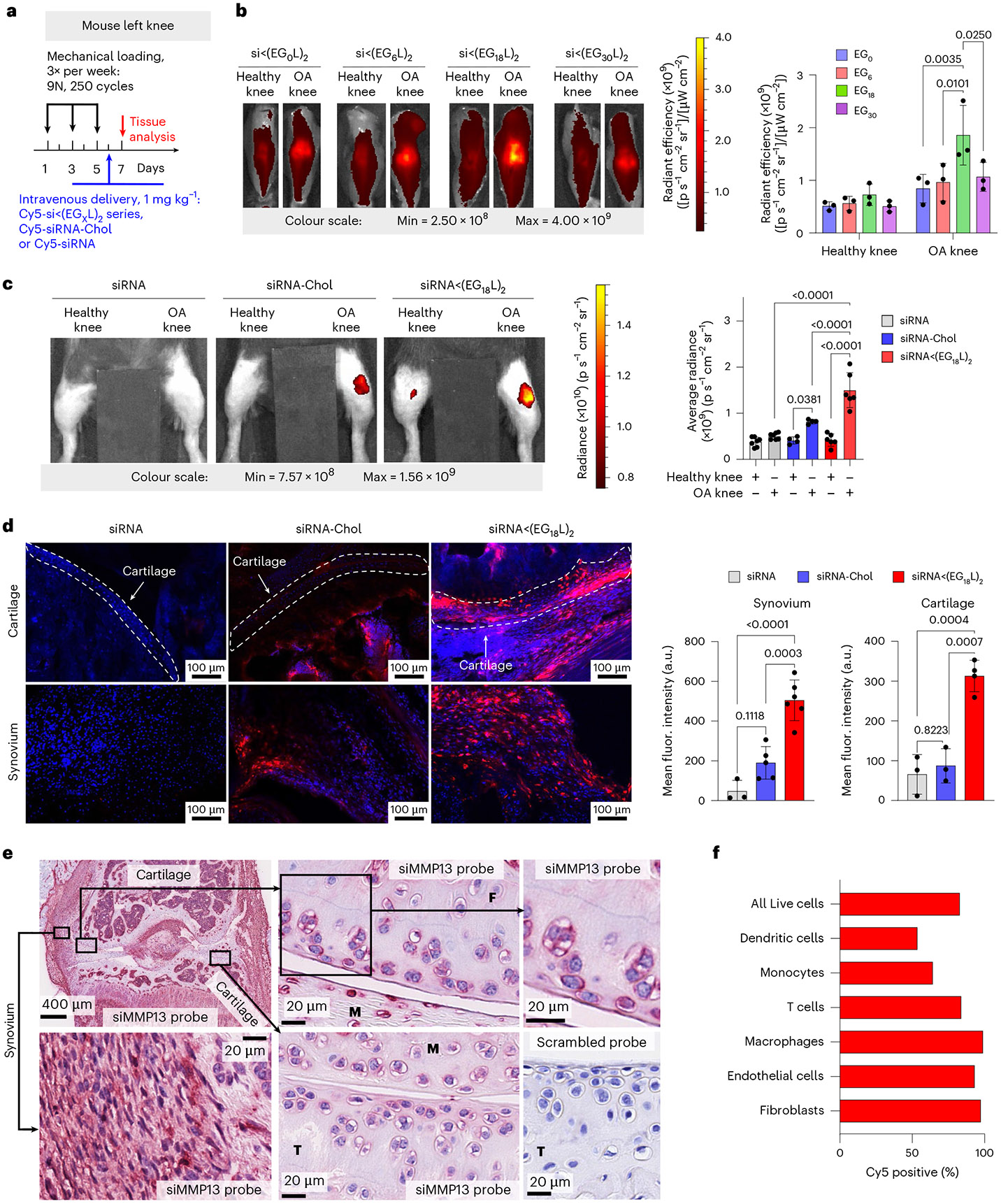
Accumulation of the albumin-binding siRNA–lipid conjugate, siRNA<(EG_18_L)_2_, in mouse PTOA knee joints following i.v. delivery. **a**, Unilateral left knee mechanical loading, treatment (1 mg kg^−1^ i.v.) and endpoint protocol used in **b**–**d**. **b**, Representative ex-vivo IVIS images and quantitation of Cy5-labelled siRNA<(EGxL)_2_ accumulation in mouse knees 24 h after delivery. **c**, Left: representative intravital Cy5 fluorescence images taken 24 h after delivery of Cy5-labelled siRNA, siRNA-Chol or siRNA<(EG_18_L)_2_. Right: quantification of average Cy5 fluorescence in knees taken 24 h after mouse i.v. injection. Analysed using mixed effects analysis. **d**, DAPI-counterstained cryosections of knees from treated mice imaged and quantified for Cy5-labelled siRNA fluorescence in cartilage and synovial tissue. Dashed lines outline articular cartilage. **e**, In situ hybridization with an siMMP13 probe showing signal localization of siMMP13<(EG_18_L)_2_ in mice treated i.v. with 10 mg kg^−1^. Representative images show siRNA presence in the femoral cartilage (F), meniscus (M), tibial cartilage (T) and synovium. Serial tissue sections incubated with a negative control scrambled probe showed no signal. **f**, Flow cytometry assessment of cell type-specific uptake of Cy5-siRNA<(EG_18_L)_2_ in synovium. Mice were treated i.v. with 10 mg kg^−1^ following three bilateral mechanical loading sessions over a week. Cells isolated from synovia of 5 mice were pooled for analysis. Experimental and flow cytometry gating details are in Supplementary Fig. 11. All error bars indicate s.d. of biological replicates. Knees labelled healthy indicate a non-loaded contralateral limb (right).

Cy5-labelled siRNA<(EG_X_L)_2_ constructs were next delivered i.v. (1 mg kg^−1^) to mice with PTOA induced in the left knee (Fig. 2a). While each accumulated to a greater extent in PTOA knees over healthy contralateral knees, siRNA<(EG_18_L)_2_ achieved the highest accumulation in PTOA joints (Fig. 2b and Supplementary Fig. 4). A benchmarking study was done to compare siRNA<(EG_18_L)_2_ to commonly used cholesterol-conjugated siRNA (siRNA-Chol). In contrast to the >4-fold enrichment of siRNA<(EG_18_L)_2_ in PTOA knees over healthy knees, parental siRNA and cholesterol-conjugated siRNA-Chol each showed only a 1.5- and 2-fold enrichment of delivery to PTOA vs healthy knees, respectively (Fig. 2c). Histological cryosections of PTOA knee synovium and cartilage illustrated holistic joint tissue penetration and cellular uptake of Cy5-labelled siRNA<(EG_18_L)_2_ (Supplementary Figs. 5-7), while siRNA-Chol was observed but at lower magnitude within PTOA joint compartments (Fig. 2d). Organ biodistribution studies performed by ex vivo Cy5 imaging showed that liver was the main off-target delivery site for both siRNA<(EG_18_L)_2_ and siRNA-Chol, while unconjugated siRNA accumulated primarily in kidneys (Supplementary Fig. 8), a major site of siRNA clearance.

Interactions between albumin and siRNA<(EG_18_L)_2_ were further assessed by FPLC of synovial fluid from OA, RA or normal joints. Similar to what was observed using healthy patient-derived synovial fluid, Cy5-siRNA<(EG_18_L)_2_ eluted primarily in the albumin-containing fractions of OA patient and RA patient-derived synovial fluid samples, but with substantially greater intensity in arthritic samples (Supplementary Fig. 9a). In contrast, the parent Cy5-siRNA remained predominantly unbound. Western blotting confirmed the increased albumin content in OA- and RA-derived synovial fractions B12 and C1, the same fractions that siRNA<(EG_18_L)_2_ was predominantly associated with (Supplementary Fig. 9b). Complementary native gel electrophoresis analyses of synovial fluid fractions B12-C1 revealed co-localization of Cy5-siRNA<(EG_18_L)_2_ with a Coomassie-stained protein of 66 kDa, the known molecular weight of albumin (Supplementary Fig. 9c). Further, siRNA<(EG_18_L)_2_ showed stability in synovial fluid from OA joints for at least 96 h (Supplementary Fig. 9d). Taken together, these findings allowed us to assign siRNA<(EG_18_L)_2_ as our lead siRNA–lipid conjugate for albumin hitchhiking to arthritic joints, motivating a focus on this construct for subsequent therapeutic studies. We also found that siRNA<(EG_18_L)_2_ interacted with albumin across multiple species, including human, mouse and guinea pig, supporting its utility for all the OA and RA models used herein (Supplementary Fig. 9e).

### PTOA joint accumulation and cell type-specific uptake of siRNA<(EG_18_L)_2_

RNA in situ hybridization (ISH) was utilized for label-free visualization of tissue localization and penetration of the siMMP13<(EG_18_L)_2_ delivered i.v. at a dose of 10 mg kg^−1^. Application of an ISH probe designed against siMMP13 to PTOA joint histological sections revealed the presence of siMMP13<(EG_18_L)_2_ within the meniscus and femoral/tibial cartilage, in addition to synovial localization (Fig. 2e). This label-free method illustrates that the therapeutically active siMMP13<(EG_18_L)_2_ compound achieves delivery and penetration into important joint tissues with known relevance to PTOA progression. To validate the reproducibility and specificity of the ISH approach, we also observed positive staining with a probe designed against the endogenous small nuclear RNA RNU6; furthermore, application of a negative control probe to the histological sections taken from PTOA joints of mice treated with siMMP13<(EG_18_L)_2_ or a saline vehicle control showed no visible staining, as did application of the siMMP13 probe to PTOA joint sections from saline-treated mice (Fig. 2e and Supplementary Fig. 10).

To characterize cell type-specific uptake of the selected siRNA<(EG_18_L)_2_ conjugate, Cy5-siRNA<(EG_18_L)_2_ (10 mg kg^−1^) or vehicle was delivered i.v. to mice with mechanical load-induced PTOA in both knees. Cell type-specific uptake by synovial fibroblasts, endothelial cells, macrophages, monocytes, dendritic cells and T cells was characterized 24 h after treatment using flow cytometry (Fig. 2f and Supplementary Fig. 11). A high percentage (83.1%) of the overall cells in the synovium were positive for uptake of Cy5-labelled siRNA<(EG_18_L)_2_, with fibroblasts, macrophages and endothelial cells exhibiting the highest proportion of Cy5-positive cells (>90%).

### siRNA<(EG_18_L)_2_ accumulation in PTOA joints following subcutaneous and intra-articular delivery

**Extended Data Fig. 1 ∣:**
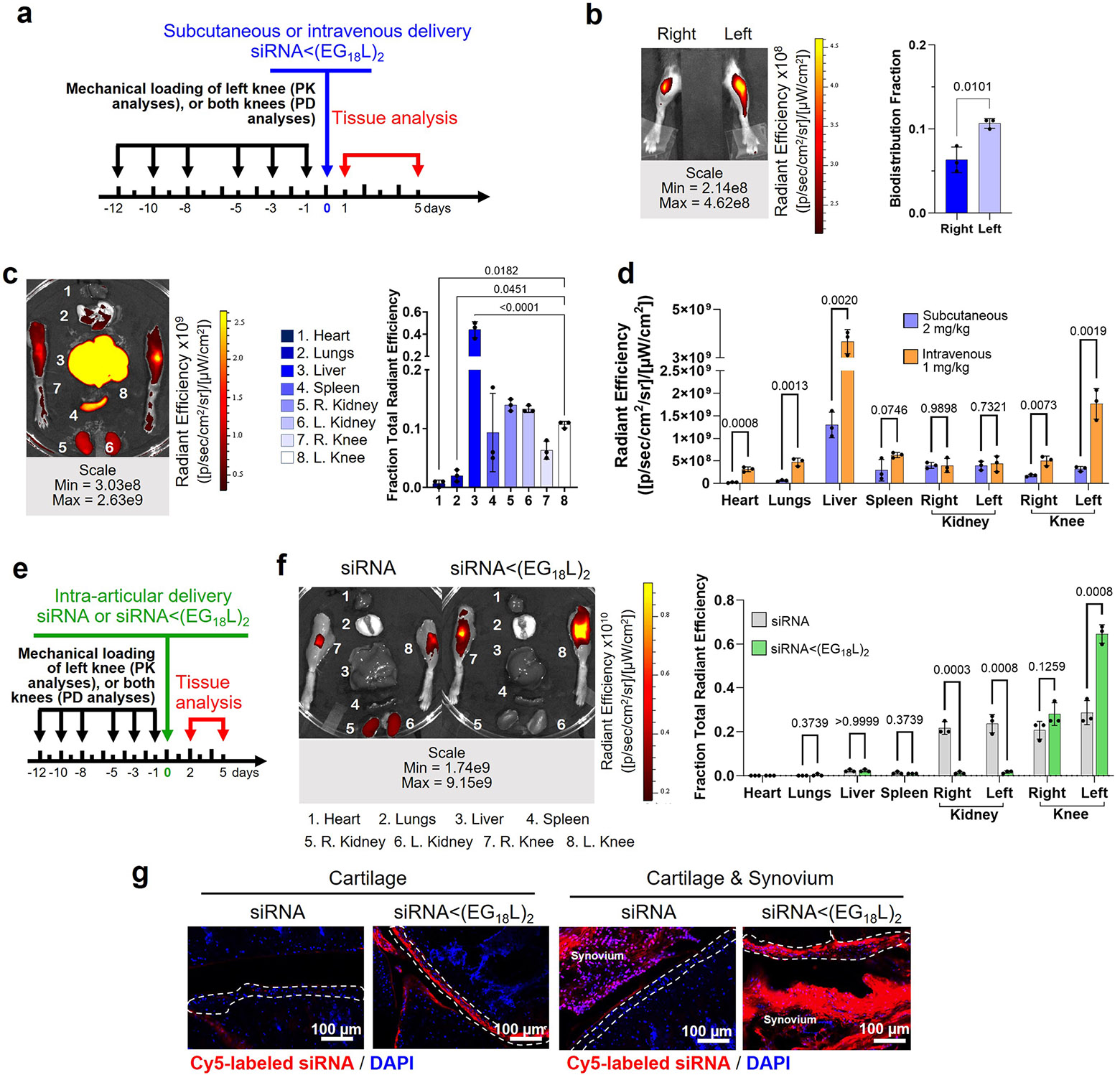
Characterizing different routes of siRNA<(EG_18_L)_2_ delivery in PTOA mouse model. **a-c**, Subcutaneous delivery of Cy5-siRNA<(EG_18_L)_2_ (2 mg/kg) was administered in a PTOA mouse model. Timeline for mechanical knee loading and treatment is shown (**a**). Intravital (**b**) and ex vivo (**c**) Cy5 fluorescence imaging was used to measure Cy5-siRNA<(EG_18_L)_2_ biodistribution to paired healthy and PTOA knees, along with organs, 24 hrs after subcutaneous treatment. N = 3. **d**, Quantification of subcutaneous vs. intravenous (i.v.) delivery route organ biodistribution 24 h after injection. Analyzed using multiple unpaired t-tests with no correction for multiple comparisons. **e-g**, Intra-articular delivery of Cy5-siRNA<(EG_18_L)_2_ (0.25 mg/kg) was characterized in a PTOA mouse model. Timeline for mechanical knee loading and treatment is shown (**e**). Ex vivo Cy5 fluorescence imaging was used to measure Cy5-siRNA<(EG_18_L)_2_ biodistribution to paired healthy and loaded knees, along with organs, 48 hrs after delivery (**f**). N = 3. Analyzed using multiple unpaired t-tests with no correction for multiple comparisons. (**g**) Cryohistology of the loaded knee joints 48 h after intra-articular injection with a focus on cartilage and synovial tissues. N = 3. Dashed lines outline articular cartilage. For all panels: Error bars indicate standard deviation of biological replicates. Radiant efficiency units are [photons/s] / [μW/cm^2^]. Knees labeled healthy indicate a non-loaded contralateral limb (right).

Subcutaneous injection of siRNA<(EG_18_L)_2_ was tested as an alternative to the i.v. delivery route, given the utility of subcutaneous injections for patient self-administration of biologic drugs. Subcutaneous delivery of Cy5-siRNA<(EG_18_L)_2_ (2 mg kg^−1^) enabled preferential siRNA accumulation in PTOA knees over contralateral non-injured knees, with similar organ biodistribution profile as i.v. delivery (Extended Data Fig. 1a-c). However, subcutaneous delivery of siRNA<(EG_18_L)_2_ at 2 mg kg^−1^ did not achieve the absolute level of siRNA accumulation in PTOA knees seen with i.v. delivery at 1 mg kg^−1^ (Extended Data Fig. 1d). Notably, a large amount of the Cy5-siRNA<(EG_18_L)_2_ dose was retained at the injection site (data not shown), perhaps due to its rapid interaction with subcutaneous fat or cells at the injection site^58^. Intra-articular (i.a.) delivery, a common clinical route for steroid delivery, was also tested (0.25 mg kg^−1^), revealing greater Cy5-siRNA<(EG_18_L)_2_ retention in the PTOA over healthy knee and greater retention than Cy5-siRNA at 48 h post injection, achieving penetration into cartilage and synovia (Extended Data Fig. 1e-g and Supplementary Fig. 7b). While i.a.-delivered Cy5-siRNA<(EG_18_L)_2_ was retained primarily in the knee, Cy5-siRNA redistributed to kidneys, reflecting its relatively rapid removal from the joint by synovial drainage via vasculature and lymphatics, underscoring the importance of the lipid moieties for retention of siRNA<(EG_18_L)_2_ within the joint space.

### Potent MMP13 knockdown in PTOA-afflicted joints by siRNA<(EG_18_L)_2_

siRNA sequences against mouse and guinea pig MMP13 (siMMP13) were screened in mouse ATDC5 chondrogenic cells and primary guinea pig chondrocytes, respectively, for MMP13 knockdown potency (Supplementary Fig. 12). Lead candidate sequences were then synthesized with the zipper modification pattern and MMP13 silencing potency reconfirmed upon transfection into TNF-stimulated ATDC5 cells (Supplementary Fig. 13). Albumin-binding zipper-pattern siMMP13 was then synthesized, thus generating siMMP13<(EG_18_L)_2_. Importantly, siMMP13<(EG_18_L)_2_, but not the parent zipper-pattern modified siMMP13, provided robust carrier-free (no lipofection reagent) *Mmp13* knockdown (Supplementary Fig. 13). The siRNA<(EG_18_L)_2_ structure also showed efficient cellular uptake in ATDC5 cells; level of uptake was partially diminished in the presence of excess albumin but increased upon treatment with pro-inflammatory TNF stimulation, even in the presence of excess albumin (Supplementary Fig. 14).

**Extended Data Fig. 2 ∣:**
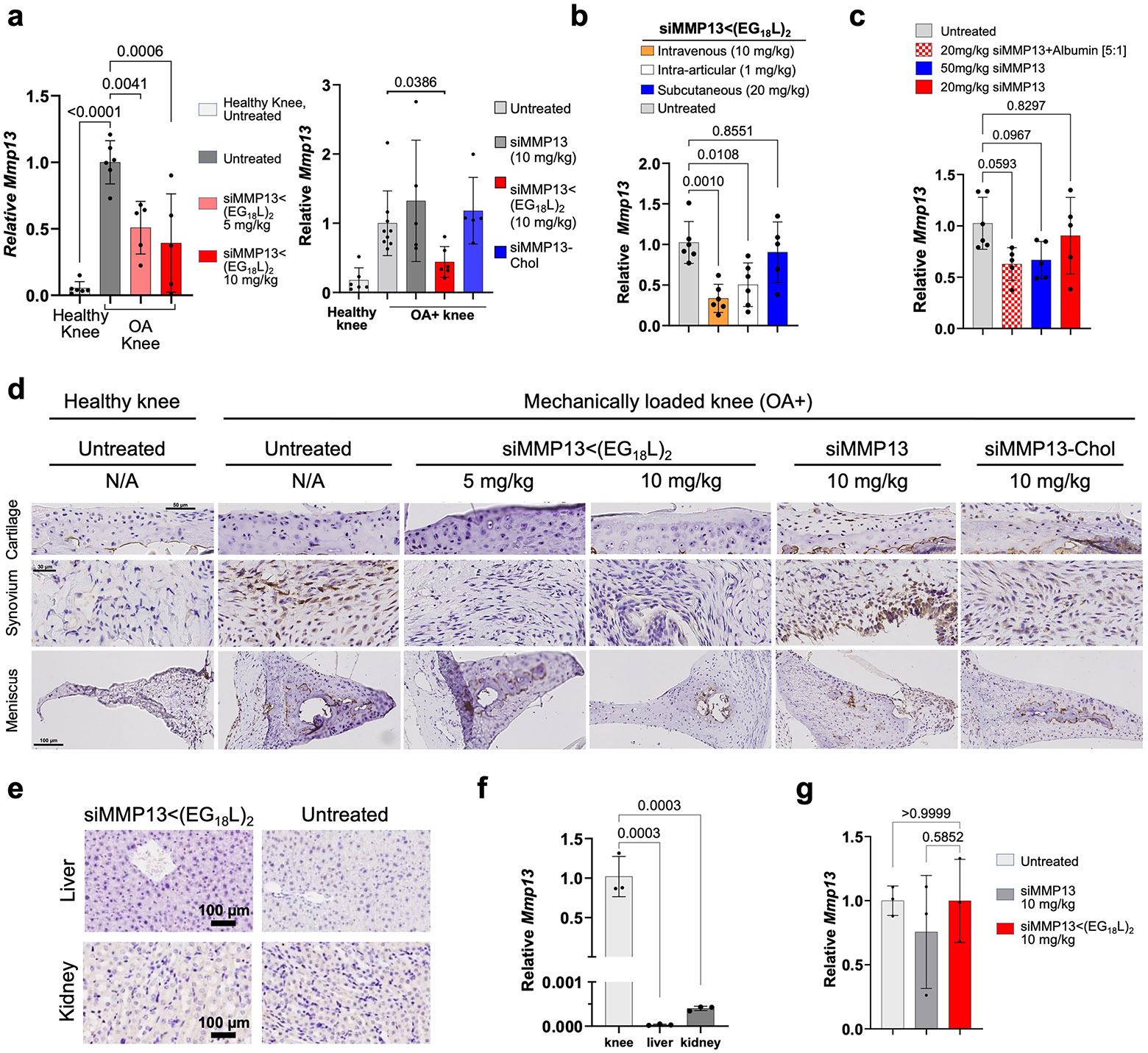
MMP13 knockdown in PTOA joints by i.v. delivery of siMMP13<(EG_18_L)_2_. **a**, *Mmp13* mRNA was measured by qRT-PCR in healthy and PTOA knees of mice 5 days after i.v. delivery of siMMP13<(EG_18_L)_2_, siMMP13-Chol, or free siMMP13 (5 and 10 mg/kg, where indicated). Timeline for mechanical knee loading and treatment is shown in Extended Data Fig 1. **b**, *Mmp13* mRNA was measured by qRT-PCR in PTOA mouse knees 5 days after i.v., i.a., or subcutaneous siMMP13<(EG_18_L)_2_ delivery at the doses indicated. N = 5-6. **c**, *Mmp13* mRNA was measured by qRT-PCR in PTOA mouse knees 5 days after subcutaneous siMMP13<(EG_18_L)_2_ delivery at the doses indicated. N = 5-6. **d-e**, MMP13 IHC in PTOA knees (**d**), liver (**e**, top), and kidneys (**e**, bottom) 5 days after i.v. delivery (10 mg/kg) of siMMP13<(EG_18_L)_2_, siMMP13-Chol, free siMMP13, or no treatment. **f**, Relative *Mmp13* mRNA expression in liver and kidneys of untreated mice (relative to PTOA knee *Mmp13* levels). N = 3. **g**, Relative *Mmp13* mRNA in kidneys, 5 days post-injection with 10 mg/kg siMMP13<(EG_18_L)_2_ or free siMMP13, vs. untreated controls. N = 3. For all panels: Error bars indicate standard deviation of biological replicates.

MMP13 silencing by siMMP13<(EG_18_L)_2_ in PTOA-affected mouse knees was first assessed 5 days after i.v. treatment, finding >50% *Mmp13* knockdown with a 5 mg kg^−1^ dose and >60% with a 10 mg kg^−1^ dose (Extended Data Fig. 2a). Neither unconjugated siMMP13 nor siMMP13-Chol (each at 10 mg kg^−1^ i.v.) significantly affected *Mmp13* levels in PTOA knees. A single i.a. injection of siMMP13<(EG_18_L)_2_ at 1 mg kg^−1^ also decreased *Mmp13* in PTOA knees (Extended Data Fig. 2b). While subcutaneous delivery of siMMP13<(EG_18_L)_2_ at a relatively high dose (50 mg kg^−1^) diminished *Mmp13* in PTOA knees (Extended Data Fig. 2c), a lower dose (20 mg kg^−1^) reduced *Mmp13* in PTOA knees only when co-delivered with an excess of mouse albumin (1:5 molar ratio). These results show promise for use of siMMP13<(EG_18_L)_2_ with multiple delivery routes. However, subcutaneous delivery may benefit from more optimization of excipients or penetration enhancers to improve absorption from the injection site into the systemic circulation.

Immunohistochemical (IHC) analysis of PTOA knees revealed abundant MMP13 in articular cartilage, synovium and meniscus compared with tissues in healthy knees. PTOA-induced MMP13 protein signal was markedly reduced in each PTOA knee compartment at 5 days after treatment with siMMP13<(EG_18_L)_2_ delivered at either 5 or 10 mg kg^−1^. However, neither siMMP13-Chol nor siMMP13 impacted MMP13 levels in PTOA knees (Extended Data Fig. 2d). MMP13 protein was undetectable in liver or kidneys by IHC (Extended Data Fig. 2e). RNA expression analyses similarly did not detect *Mmp13* in liver, although low levels were found in kidney, albeit 6,000-fold lower than that detected in PTOA knees (Extended Data Fig. 2f). Regardless, kidney *Mmp13* gene expression was unaffected in siMMP13<(EG_18_L)_2_-treated mice (Extended Data Fig. 2g), supporting the idea that siMMP13<(EG_18_L)_2_ might have a wide therapeutic window, with minimal risk of molecularly on-target side effects in two of the primary organs commonly associated with siRNA clearance, liver and kidney.

To further characterize cellular sources of MMP13 in PTOA synovium, we analysed single-cell RNA-sequencing from a murine anterior cruciate ligament (ACL) rupture-induced PTOA model^59^. Synovial fibroblasts, specifically *Thy1+ Pdgfra+ Prg4−* sublining fibroblasts were the primary *Mmp13*-expressing cell type in synovium, with some myeloid cells exhibiting a much lower degree of *Mmp13* expression. While healthy synovium was essentially devoid of *Mmp13*-expressing cells, PTOA synovium exhibited a large increase in the number of *Mmp13*-positive synovial fibroblasts (Supplementary Fig. 15). Taken together with Cy5-siRNA<(EG_18_L)_2_ uptake results demonstrating a high degree of uptake by synovial fibroblasts (Fig. 2f and Supplementary Fig. 11), these results suggest that our siRNA<(EG_18_L)_2_ structure efficiently targets the primary *Mmp13*-expressing cells in synovium.

### Sustained retention of siMMP13<(EG_18_L)_2_ in PTOA joints enables potent and durable MMP13 knockdown

**Extended Data Fig. 3 ∣:**
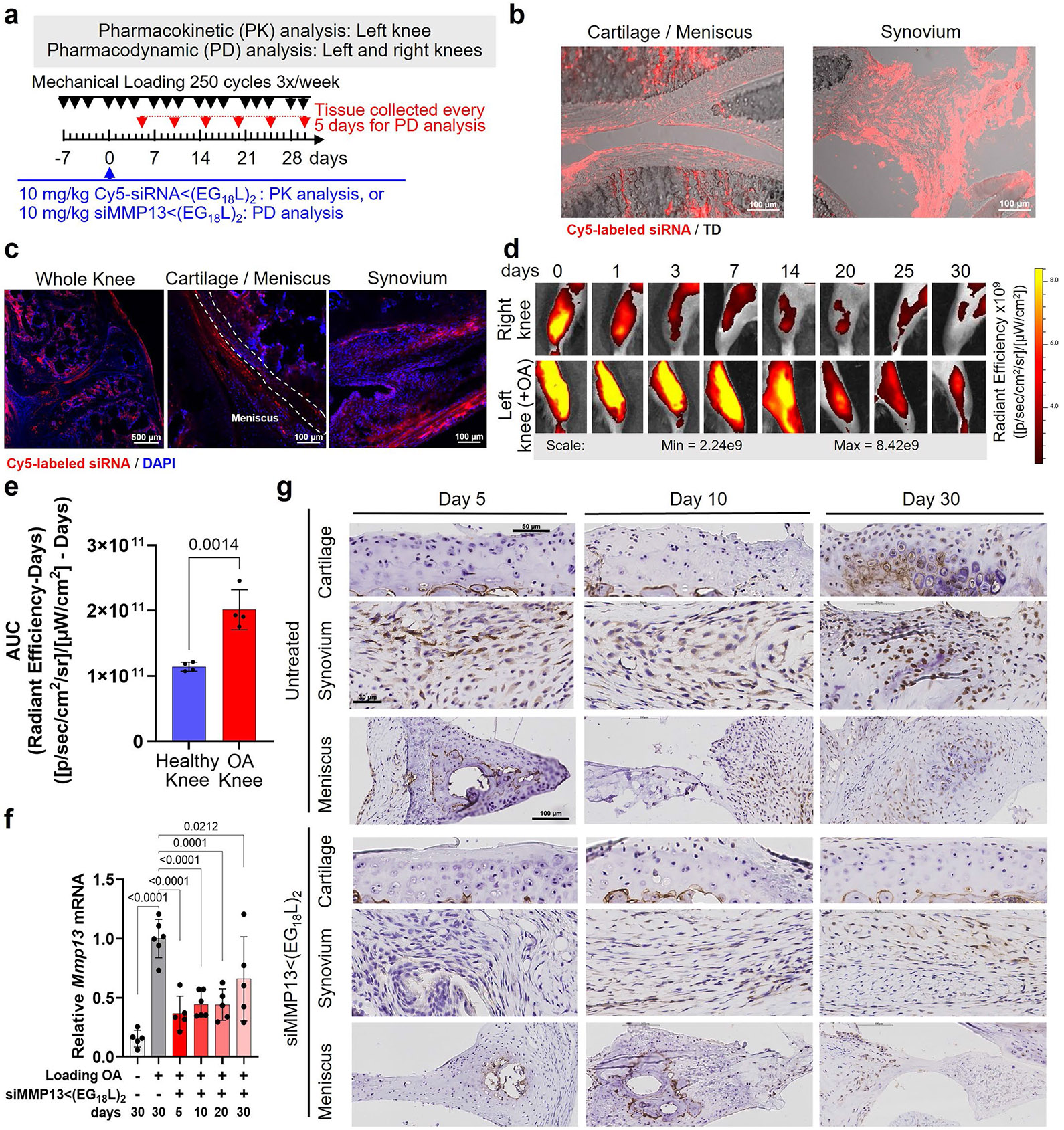
Systemic (i.v.) siMMP13<(EG_18_L)_2_ (10 mg/kg) treatment achieves long-term retention and MMP13 silencing in PTOA knee joints. **a**, Timeline for mouse left knee mechanical loading (3 times / week) and single treatment (day 0; 7 days after initiation of mechanical loading) with Cy5-siRNA<(EG_18_L)_2_ or siMMP13<(EG_18_L)_2_. **b**, Cryosection confocal microscopy imaging of Cy5-siRNA<(EG_18_L)_2_ in joint tissues taken down on day 1 post-treatment. **c-e**, Cy5-siRNA<(EG_18_L)_2_ was visualized on day 30 post-treatment by confocal microscopy of knee joint cryosections (**c**, representative images with dashed lines outlining articular cartilage), and at time points from 0-30 days post-treatment by longitudinal intravital Cy5 imaging (**d**). Semiquantitative analysis of the AUC (Cy5 fluorescence x days) was calculated (**e**) (N = 4). **f-g**, Knee joint *Mmp13* was measured at the indicated time points by qRT-PCR (**e**) and IHC (**f**) in healthy or PTOA knee tissues collected at the indicated time points from mice treated with or without siMMP13<(EG_18_L)_2_. N = 5-6. For all panels: Error bars indicate standard deviation of biological replicates. Knees labeled healthy indicate a non-loaded contralateral limb (right).

Mice were subjected to mechanical left knee loading (3 times per week) over 5 weeks. They were i.v. treated with a single dose of Cy5-siRNA<(EG_18_L)_2_ at 10 mg kg^−1^ after the first week of loading, and longitudinal measurements of Cy5 retention in PTOA knees were performed at time points throughout the next 30 days (Extended Data Fig. 3a). At day 1, there was significant Cy5 signal in the cartilage/meniscus and synovial tissues (Extended Data Fig. 3b) that was retained for up to 30 days in PTOA knees treated systemically with Cy5-siRNA<(EG_18_L)_2_ (Extended Data Fig. 3c), and higher siRNA<(EG_18_L)_2_ levels were detected in PTOA knees over contralateral control knees (Extended Data Fig. 3d,e). Importantly, a single siMMP13<(EG_18_L)_2_ treatment maintained knockdown of MMP13 transcript and protein in PTOA joints through day 30 (Extended Data Fig. 3f,g).

**Extended Data Fig. 4 ∣:**
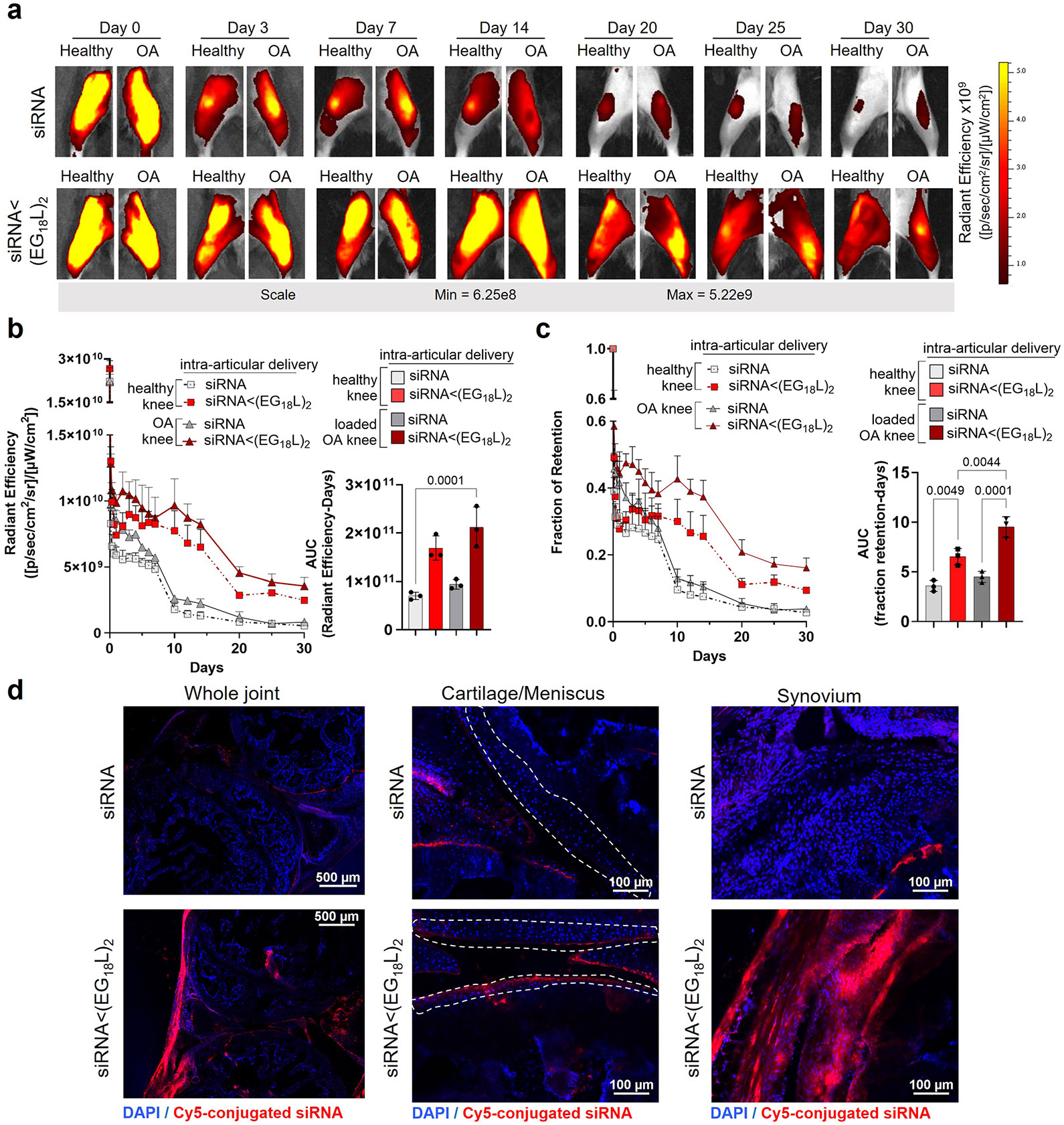
Healthy and PTOA knee joint retention after intra-articular delivery over a 30-day time course. **a**, Representative IVIS images of healthy and PTOA mouse knee joints over 30 days after a single 1 mg/kg intra-articular (i.a.) injection of Cy5-siRNA<(EG_18_L)_2_ or Cy5-siRNA. **b**, Semiquantitative analysis of time-course fluorescent radiant efficiency within healthy and OA mouse knee joints over 30 days (N = 3). AUC was calculated based on the fluorescence intensity profiles. Radiant efficiency over time plotted as mean + SEM. AUC plot error bars indicate standard deviation of biological replicates. **c**, Semiquantitative analysis of time-course fraction of retention within healthy and OA mouse knee joints over 30 days (N = 3). AUC was calculated based on the fraction of retention profiles. Radiant efficiency over time plotted as mean + SEM. AUC plot error bars indicate standard deviation of biological replicates. **d**, Representative confocal microscopy of knee joint cryosections at the 30-day endpoint showing i.a. Cy5-siRNA<(EG_18_L)_2_, and i.a. Cy5-siRNA in loaded, PTOA, knees with a specific focus on synovial and cartilage/meniscus tissues. Dashed lines outline articular cartilage. Knees labeled healthy indicate a non-loaded contralateral limb (right).

Intra-articular dosing of siRNA<(EG_18_L)_2_ (1 mg kg^−1^) was similarly assessed, revealing preferential retention of Cy5-siRNA<(EG_18_L)_2_ in PTOA knees over contralateral healthy knees through 30 days post treatment (Extended Data Fig. 4). In a side-by-side comparison, an i.v. dose of 10 mg kg^−1^ and intra-articular dose of 1 mg kg^−1^ siRNA<(EG_18_L)_2_ had remarkably similar area under the curve (AUC) profiles, suggesting that ~10-fold higher dose should be used for the i.v. relative to the i.a. delivery route for siRNA<(EG_18_L)_2_ arthritic knee joint treatments. These trends were confirmed by a peptide nucleic acid (PNA) hybridization assay that enables absolute siRNA quantification in tissue. This measurement showed preferential accumulation of siRNA<(EG_18_L)_2_, but not free siRNA, in PTOA knees over contralateral healthy knees, and similar siRNA levels (measured as ng siRNA per mg tissue) when delivered at 10 mg kg^−1^ i.v. and at 1 mg kg^−1^ i.a. (Supplementary Fig. 16d). Broader organ biodistribution was also assessed at 30 days following administration of intravenous or intra-articular treatments. While signal remained in all organs, the siRNA<(EG_18_L)_2_ signal was highest within PTOA joints of all the tissues analysed (Supplementary Fig. 16).

### siMMP13<(EG_18_L)_2_ diminishes molecular, histological and clinical manifestations of PTOA

**Fig. 3 ∣:**
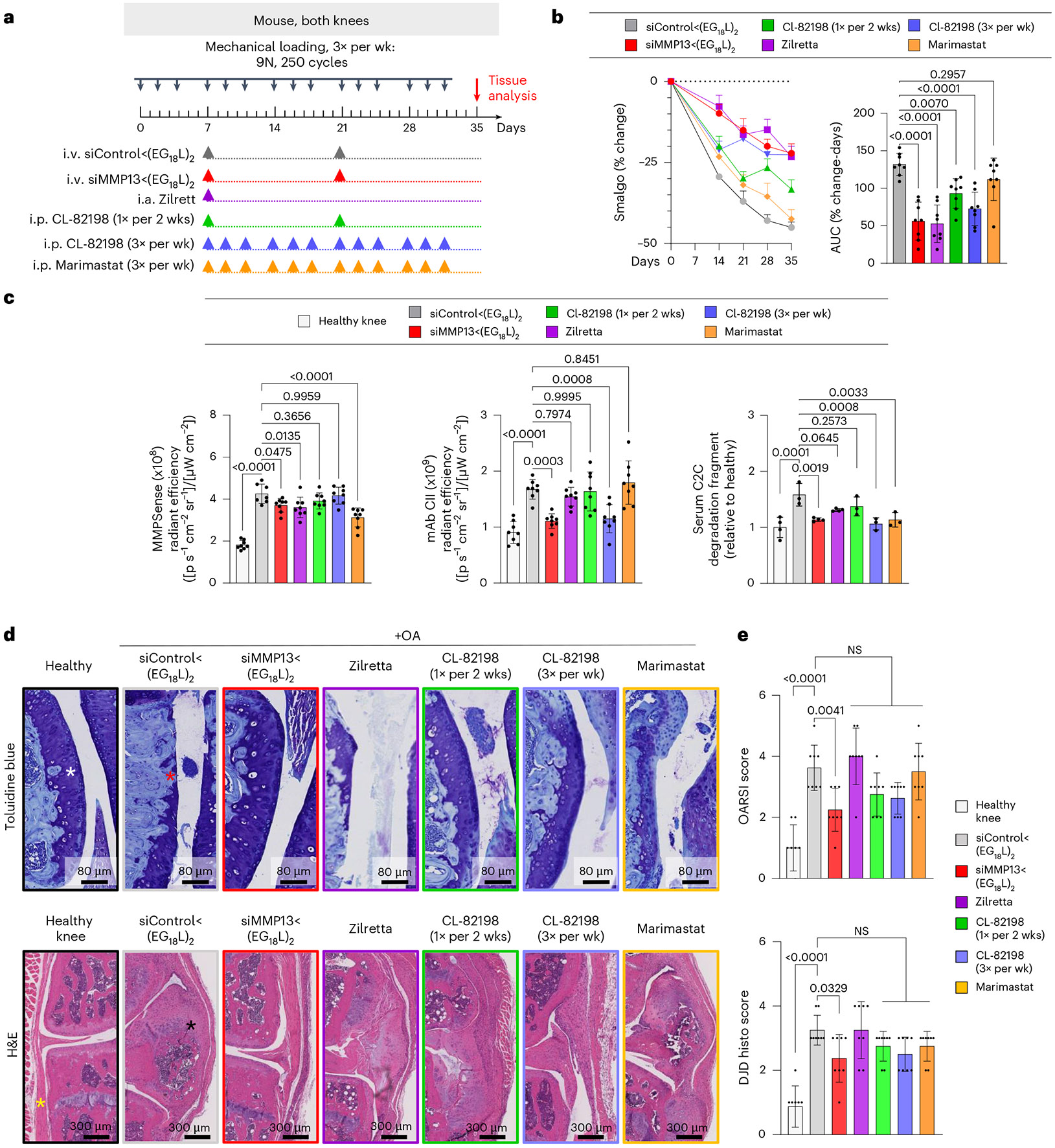
Systemic delivery of siMMP13<(EG_18_L)_2_ decreases PTOA-induced hyperalgesia and joint histopathology. **a**, Schematic timeline for knee loading and treatment. Bilateral knee loading (3× per week, 5 weeks) was used to induce severe PTOA for comparing therapeutic impact of treatment with i.v. siMMP13<(EG_18_L)_2_, i.a. Zilretta, i.p. CL-82198 and i.p. Marimastat. **b**, Left: mechanical hyperalgesia was measured via algometer through day 35. Data over time displayed as mean + s.e.m. Right: AUC of average Smalgo readings over time was assessed. *N* = 8. **c**, Left: total MMP activity in mouse knees was measured using MMPsense 750 Fast (*N* = 8). Middle: mAbCII binding in knee joints was measured by fluorescence imaging (*N* = 8). Right: C2C fragments in mouse serum was measured by ELISA (*N* = 3). **d**, Knee joints were stained with toluidine blue (top row, femoral condyles shown) and H&E (bottom row, synovium lining and meniscus shown). Asterisks: white, healthy articular surface; red, loss of articular surface; yellow, healthy synovial lining/meniscus; black, synovial thickening/meniscal expansion and calcification. **e**, Joint cartilage damage was quantitated with the OARSI osteoarthritis cartilage histopathology assessment system (top) and DJD score (bottom). NS, not significant. In all panels, error bars indicate s.d. of biological replicates, unless specified otherwise.

Therapeutic efficacy of siMMP13<(EG_18_L)_2_ was tested in a mouse PTOA model after 5 weeks of mechanical knee loading (3 times per week). Mice were treated with siMMP13<(EG_18_L)_2_ or siControl<(EG_18_L)_2_ (10 mg kg^−1^) on day 7 and again on day 21 (Fig. 3a). The pressure-pain threshold in PTOA knees was measured by algometer, revealing a 45% reduction in threshold pressure tolerance in siControl<(EG_18_L)-treated mice, while siMMP13<(EG_18_L)_2_ lost only 25% (Fig. 3b). The siMMP13<(EG_18_L)_2_ cohort showed relief of joint sensitivity to a level similar to mice receiving intra-articular delivery of the FDA-approved sustained release corticosteroid Zilretta (8 mg kg^−1^) or 3× per week intraperitoneal treatment with the experimental MMP13-selective small-molecule inhibitor CL-82198 (ref. 19) (10 mg kg^−1^) (Fig. 3a,b). However, treatment with CL-82198 on the same schedule as siMMP13<(EG_18_L)_2_ (once every 2 weeks) produced a less-favourable pain tolerance in PTOA knees, as did treatment with the pan-MMP inhibitor Marimastat (10 mg kg^−1^ i.p., 3× weekly).

Pan-MMP activity in PTOA joints was assessed in vivo on study day 30 using MMPSense, an MMP substrate that fluoresces upon proteolytic cleavage. Intravital imaging revealed significantly elevated MMP-driven fluorescence in PTOA knees of mice treated with siControl<(EG_18_L)_2_ compared with healthy knees (Fig. 3c). Decreased pan-MMP activity was seen in PTOA knees of mice treated with siMMP13<(EG_18_L)_2_, Zilretta and Marimastat, although the MMP13-selective small-molecule inhibitor CL-82198 did not significantly diminish pan-MMP activity in PTOA knees. A fluorescently labelled monoclonal antibody against aberrantly exposed CII collagen (mAbCII) was used to assess relative cartilage damage^60-62^, finding significantly elevated mAbCII localization to PTOA knees (Fig. 3c), thus confirming PTOA-induced cartilage damage in this model. However, mAbCII intensity in PTOA knees was diminished significantly in siMMP13<(EG_18_L)_2_-treated samples, approximating the mAbCII signal in healthy knees. Similar trends were observed for serum-based measurements of collagen C2C degradation fragments, a biomarker of cartilage degradation^63^. PTOA-induced mAbCII signal and serum C2C levels were also diminished by CL-82198 when dosed with higher frequency (3 times per week), underscoring the causative role of MMP13 in PTOA-induced collagen fragmentation and cartilage damage and supporting the hypothesis that MMP13 inhibitory strategies reduce structural OA manifestations. The long-acting steroid Zilretta had little impact on mAbCII binding in PTOA knees or on serum C2C levels, consistent with observations that steroids temporarily control arthritis pain but do not target underlying joint destruction and may even accelerate cartilage thinning^64^.

Histological analyses of PTOA joints collected on study day 30 revealed profound disruption of cartilage architecture, proteoglycan loss, synovial thickening, osteophyte formation and meniscal mineralization (Fig. 3d and Supplementary Fig. 20). In PTOA knees treated with siMMP13<(EG_18_L)_2_, cartilage morphology, proteoglycan content, synovial structure and meniscal anatomy were each relatively maintained, as quantitated using the Osteoarthritis Research Society International (OARSI) OA severity scoring system^65,66^ (Fig. 3e and Supplementary Table 1). Degenerative joint disease scoring (Supplementary Table 1) also indicated that there were significantly less structural changes in the mechanically loaded joints of mice treated with siMMP13<(EG_18_L)_2_ versus those treated with non-targeting siControl<(EG_18_L)_2_. Unlike what was seen with siMMP13<(EG_18_L)_2_, modest reductions in OA progression and joint degeneration were achieved in PTOA joints treated with CL-82198, although these trends did not reach statistical significance. OA progression and joint degeneration scores were not impacted by treatment with Zilretta or Marimastat.

**Extended Data Fig. 5 ∣:**
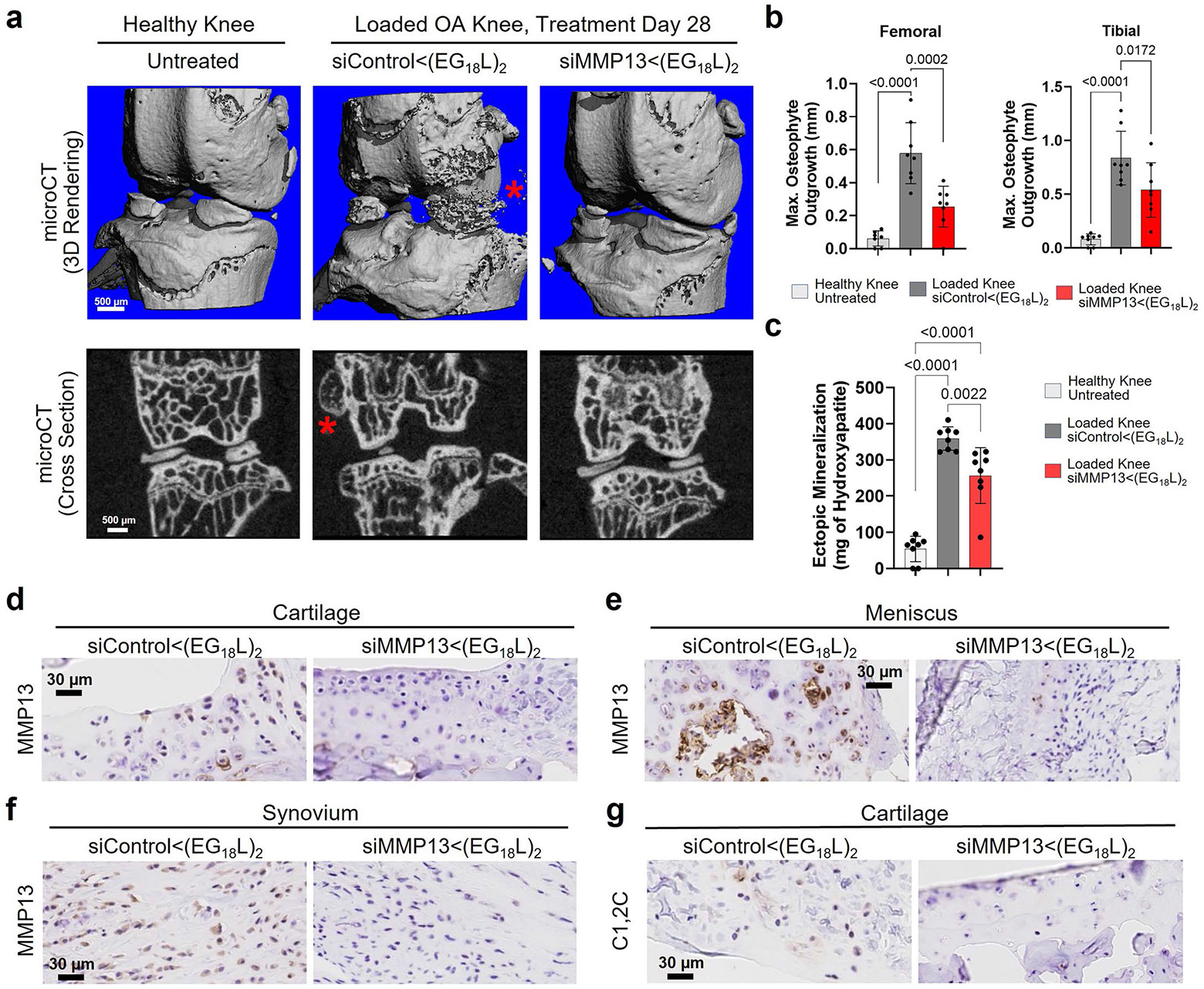
Intravenous albumin-hitchhiking siMMP13<(EG_18_L)_2_ treatment provides whole knee joint protection by reducing osteophyte formation and pathologic mineralization and is associated with a reduction in MMP13 protein and collagen degradation fragments. **a**, MicroCT-based 3-dimensional (3D) renderings of meniscal/ectopic mineralization and osteophyte growth in healthy and PTOA mice treated with siMMP13<(EG_18_L)_2_ or siControl<(EG_18_L)_2_. Red asterisk = osteophyte outgrowth. **b**, Measurements of femoral and tibial osteophyte size at largest outgrowth from normal cortical bone structure. N = 8. **c**, Ectopic mineralization (measured as mg of hydroxyapatite) in menisci and in the form of osteophytes was quantified. N = 8. **d-f**, MMP13 IHC analyses of PTOA joint cartilage (**d**), meniscus (**e**), and synovium (**f**) show reduced MMP13 protein levels in animals treated with siMMP13<(EG_18_L)_2_. **g**, IHC on C1,2 C collagen 2 degradation fragments. Samples here are from the PTOA therapeutic study in Main Fig. 3. For panels **b**-**c**: Error bars indicate standard deviation of biological replicates.

Using microcomputed tomography (microCT) to quantify osteophytes and ectopic mineralization in PTOA mouse knees, the significant protection conferred by siMMP13<(EG_18_L)_2_ against meniscal mineralization and osteophyte formation was confirmed (Extended Data Fig. 5a-c). Importantly, pathologically elevated MMP13 expression in PTOA cartilage, meniscus and synovial compartments was reduced by treatment with siMMP13<(EG_18_L)_2_, correlating with decreased presence of degradation fragments (Extended Data Fig. 5d-g). These data show that MMP13 silencing in arthritic joints provides both molecular and clinically tangible benefits, reducing inflammation, clinical pain, articular cartilage loss and secondary manifestations of OA.

### MMP13 knockdown in PTOA knees reprograms pro-inflammatory gene expression patterns

**Extended Data Fig. 6 ∣:**
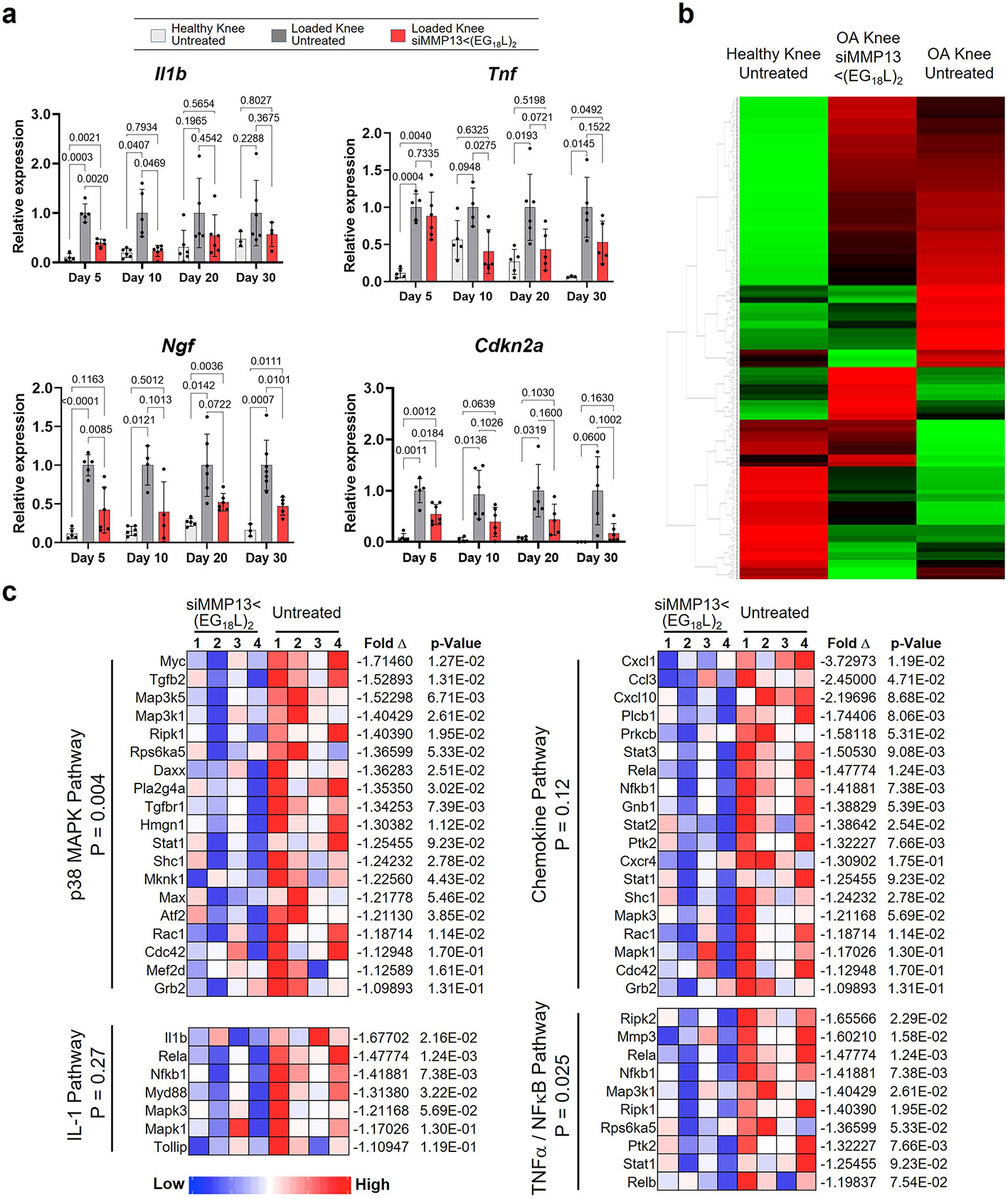
Systemic siMMP13<(EG_18_L)_2_ delivery alters inflammatory gene expression profiles in mechanically-loaded PTOA joints. **a**, qRT-PCR of IL-1B, TNFalpha, NGF, and P16INK4a (Cdkn2a) at days 5, 10, 20, and 30 after a single i.v. injection of 10 mg/kg siMMP13<(EG_18_L)_2_. Analyzed using a mixed effects analysis. N = 3-7. Error bars indicate standard deviation. **b**, Unsupervised sorting of treatment groups as quantified by nanoString at day 10 after treatment of PTOA knees with 10 mg/kg siMMP13<(EG_18_L)_2_. Gene expression is shown as high- (green) or low-expression (red) sorted vertically by differences between treatment groups. **c**, Gene cluster expression changes at day 10 post-treatment in PTOA knees.

To understand the gene expression patterns that underlie the therapeutic outcomes associated with MMP13 silencing, we first measured the impact of PTOA on a small panel of pro-inflammatory genes with previously described roles in OA, including *Ngf*, *Il1b*, *Tnf* and *Cdkn2a*. These genes were each elevated in mouse PTOA knees compared with healthy knees (Extended Data Fig. 6a). We found that PTOA-induced levels of *Ngf*, *Il1b*, *Tnf* and *Cdkn2a* were significantly dampened as early as 5 days following i.v. delivery of siMMP13<(EG_18_L)_2_ at 10 mg kg^−1^, gene expression changes that were sustained through day 30. These findings motivated a broader and unbiased analysis of PTOA-induced gene expression changes in knee tissue, along with measuring the broader gene expression impact of MMP13 knockdown. Using a nanoString nCounter Inflammation 254 gene panel, unsupervised analysis of gene expression illustrated extensive PTOA-induced gene expression changes (Extended Data Fig. 6b). Notably, PTOA induction altered signalling along the pathway driven by p38 mitogen associated protein kinase (MAPK), a serine-threonine kinase that phosphorylates a network of substrates that respond to cell stress^67^. Relevant to OA, p38 MAPK signalling through its substrate MAPK-associated protein kinase 2 (MK2) regulates chondrocyte differentiation, cellular senescence, MMP upregulation and pro-inflammatory cytokine production^68^. MK2 signalling within human arthritic chondrocytes drives expression of prostaglandin E2, MMP3 and MMP13, leading to arthritic pain, inflammation and cartilage degradation^69^. Interestingly, we found that the PTOA-induced p38 MAPK gene cluster was the gene enrichment set most potently suppressed in siMMP13<(EG_18_L)_2_-treated samples (Extended Data Fig. 6c).

Treatment with siMMP13<(EG_18_L)_2_ also significantly suppressed IL-1, chemokine and TNF/NFκB pathway-associated gene clusters (Extended Data Fig. 6c). Notably, these clusters include several genes associated with OA pathogenesis, including those associated with chondrocyte proliferation (*Mef2d*), synovial fibroblast proliferation (*Myc*, *Jun*, *Tgfb2*, *Pdgfa*)^70,71^, angiogenesis (*Pdgfba*, *Flt1*, *Hif1a*), ECM degradation (*Mmp3, Masp1*), inflammatory signalling through nuclear factor-κB, (*Nfκb1*, *Rela*, *Relb*), cytokines (*Il1b*, *Stat1*), chemokines (*Cxcl1*, *Cxcl10*, *Ccl3*, *Cxcr4*) and toll-like receptors (*TLR1*, *2*, *4*, *5*, *6 and 8*).

Given that many inflammatory and stress response factors induce MMP13 expression and that proteolytic matrix degradation fragments further potentiate inflammation and expression of stress response factors^72^, these data suggest that siMMP13<(EG_18_L)_2_ uncouples a feed-forward inflammation-degradation cycle that drives arthritic joint pathology. These data support this hypothesis, given that selective MMP13 inhibition in arthritic joints suppresses expression of both pro-inflammatory factors and transcription factors that promote MMP13 expression.

### K/BxN serum transfer arthritis (STA) multijoint rheumatoid/inflammatory arthritis mouse model

**Extended Data Fig. 7 ∣:**
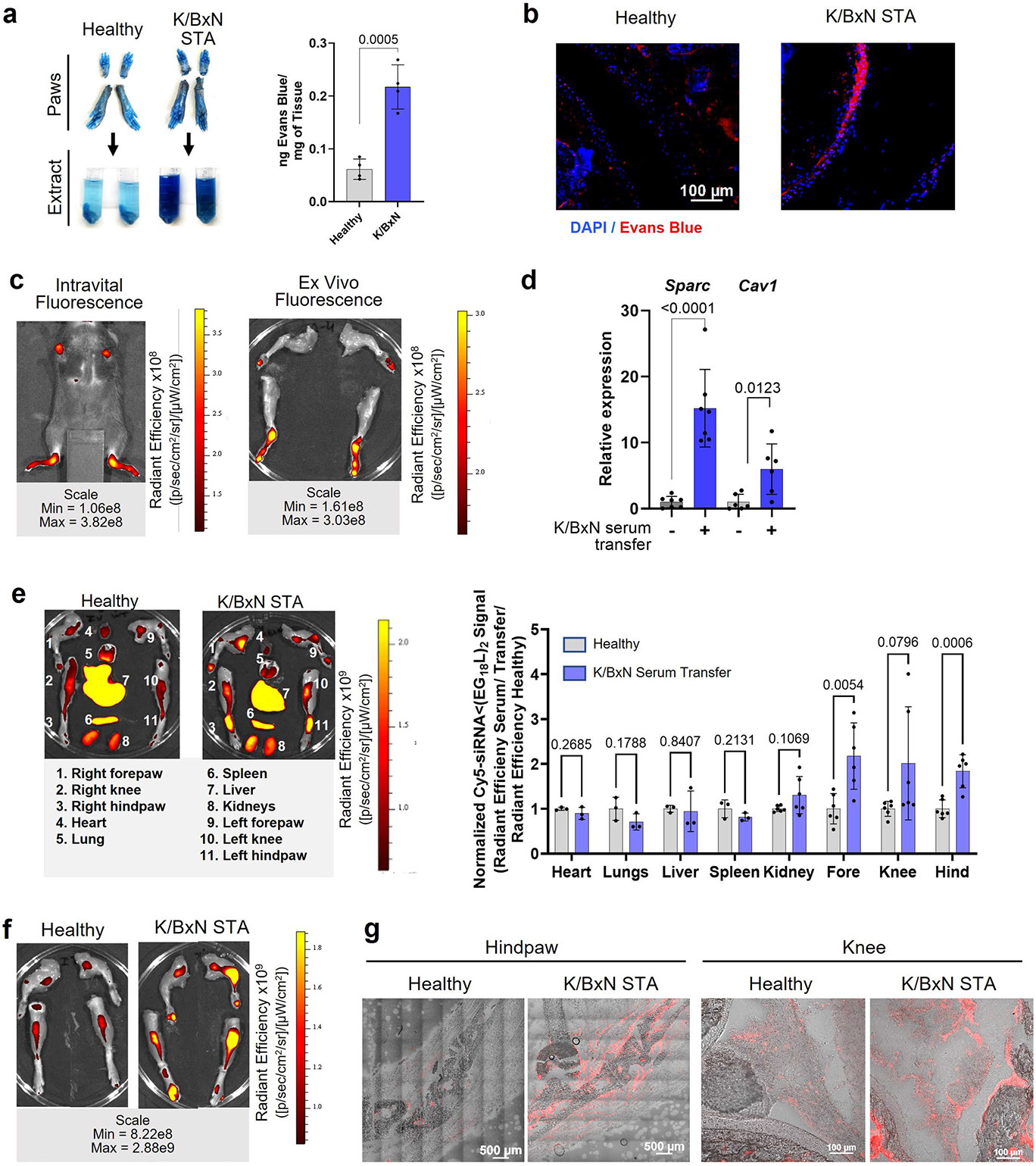
Characterizing albumin-based drug delivery in the K/BxN serum transfer arthritis model. **a-c**, Evans Blue or Cy5-MSA was delivered i.v. to wild type (healthy) and K/BxN serum recipient mice 4 days after serum transfer. Joints of forepaws and hindpaws were used for Evans Blue extraction (**a**, representative images), measuring Evans Blue absorbance in extracts (**a**, right). Evans Blue was also visualized by fluorescence microscopy in joint cryosections (**b**). MSA-Cy5 delivery to joints was visualized by intravital Cy5 fluorescence imaging (**c**). **d**, qRT-PCR was used to measure relative gene expression of albumin transport-associated genes (Sparc and Caveolin-1 [Cav1]) in healthy and K/BxN arthritis hind paws. N = 7 for Sparc; N = 6 for Cav1. **e-g**, Cy5-siRNA<(EG_18_L)_2_ (1 mg/kg, i.v.) was delivered to healthy and K/BxN serum recipient mice. Cy5 fluorescence in organs (**e**) and limb joints (**f**), analyzed using multiple unpaired t-tests with no correction for multiple comparisons, were measured ex vivo at 24 hrs. Cy5 fluorescence was assessed in knee and hindpaw cryosections by fluorescence microscopy (**g**). For panel **e**, N = 3 for heart, lungs, liver, and spleen; N = 6 for kidney, forepaw, knee, and hindpaw. For all panels: Error bars indicate standard deviation of biological replicates.

Rheumatoid arthritis is a systemic, multifactorial autoimmune disease that causes painful synovial inflammation and cartilage degradation in multiple joints simultaneously^73^. Increased MMP13 activity and consequent cartilage loss is a known feature of RA, and daily treatment with an experimental MMP13 small-molecule inhibitor can reduce RA pathogenesis^4,74-76^. Thus, we hypothesized that siMMP13<(EG_18_L)_2_ therapy would reduce cartilage loss, decrease induction of pro-inflammatory gene expression and suppress multiple features of RA. We used the transgenic K/BxN serum transfer model to test therapeutic efficacy of siMMP13<(EG_18_L)_2_ in an RA-like scenario. In this model, KRN, a T-cell receptor that binds to an endogenous glucose-6-phosphate isomerase (GPI) peptide presented by the IAg7 major histocompatibility complex class II (MHC-II) allele, drives production of anti-GPI autoantibodies and GPI-directed autoimmunity that localizes to articular cartilage/joint tissues. K/BxN mouse serum transiently confers RA to wild-type mouse serum recipients. MMP13 expression is increased in inflamed joints of K/BxN serum recipients, and MMP13-deficient mice are partially protected from K/BxN serum-induced RA^76^. Carrier-free siRNA delivery in RA has not yet been explored to our knowledge, but siMMP13<(EG_18_L)_2_ systemic administration for delivery to multiple afflicted joints is potentially advantageous. Interestingly, albumin-based delivery approaches have been shown to facilitate therapeutic outcomes in RA preclinical studies^30,31,48,77-84^. Importantly, i.v. delivery of albumin-binding Evans blue to mice resulted in concentrated Evans blue accumulation in arthritic forepaws and hindpaws of K/BxN serum recipients compared with unchallenged mice (Extended Data Fig. 7a,b). Likewise, i.v. delivery of Cy5-labelled MSA led to accumulation in arthritic forepaws and hindpaws of K/BxN serum recipients, but not in unchallenged wild-type mice (Extended Data Fig. 7c). Expression of caveolin-1 and SPARC was elevated in hindpaws of K/BxN recipient mice (Extended Data Fig. 7d). These data confirm increased albumin retention in RA-affected joints, supporting therapeutic testing of albumin-binding siRNA<(EG_18_L)_2_ in RA models.

**Fig. 4 ∣:**
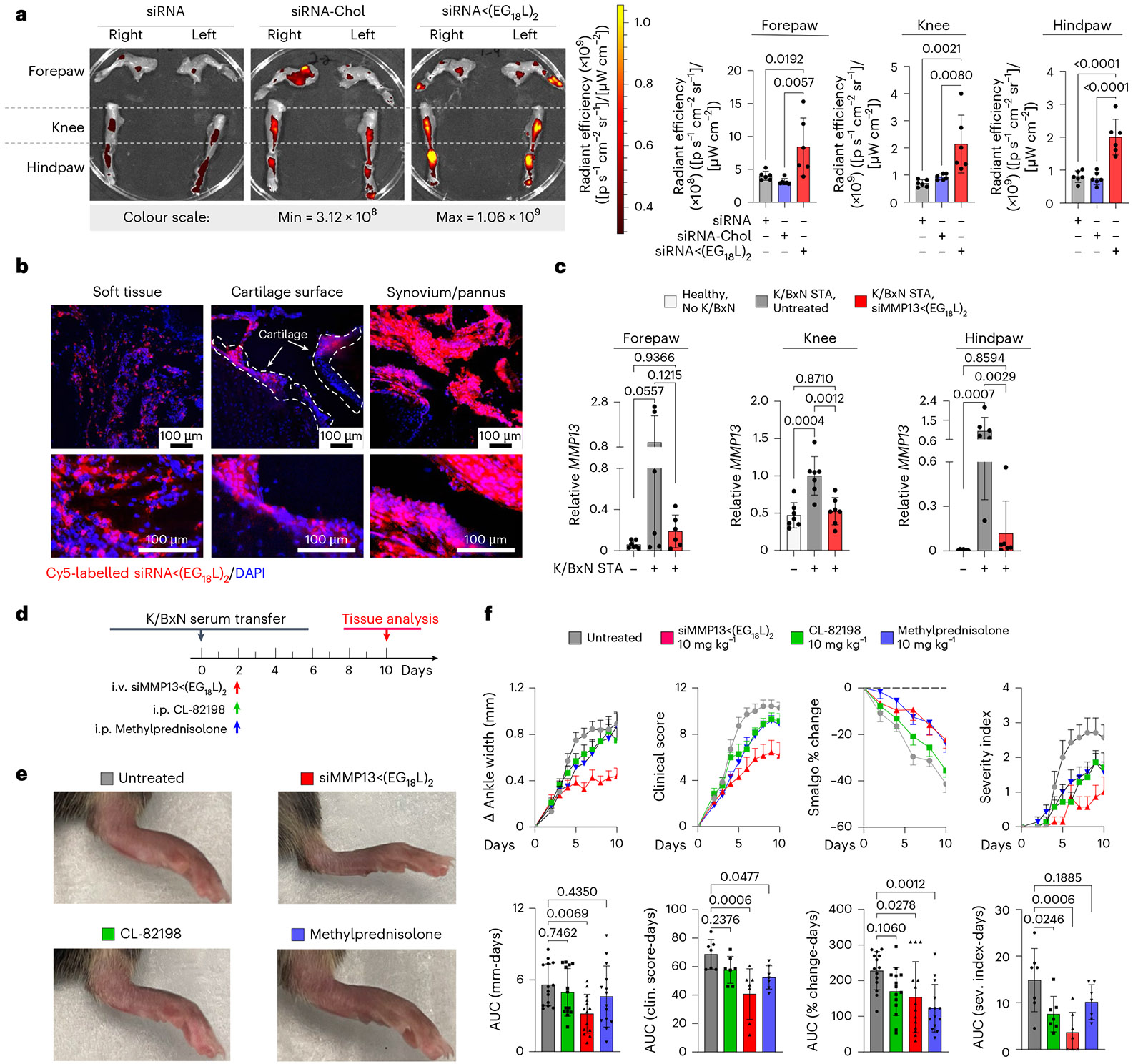
Intravenous delivery of siMMP13<(EG_18_L)_2_ achieves multijoint accumulation, MMP13 knockdown and diminished arthritis progression in a mouse RA model. **a**,**b**, In a mouse inflammatory arthritis (K/BxN serum transfer) model, mice were treated with Cy5-siRNA<(EG_18_L)_2_, Cy5-siRNA-Chol and Cy5-siRNA (1 mg kg^−1^ i.v.) 4 days after receiving K/BxN serum transfer. Joints of forepaw, hindpaw and knee were collected 24 h later and assessed by IVIS imaging (**a**) or fluorescence microscopy of hindpaw ankle joints (**b**). Representative images and quantitation of IVIS data are shown. *N* = 6. Dashed lines outline articular cartilage in fluorescence microscopy images of tissue cryosections. **c**, Relative *Mmp13* mRNA in forepaws, knees and hindpaws of healthy untreated wild-type mice or in K/BxN serum recipients treated with siControl<(EG_18_L)_2_ or siMMP13<(EG_18_L)_2_ (10 mg kg^−1^ i.v.) was measured by RT–qPCR. *N* = 6. Error bars represent s.d. of biological replicates. **d**–**f**, K/BxN serum recipients were treated with siMMP13<(EG_18_L)_2_, methylprednisolone or CL-82198 (10 mg kg^−1^). **d**, Schematic timeline for K/BxN serum transfer and treatment. **e**, Photos of hindpaws were collected on treatment day 10, and representative images are shown. **f**, Ankle width change, clinical score, algometer ankle joint hyperalgesia and severity index were measured for 10 days after treatment. Error bars indicate s.e.m. (top) or s.d. (bottom) of biological replicates.

Mice with STA showed accumulation of i.v.-injected Cy5-siRNA <(EG_18_L)_2_ within multiple inflamed joints (forepaw, wrist, hindpaw, ankle, knee) (Fig. 4a and Supplementary Fig. 17) at substantially higher levels than what was seen in healthy mice (Extended Data Fig. 7e-g) and exceeding that achieved by treatment with Cy5-siRNA or Cy5-siRNA-Chol (Fig. 4a). Fluorescence microscopy confirmed increased siRNA<(EG_18_L)_2_ in fore- and hindpaws of K/BxN recipients, with specific accumulation in soft tissue and cartilage (Fig. 4b). Longevity of siRNA retention was assessed in limbs of K/BXN serum recipients following a single-dose Cy5-siRNA<(EG_18_L)_2_. Intravital fluorescence was detected in limbs throughout the 30 days of mice monitoring (Supplementary Fig. 17c), supporting the notion of albumin-mediated delivery and retention of siRNA<(EG_18_L)_2_ in multiple afflicted joints in RA models.

### siMMP13<(EG_18_L)_2_ therapy reduces manifestations and symptoms of RA

**Extended Data Fig. 8 ∣:**
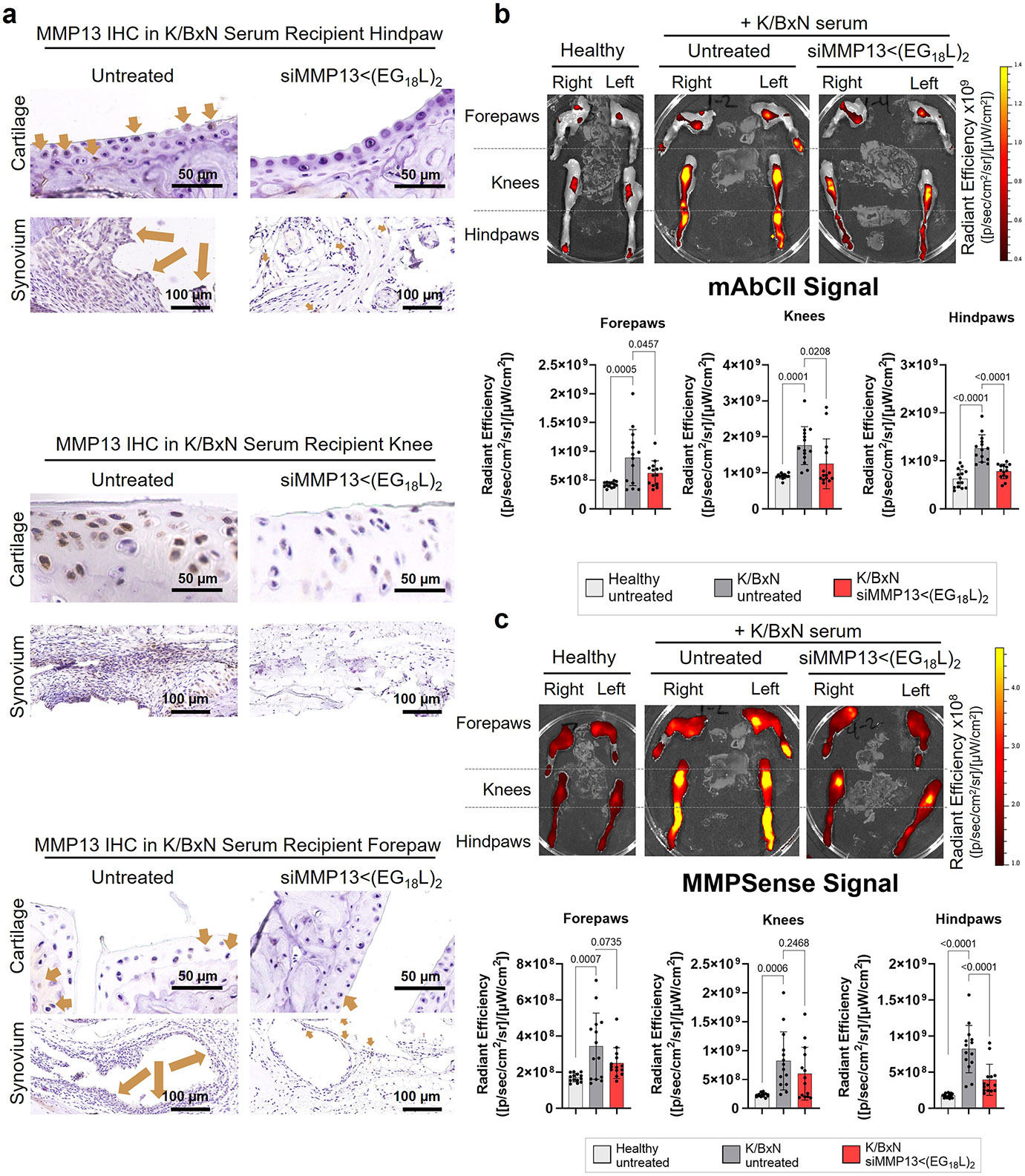
Intravenous siMMP13<(EG_18_L)_2_ delivery silences MMP13 and protects cartilage in the joints of mice given K/BxN serum transfer. K/BxN STA mice were treated with or without siMMP13<(EG_18_L)_2_ (10 mg/kg, i.v.), then assessed at day 10. **a**, MMP13 IHC analysis of cartilage and synovium in multiple joints was used to assess silencing of MMP13 expression. **b**, Fluorescent mAbCII delivered to mice was detected by ex vivo fluorescence imaging to measure relative cartilage damage in healthy and K/BxN STA mice undergoing treatment (representative images shown). N = 14. **c**, Pan-MMP activity was measured via delivery of MMPsense 750 Fast to mice, followed by detection using ex vivo fluorescence imaging. N = 14. For all panels: Error bars indicate standard deviation of biological replicates.

Strong *Mmp13* induction was seen in forepaws, knees and hindpaws of K/BxN serum recipients compared with healthy mice (Fig. 4c). At treatment day 8 following a single 10 mg kg^−1^ dose of siMMP13<(EG_18_L)_2_, mRNA and protein were decreased in forepaws, knees and hindpaws (Fig. 4c and Extended Data Fig. 8a). Similar to PTOA, MMP13 silencing in this RA model reduced expression of pro-inflammatory genes and cytokines encoding cyclooxygenase 2 (COX2), IL-6, TNF and IL-1β (Supplementary Fig. 18). Clinical parameters of RA were also reduced upon MMP13 knockdown, including ankle swelling, pressure-pain threshold, arthritis clinical score and disease severity index (Fig. 4d-f), and the number of afflicted joints per mouse was decreased (Supplementary Fig. 19). In parallel studies, K/BxN serum recipients were treated with a single dose of Cl-82198 (10 mg kg^−1^ i.p.) or methylprednisolone (10 mg kg^−1^ i.p.), neither of which improved ankle swelling (Fig. 4d-f). Although methylprednisolone, but not CL-82198, improved clinical score and pressure-pain tolerance, the effect size was not as pronounced as what was seen with siMMP13<(EG_18_L)_2_. Similarly, the disease severity score was somewhat improved upon treatment with CL-82198, but to a lesser extent than what was seen with siMMP13<(EG_18_L)_2_. Further, MMP13 silencing resulted in decreased mAbCII binding, indicative of cartilage preservation (Extended Data Fig. 8b) and decreased total MMP activity (Extended Data Fig. 8c).

**Extended Data Fig. 9 ∣:**
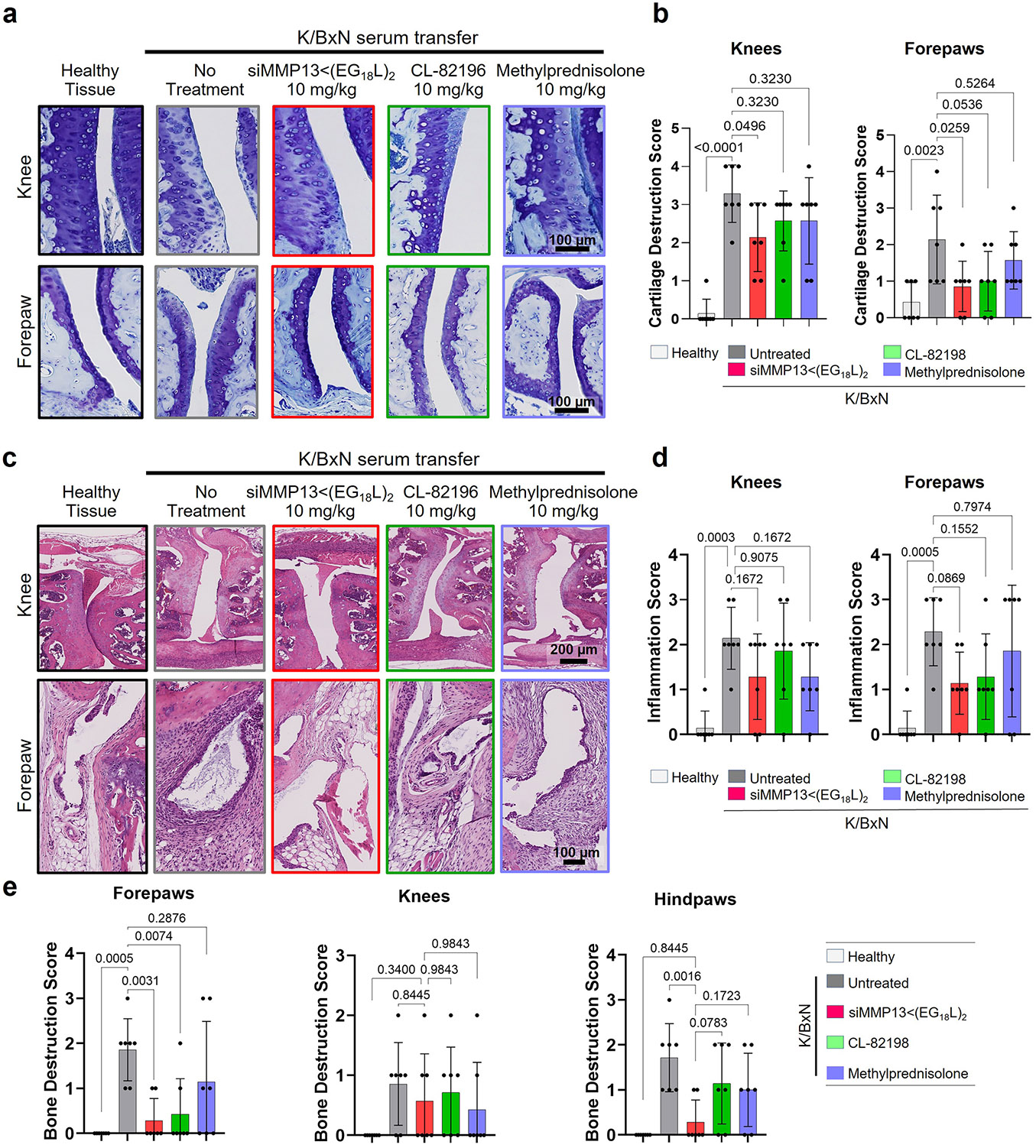
Intravenous delivery of siMMP13<(EG_18_L)_2_ protects cartilage and bone while reducing inflammation in multiple joints of the K/BxN STA model. **a-b**, Toluidine Blue stained histological knee and forepaw sections (**a**) were scored using the Cartilage Destruction Score (**b**). Hindpaw data is shown in Fig. 5. **c-d**, H&E-stained histological knee and forepaw sections (**c**) were scored using the Inflammation Score (**d**). Hindpaw data is shown in Fig. 5. **e**, Histological bone destruction score quantification of hindpaw, knee, and forepaw joint tissues. For panels **b**, **d**, and **e**: N = 7. Error bars indicate standard deviation of biological replicates.

**Extended Data Fig. 10 ∣:**
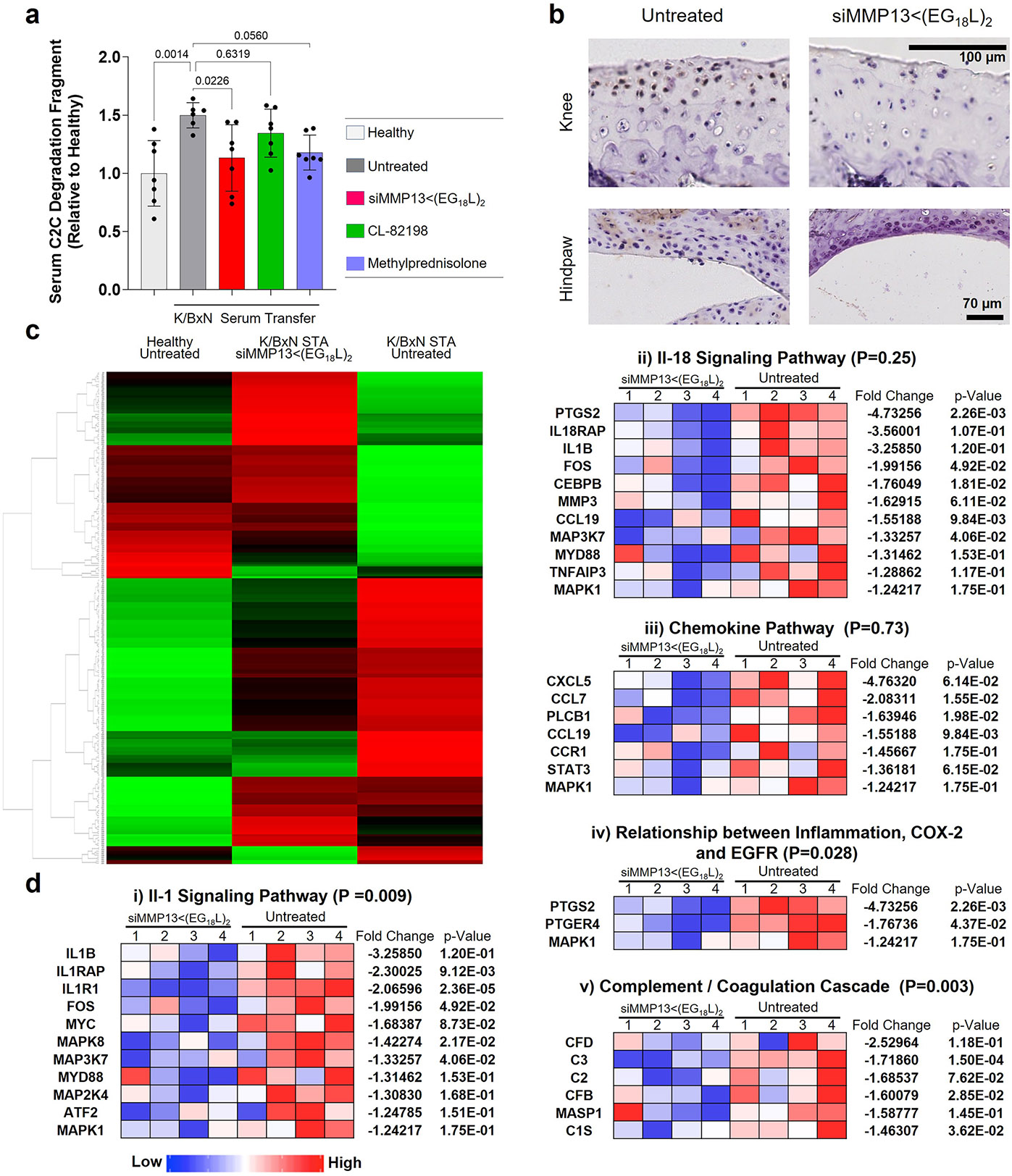
Intravenous siMMP13<(EG_18_L)_2_ treatment reduces presence of cartilage degradation fragments in serum and diminishes the inflammatory gene expression profile in the joints of the K/BxN STA model. **a**, Relative serum C2C collagen 2 degradation fragment levels in serum were measured on day 10 following treatment by ELISA. Error bars indicate standard deviation of biological replicates. N = 6-7. **b**, Presence and localization of C1,2C collagen 2 degradation fragments were assessed by IHC on day 10 post-treatment. **c-d**, RNA harvested from K/BxN recipient mouse joints was assessed by the nanoString nCounter Mouse Inflammatory 294 Gene Expression panel. Clustering of gene expression changes [high- (green) or low- (red)] sorted vertically by differences between treatment groups (**c**). Analysis of relevant gene clusters from tissues that were harvested on day 10 post-treatment (**d**). N = 4.

**Fig. 5 ∣:**
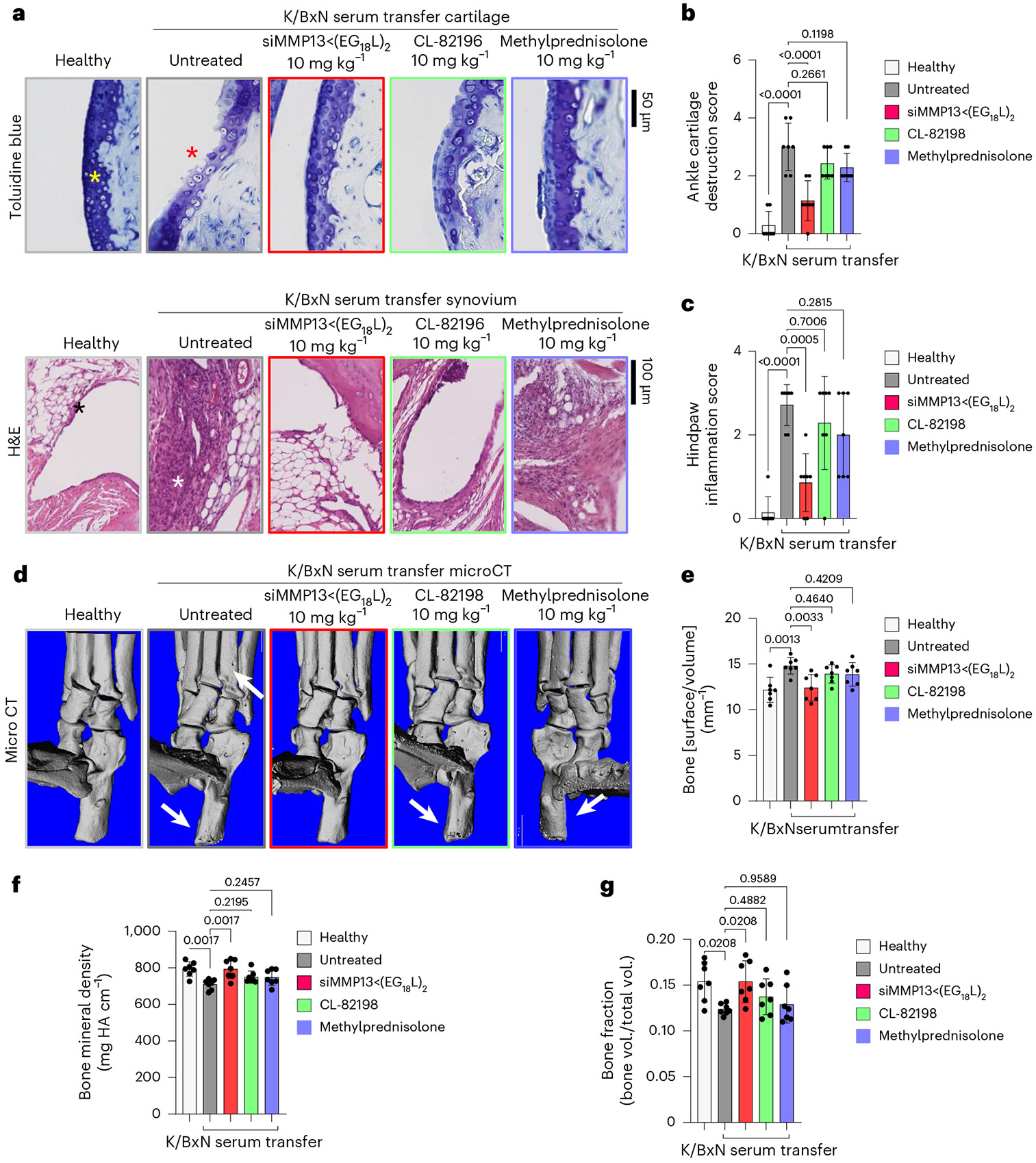
Intravenous siMMP13<(EG_18_L)_2_ treatment protects against cartilage/bone destruction and synovial inflammation in a mouse RA model. K/BxN serum recipient mice were treated with siMMP13<(EG_18_L)_2_, methylprednisolone or CL-82198 (10 mg kg^−1^). **a**–**c**, Histological analysis of toluidine blue-stained ankle cartilage sections (**a**, top) and H&E-stained hindpaw sections (**a**, bottom) were used for cartilage destruction scoring (**b**) and inflammation scoring (**c**). Asterisks: yellow, articular surface; red, articular surface damage; black, healthy synovium; white, synovial inflammation. **d**–**g**, MicroCT-based 3D renderings of hindpaw scans (**d**, representative images) were used to measure bone surface:volume ratio (**e**), bone mineral density (**f**) and bone fraction (**g**). HA, hydroxyapatite, White arrows in **d** show bone erosions. All error bars indicate s.d. of biological replicates.

Histologic examination of toluidine blue-stained cartilage collected on treatment day 8 in K/BxN recipient mice revealed substantial cartilage destruction in hindpaws, knees and forepaws, a phenotype that was diminished by siMMP13<(EG_18_L)_2_ treatment (Fig. 5a,b and Extended Data Fig. 9a,b; lower magnification images in Supplementary Figs. 20b and 21-23). Inflammation scoring illustrated similar trends (Fig. 5c and Extended Data Fig. 9c,d). MicroCT studies of bone structure in untreated K/BxN serum recipients revealed erosive bone disease characterized by increased bone surface to volume ratios, decreased bone mineral density (BMD) and decreased bone volume/total volume (BV/TV) ratio (Fig. 5d-g and Supplementary Fig. 23). Remarkably, treatment with siMMP13<(EG_18_L)_2_ protected K/BxN serum recipients from inflammation-induced changes in bone surface to volume, BMD and BV/TV measurements. Further, bone erosions characteristic of inflammatory arthritis were evident in forepaws, knees and hindpaws of untreated RA mice, but were minimal or absent in RA mice treated with siMMP13<(EG_18_L)_2_ (Extended Data Fig. 9e). In contrast, Cl-82198 and methylprednisolone did not diminish features of erosive bone disease detected by microCT. Serum C2C degradation fragments were significantly elevated in untreated, CL-82198-treated and methylprednisolone-treated K/BxN serum recipients (Extended Data Fig. 10a), while treatment with siMMP13<(EG_18_L)_2_ significantly reduced serum C2C fragments. These findings were confirmed using IHC for C1,2C in K/BxN serum recipients’ hindpaw/ankle and knee-joint tissues (Extended Data Fig. 10b).

Hindpaw ankle articular cartilage and synovial RNA was collected from K/BxN serum transfer recipients on treatment day 8 and assessed using the nanoString nCounter mouse inflammation expression panel. These studies identified several gene clusters that were elevated in untreated K/BxN serum transfer recipients compared with healthy mice, many of which were downregulated in samples collected from K/BxN serum transfer recipients treated with siMMP13<(EG_18_L)_2_ (Extended Data Fig. 10c). Treated RA animals had gene expression patterns that mirrored the changes seen in PTOA knees upon MMP13 knockdown (for example, IL-1 signalling, NF-kB signalling and chemokine pathways were suppressed by treatment) (Extended Data Fig. 10d). In addition, the pro-inflammatory COX2-encoding gene *Ptgs2*, the chemokine genes *Cxcl5, Ccl7* and *Ccl19*, and the RA-associated macrophage genes *Tnfsf14* and *Tr2* were significantly reduced in K/BxN hindpaws treated with siMMP13<(EG_18_L)_2_. Thus, the molecular markers of inflammation that characterize RA are dampened by MMP13 knockdown achieved by i.v. delivery of siMMP13<(EG_18_L)_2_.

Importantly, serum levels of toxicity markers, including blood urea nitrogen, alanine transaminase, aspartate amino transferase, creatine phosphokinase, lactate dehydrogenase and glucose, were detected within the normal ranges, lacking significant differences between healthy, PTOA or K/BxN serum recipient mice in any of the treatment groups (Supplementary Fig. 24). Liver, kidney, lung, heart and spleen of mice within all groups also showed a normal gross and histological structure (Supplementary Fig. 25).

### MMP13 silencing in a Dunkin Hartley guinea pig ACL transection (ACLT) large animal model

**Fig. 6 ∣:**
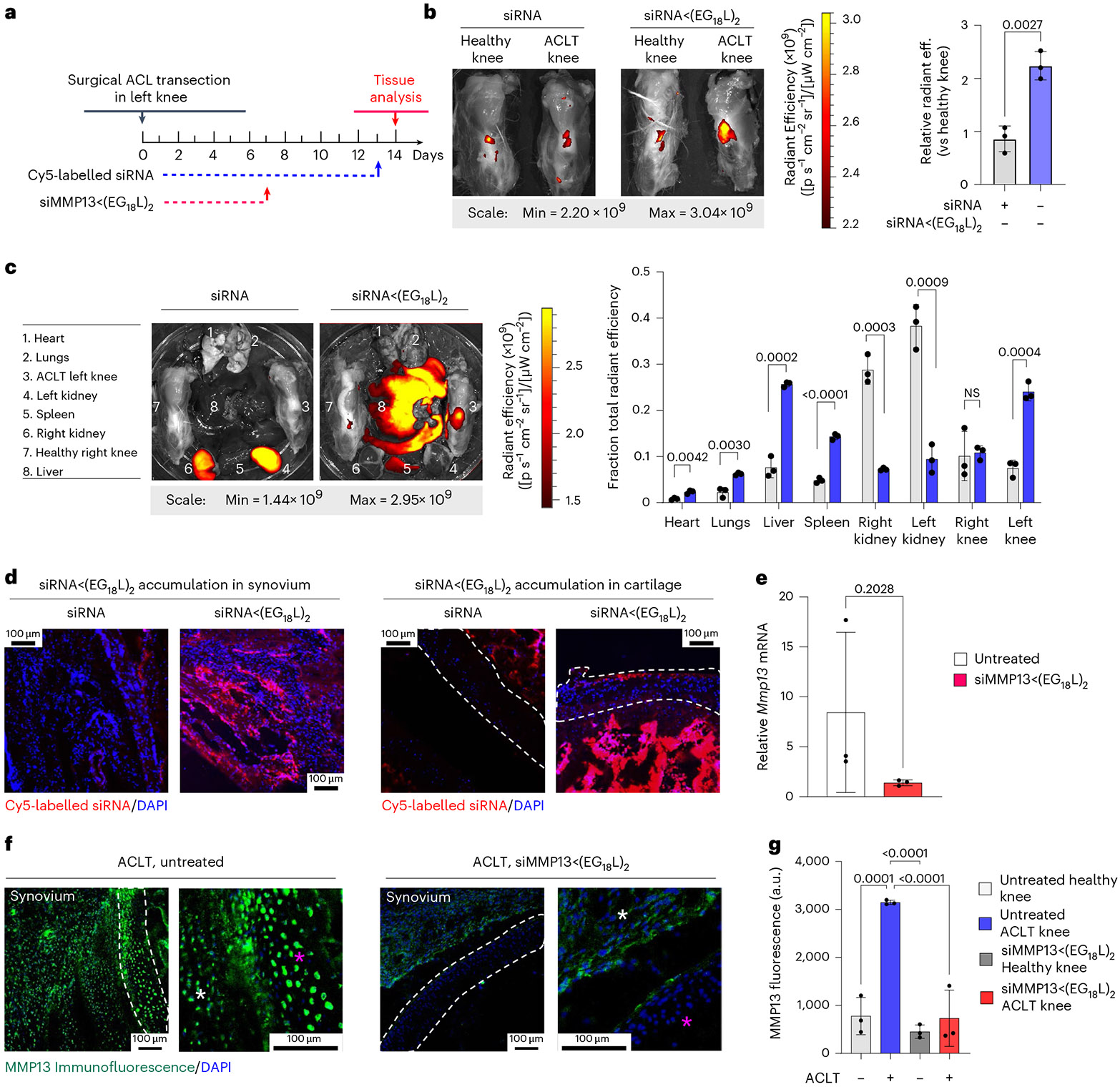
MMP13 silencing by intravenous siMMP13<(EG_18_L)_2_ treatment in guinea pig ACL transection (ACLT) model of arthritis. **a**, Study timeline for ACLT, treatment and sample collection in Dunkin Hartley guinea pigs. **b**–**d**, Cy5 fluorescence was measured ex vivo in healthy and ACLT knees (**b**) and organs (compared using multiple unpaired *t*-tests with no correction for multiple comparisons) (**c**) of guinea pigs by IVIS. Tissues were also assessed by fluorescence microscopy of sagittal ACLT knee cartilage/synovial cryosections (**d**) at 24 h after i.v. delivery of Cy5-siRNA or Cy5-siRNA<(EG_18_L)_2_ at 1 mg kg^−1^. Representative images are shown. In **d**: blue, DAPI; red, Cy5 siRNA. **e**, Guinea pig *Mmp13* mRNA expression (ACLT/healthy knee) as determined by RT–qPCR. **f**,**g**, MMP13 immunofluorescence in cartilage/synovial cryosections of ACLT knee joints that were untreated (left) or treated with siMMP13<(EG_18_L)_2_ (10 mg kg^−1^ i.v.). Asterisks: white, synovium; magenta, femoral condyle cartilage. Representative images are shown with dashed lines outlining articular cartilage (**f**). MMP13 immunofluorescence intensity was quantitated using morphometric software (**g**). All error bars indicate s.d. of biological replicates. Knees labelled healthy indicate a contralateral limb (right) without ACL transection (non-surgical).

ACLT was done on the left knee of Dunkin Hartley guinea pigs (DHGPs) to establish proof of concept in PTOA in a larger species (Fig. 6a). Similar to humans, DHGPs develop OA with aging, with the medial knee compartment showing loss of proteoglycans, fibrillation, cloning of chondrocytes, osteophyte formation and subchondral bone sclerosis^85^. ACLT is used to mimic PTOA and accelerate the spontaneous OA phenotype^86^. MMP13 is strongly expressed and highly localized with OA cartilage lesions in DHGPs^85^. Cy5-siRNA<(EG_18_L)_2_ was delivered i.v. on post-surgical day 13 (1 mg kg^−1^ i.v.), and knees were imaged ex vivo to measure Cy5 fluorescence on day 14, revealing significantly increased Cy5-siRNA<(EG_18_L)_2_ accumulation in ACLT knees over unaltered knees (Fig. 6b), while free Cy5-siRNA did not exhibit preferential accumulation in ACLT-damaged knees. Ex vivo Cy5 imaging of organs showed abundant free siRNA in kidneys, while the albumin-binding siRNA<(EG_18_L)_2_ was found at highest levels in ACLT-damaged knees and liver, but not in healthy knees or in kidneys (Fig. 6c). Fluorescence microcopy confirmed delivery of Cy5-siRNA<(EG_18_L)_2_ but not unconjugated zipper-pattern siRNA to synovia of ACLT-damaged knees, with observable penetration into cartilage (Fig. 6d). Intravenous siMMP13<(EG_18_L)_2_ delivered on post-surgical day 7 at 10 mg kg^−1^ decreased *Mmp13* expression in ACLT-damaged knees by >80% (Fig. 6e) and MMP13 protein expression by >70% on day 14 (Fig. 6f,g).

### Outlook

A unique albumin-binding RNAi conjugate (siRNA<(EG_18_L)_2_) was engineered for MMP13-selective gene targeting in the context of arthritis. This optimized siRNA–lipid conjugate was capable of preferential accumulation and long-term retention within arthritic joints, robustly decreasing arthritis-induced MMP13 and interrupting the forward-feeding cycle of inflammatory joint damage to limit arthritis progression. Overall, this albumin-binding siRNA-L_2_ system shows promise as a systemic and/or local anti-MMP13-specific therapy. This siRNA delivery technology is readily amenable to modular engineering for targeting other arthritis disease-driving genes and/or gene combinations. Beyond the scope of arthritis, we believe this technology may be utilized to deliver to other diseased or injured tissues characterized by inflammation, including tumours, tissues undergoing ischaemic injury and bone fractures.

## Methods

### Materials

Phosphoramidites (2’-O-Me and 2’-F) and universal synthesis columns (MM1-2500-1) were purchased from Bioautomation, symmetrical branching CED phosphoramidite from ChemGenes (CLP-5215), and cyanine 5 phosphoramidite (10-5915), stearyl phosphoramidite (10-1979), hexaethyleneglycol phosphoramidite (10-1918), TEG cholesterol phosphoramidite (10-1976) and desalting columns (60-5010) were from Glen Research. Unless otherwise stated, materials and reagents were purchased from Fisher Scientific or Sigma-Aldrich. CL-82198 was purchased from MedChemExpress (HY-100359). Marimastat was purchased from APExBio (A4049). Clinical formulations of Zilretta and methylprednisolone were purchased through the Vanderbilt University Medical Center pharmacy. C2C ELISA kit and C1,2C antibody were from IBEX Pharmaceuticals.

### siRNA synthesis, modification, annealing and storage

siRNA sequences were synthesized using modified (2’-F and 2’-O-Me) phosphoramidites with standard protecting groups on a MerMade 12 oligonucleotide synthesizer (Bioautomation). Amidites were dissolved at 0.1 M in anhydrous acetonitrile, except for 2’O-Me U-CE phosphoramidite (dissolved in 20% (v/v) anhydrous dimethylformamide) and stearyl phosphoramidite (dissolved in 3:1 (v/v) dichloromethane:acetonitrile). Coupling was performed under standard conditions. Strands were grown on controlled pore glass with a universal terminus (1 μmol scale, 1,000 Å pore), cleaved and deprotected using 1:1 methylamine:40% ammonium hydroxide, except Cy5 sequences which were deprotected in 30% ammonium hydroxide at room temperature for 20 h. Lipophilic RNAs were purified by reverse-phase (RP) high performance liquid chromatography (HPLC) using a Clarity Oligo-RP column (Phenomenex) under a linear gradient from 85% mobile phase A (50 mM triethylammonium acetate in water) to 100% mobile phase B (methanol), or 95% mobile phase A to 100% mobile phase B (acetonitrile). Oligonucleotide-containing fractions were dried, resuspended in nuclease-free water, sterile filtered and lyophilised. Conjugate molecular weight and purity was confirmed by liquid chromatography–mass spectrometry (LC–MS, ThermoFisher LTQ Orbitrap XL Linear Ion Trap mass spectrometer). Chromatography was performed using a Waters XBridge Oligonucleotide BEH C18 column under a linear gradient from 85% A (16.3 mM triethylamine, 400 mM hexafluoroisopropanol) to 100% B (methanol) at 45 °C. Mass spectrometry combined with liquid chromatography was utilized for product characterization (Supplementary Figs. 26-36). Purified oligonucleotide was resuspended in 0.9% sterile saline, annealed to the complementary strand by heating (95 °C) and stepwise cooling (15 °C per 9 min) to 25 °C and then stored at −80 °C.

### MMP13 siRNA delivery to cultured cells

siRNA sequences used are listed in Supplementary Fig. 12. Seven candidate siRNA sequences (Dharmacon) targeting murine *Mmp13* and 4 candidates (Dharmacon) targeting guinea pig *Mmp13* were transfected (Lipofectamine 2000, ThermoFisher) into ATDC5 cells or primary guinea pig chondrocytes (respectively) in OptiMEM for 24 h. Stabilized ‘zipper’ siRNA sequences (50 nM and 100 nM, described below) were transfected into cells using Lipofectamine 2000 in OptiMEM for 24 h. For carrier-free delivery of siRNA and siRNA<(EG_18_L)_2_ to cells, siRNA (1,000 nM OptiMEM) was added to cells for 48 h. In all cases, complete medium supplemented with mouse or guinea pig tumour necrosis factor (TNF) (20 ng ml^−1^) replaced the siRNA containing media for an additional 24 h. RNA was collected, reverse transcribed and assessed by real-time polymerase chain reaction (PCR) using primers against mouse or guinea pig *Mmp13*. CellTiter-Glo (Promega) assay was used to assess cytotoxicity.

### siRNA stability analysis

siRNA (1 nmol) was incubated at 37 °C for 0–24 h in 60% fetal bovine serum (FBS) in PBS, or in patient-derived arthritic joint synovial fluid (83-year-old male patient with untreated, active osteoarthritis). Samples were resolved on 2% agarose gels. Gels were stained with GelRed nucleic acid stain (Biotium) and imaged with UV transillumination.

### siRNA<(EG_18_L)_2_ stability analysis (long-term)

siRNA<(EG_18_L)_2_ (2 nmol) was incubated at 37 °C for 0–96 h in 5 μl patient-derived arthritic joint synovial fluid with rocking. Samples were resolved using 4–20% Native PAGE (Mini-Protean) with 250 ng in each lane. Gels were stained with GelRed nucleic acid stain (Biotium) and imaged with UV transillumination.

### Cell culture

Immortalized mouse chondrogenic ATDC5 cells (ATCC) were cultured in DMEM/F-12, GlutaMAX medium with 10% FBS and 1% penicillin/streptomycin (P/S). Primary guinea pig knee-joint chondrocytes were cultured in DMEM with 10% FBS and 1% P/S. Cells were incubated at 37 °C in 5% CO_2_. All cells tested negative for *Mycoplasma* using MycoAlert Mycoplasma Detection kit (Lonza).

### Quantitation of siRNA uptake in cultured cells

ATDC5 cells (15,000 per well) were seeded overnight in 8-well chamber slides, treated with Cy5-labelled siRNAs pre-complexed with mouse serum albumin (MSA, 10× molar excess) for 4 h in OptiMEM, washed with PBS, fixed and counterstained with DAPI. Cy5 imaging was performed by confocal microscopy on a Nikon Eclipse Ti-0E inverted microscopy base. Alternatively, ATDC5 cells (10,000 per well) in 96-well plates were seeded overnight, treated for 2 h with Cy5-labelled siRNA (100 nM) with or without 1 μM MSA or 10% serum, in OptiMEM. Cells were collected by trypsinization and assessed by flow cytometry on a Guava EasyCyte (Luminex), gating >500 cellular events.

### Size exclusion chromatography (SEC)

Cy5-labelled siRNA molecules (1 μM) were incubated for 30 min with 100 μl patient-derived synovial fluid collected from joints of a non-arthritic donor (myocardial infarction death, no history of rheumatic disease), a donor with untreated osteoarthritis, or a donor with untreated rheumatoid arthritis. The siRNA-synovial fluid mixtures were filtered (0.22 μm) and injected into an AKTA Pure Chromatography System (Cytiva) for fractionation with three inline Superdex 200 Increase columns (10/300 GL) at 0.3 ml min^−1^ using 10 mM Tris-HCl, 0.15 M NaCl and 0.2% NaN_3_. Then, 1.5 ml fractions were collected (F9-C 96-well plate fraction collector, Cytiva). Cy5 fluorescence was measured in fractions (100 μl) in black-walled 96-well plates on a SynergyMx (Biotek). Albumin-associated fractions were determined empirically using known protein standards, examining A280 of eluent from each fraction. Fractions were resolved on 4–20% SDS–PAGE (Mini-Protean), transferred to nitrocellulose (iBlot2, Invitrogen) and stained with Coomassie blue or probed with anti-human albumin antibody (ab19180, Abcam) and IRDye 800CW donkey anti-goat IgG (Li-Cor).

### Mouse models of arthritis

A mechanical overload model of post-traumatic osteoarthritis was used, using an ElectroForce 3100 test frame (TA Instruments). Knee joints of anaesthetized, 6-week-old C57BL/6 male mice were positioned in flexion at 140° with the tibia approximately vertical and placed directly under the loading point^87^. Repetitive knee loading was performed with 250 cycles of 9 N of compressive mechanical force as described in previous studies^62,87^. Loading was repeated three times weekly for up to 5 weeks in either the left knee only, or in both knees, as indicated. For modelling rheumatoid arthritis, serum (200 μl) collected from donor K/BxN transgenic mice was transferred by intraperitoneal delivery to 8-week-old male C57BL/6 mice recipients^76^.

### Cy5 labelling

MSA (Sigma) and 40 kDa PEG (Sigma) were Cy5-labelled (Cy5 conjugation kit, Abcam) and purified, confirming Cy5 fluorescence using a plate fluorimeter (Tecan).

### Pharmacokinetic and retention studies

For initial pharmacokinetic studies, Cy5-siRNA, Cy5-siChol, Cy5-siRNA<(EG_0_L)_2_, Cy5-siRNA<(EG_6_L)_2_, Cy5-siRNA<(EG_18_L)_2_ and Cy5-siRNA<(EG_30_L)_2_ molecules were delivered i.v. (1 mg kg^−1^), subcutaneously (2 mg kg^−1^) or intra-articularly (0.25 mg kg^−1^). Cy5-MSA (3 mg kg^−1^) and Cy5-PEG (3 mg kg^−1^) were delivered i.v. For longer-term retention studies, Cy5-siRNA<(EG_18_L)_2_ was delivered intravenously (10 mg kg^−1^), or by intra-articular injection (1 mg kg^−1^). Mouse hindlimbs were depilated before intravital Cy5 imaging (IVIS Lumina III, Caliper Life Sciences) at indicated time points. Ex vivo Cy5 imaging of organs and denuded limbs collected at necropsy was performed. Where indicated, knee joints were microdissected for Cy5 imaging of knee cartilage and synovial tissues. For Cy5 imaging in histological sections, hindlimbs were embedded into OCT freezing compound, serially sectioned at various depths along the joint at 20 μm, captured onto polyvinylidene chloride film coated with synthetic rubber cement (http://section-lab.jp/), placed on slides, formalin fixed and counterstained with DAPI (in some cases). Cy5 fluorescence was imaged using a Nikon Eclipse Ti inverted confocal microscope. Whole-joint imaging was performed by stitching 4 × 4 images at ×10 magnification.

### Cellular uptake assessment via flow cytometry

Cy5-siRNA<(EG_18_L)_2_ (10 mg kg^−1^) or vehicle (0.9% NaCl) was delivered i.v. to mice that had undergone 1 week of bilateral loading as described above (Supplementary Fig. 11a). Mice were sacrificed 24 h after injection and synovial tissue was isolated from the anterior, medial and lateral compartments as previously described^59^. The posterior synovium was not collected. Two synovia were digested together in a volume of 1.5 ml digestion media (DMEM with 400 μg ml^−1^ collagenase IV, liberase and DNaseI). Synovia were digested for 40 min at 37 °C with intermittent vortexing at 0, 15, 30, 35 and 40 min, and then pelleted at 500 × *g* for 5 min at 4 °C. Each treatment group (vehicle and Cy5-siRNA<(EG_18_L)_2_) comprised 5 male C57/B6 mice, which were pooled and divided into controls and samples. Cold FACS buffer (PBS containing 1% FBS, 2 mM EDTA) was used for all antibody staining and wash steps. Non-specific binding was blocked using Mouse Seroblock FcR (Bio-Rad) for 5 min. CD45-PerCp/Cy5.5 (Biolegend), FAP-AF488 (R&D Systems) and CD31-PE (Invitrogen) were used to identify major synovial cell populations. CD3-SBV515 (Bio-Rad), CD11b-APC/Cy7 (Biolegend), F4/80-PE/Cy7 (Biolegend) and CD11c-BV605 (Biolegend) were used to identify specific immune populations. All cell populations were gated off fluorescence-minus-one (FMO) controls (Supplementary Fig. 11b). The Cy5 gate was established using the vehicle mouse sample and applied to each cell population (Supplementary Fig. 11c), then a %Cy5 positive proportion (Fig. 2f) and median fluorescence intensity (MFI) (Supplementary Fig. 11d) were calculated for all synovial cells and each cell type separately. Experiments included single-stained compensation controls to account for fluorescence bleed over. Acquisition was performed using a BD LSRFortessa cytometer with FACSDiva software (BD Biosciences), then data compensation and analysis was performed using FlowJo v.10 (TreeStar/BD Biosciences). Antibody details are listed in Supplementary Fig. 11e.

### Peptide nucleic acid hybridization assay

Quantification of antisense strands in tissue was performed using a PNA hybridization assay as previously described^88,89^. In brief, knee joints were dissected into mixed cartilage/meniscus and synovial tissues (5–15 mg) and lysed in 300 μl QuantiGene homogenizing solution (Invitrogen) containing 0.2 mg ml^−1^ Proteinase K (Invitrogen). Sodium dodecyl sulfate (SDS) was precipitated from homogenates with 20 μl 3 M potassium chloride and centrifuged at 4,000 × *g* for 15 min. To anneal the PNA probe, 100 μl of hybridization buffer (50 mM Tris, 10% ACN, pH 8.8) and 5 pmol of Cy3-labelled probe (PNABio) were added to 150 μl of each homogenate. Samples were then incubated for 15 min at 90 °C and then at 50 °C before analysis by ion exchange on an iSeries LC equipped with an RF-20A fluorescence detector (Shimadzu) over a DNAPac PA100 anion-exchange column (ThermoFisher). Mobile phases consisted of buffer A (50% acetonitrile and 50% 25 mM Tris-HCl, pH 8.5; 1 mM ethylenediaminetetraacetate in water) and buffer B (800 mM sodium perchlorate in buffer A), and a gradient was obtained as follows: 10% buffer B for 4 min, 50% buffer B for 1 min and 50% to 100% buffer B within 5 min. The final mass of siRNA was calculated from a standard curve of known quantities of siRNA or EG18 spiked into untreated tissue homogenates.

### Therapeutic treatment of arthritis models

Zipper-modified siRNA targeting mouse MMP13 (siMMP13) or non-targeting siRNA (siControl) was delivered to mice at either 5 mg kg^−1^ or 10 mg kg^−1^ i.v., 1 mg kg^−1^ intra-articular, or 20–50 mg kg^−1^ subcutaneous. Mice were treated in parallel studies with Marimastat (10 mg kg^−1^ i.p.), Cl-82198 (10 mg kg^−1^ i.p.), methylprednisolone (10 mg kg^−1^ i.p.) or Zilretta (8 mg kg^−1^)^90^.

### In situ imaging of MMP activity and collagen fragments

Mice were injected with mAbCII-680 (50 μl of 1 mg ml^−1^, tail vein) or with MMPsense 750 fast (100 μl of resuspended formulation following instructions, tail vein, NEV10168, PerkinElmer). Hindlimbs were depilated for IVIS imaging of fluorescence intensity.

### Gene expression analysis in animal tissues

At necropsy, hindlimbs were cleaned of excess muscle and placed in RNAlater solution (ThermoFisher). Anterior synovial tissue pads, menisci, and tibial and femoral articular surface cartilage specimens were collected by microdissection. Cartilage, meniscal tissue and synovial tissue were mixed in equal mass ratios and termed ‘whole joint’ and used for mRNA extraction (RNeasy Plus mini kit from Qiagen). RNA from whole joint, liver or kidney was reverse transcribed (iScript cDNA synthesis kit, Bio-Rad) and used for real-time PCR with the following: murine *Taq*man probes: *Actb* (Mm02619580_ g1), *Gapdh* (Mm99999915_g1), Mmp13 (Mm00439491_m1), *Il1b* (Mm00434228_m1), *Il6* (Mm00446190_m1), *Cox2* (Mm03294838_g1), *Tnf* (Mm00443258_m1); *Sparc*: (Mm00486332_m1), *Cav1* (Mm00483057_m1), *Fcrn* (Mm00438887_m1), *Ngf* (Mm00443039_m1), *Cdkn1b* (Mm00494449_m1); quinea pig *Taq*man probes: *Actb* (Cp03755210_g1), *Gapdh* (Cp03755743_g1), *Mmp13* (APRWJ74). RNA was processed for nanoString analysis using the mouse nCounter Inflammation Panel by the Vanderbilt VANTAGE shared resource, hybridizing for 20 h following manufacturer directions (Nanostring Technologies). Data analysis was performed using nSolver software for comparison, unsupervised analysis and gene cluster analysis between groups. The source data file contains gene names and fold expression changes in knee RNA samples collected from healthy mice, untreated PTOA mice and PTOA mice treated with siMMP13<(EG_18_L)_2_, as well as hindpaw RNA samples collected from healthy mice, untreated K/BxN serum recipient mice and K/BxN serum recipients treated with siMMP13<(EG_18_L)_2_.

### Mining of single-cell RNA-sequencing data for MMP13 expression in synovium

Single-cell RNA-sequencing (scRNAseq) data from ref.^59^ (NIH GEO accession: GSE211584) were employed to identify *Mmp13*-expressing cells in murine PTOA synovium. Data import, quality control, dimensionality reduction and clustering were performed as described in the original publication to yield a cellular atlas of healthy and PTOA synovium comprising lining fibroblasts, sublining fibroblasts, myeloid cells, dendritic cells, T cells, pericytes, endothelial cells, lymphatic endothelial cells, Schwann cells, skeletal muscle and red blood cells. Healthy synovium represented cells from the ‘Sham’ condition (no injury loading, analgesia and anaesthesia only) and PTOA synovium represented merged cells from the ‘ACLR 7 d’ and ‘ACLR 28 d’ conditions, which underwent non-invasive anterior cruciate ligament rupture via mechanical loading. All conditions were made up of pooled male and female mice, with ~20,500 total cells in the analysis. Gene feature plots of normalized *Mmp13* transcript expression were derived to demonstrate the cellular sources and PTOA-associated induction of *Mmp13* in murine synovium.

### Evans blue delivery and extraction

Evans blue (200 μl of 2% w/v in sterile saline) was delivered by intravenous injection. After 24 h, mice were perfused with PBS. Hindlimbs collected at necropsy were air dried overnight. Joint tissues were microdissected. Evans blue was extracted from dissected tissues in formamide at 55 °C for 24 h. Evans blue extracted from tissue was measured as absorbance at 610 nm.

### Immunofluorescence staining

Cryosections (20 μm) were fixed for 10 min with 4% paraformaldehyde, blocked in 5% donkey serum and probed with the following: anti-guinea pig MMP13 (1:100, ARP56350_P050, Aviva Systems Biology); goat anti-rabbit Alexa Fluor 488 (1:500, ab150077, Abcam); *Lycopersicon esculentum* (tomato) lectin (LEL, TL) DyLight 488 (1:100, DL-1174-1). Slides were counterstained with DAPI, mounted with ProLong Gold Antifade and imaged on a Nikon Eclipse Ti inverted confocal microscope. Imaging settings remained constant across all treatment groups.

### Staining of paraffin-embedded histological sections

Joints were formalin fixed, decalcified (20% tetrasodium EDTA) and paraffin embedded. Formalin-fixed paraffin-embedded coronal (knee) or sagittal (paw) sections (5 μm) were stained with haematoxylin and eosin (H&E), toluidine blue, or used for antigen retrieval in Epitope Retrieval 1 (Bond Rx) and immunohistochemistry. The following antibodies were used: anti-MMP13 (1:750, Abcam Ab39012), C1,2C (Col 2 3/4Cshort) polyclonal rabbit antibody (1:500, IBEX Pharmaceuticals, 50-1035). The Bond Refine Polymer detection system was used for visualization. Slides were imaged on the Leica SCN400 slide scanner (Leica Biosystems).

### Clinical and histological scoring of arthritis

Knee-joint hyperalgesia was quantified using a hand-held algesiometer (Bioseb SMALGO, SMall animal ALGOmeter). For measurements, the pressure applicator tip was positioned on the medial aspect of the knee joint and force increased until eliciting a nociceptive response. Each limb was assessed in triplicate. The clinical score of arthritis was assessed as swelling/erythema on a scale of 0–3 (0, none; 1, slight; 2, moderate and in multiple digits; 3, pronounced and in entire paw)^91^. Ankle thickness was measured with calipers. Serum specimens were assessed for collagen degradation fragments using C2C enzyme-linked immunosorbent assay kit (IBEX Pharmaceuticals). Stained coronal knee-joint sections were scored from at least 2 mid-frontal coronal sections per joint^66^ using the OARSI and the DJD scales by a board-certified veterinary pathologist. OARSI scores (0–6 semiquantitative scale; Supplementary Table 1) were assigned from assessment of the medial and lateral plateaus of the tibia and femur^65^. DJD severity score (0–4 semiquantitative scale) was determined from H&E-stained sections using a semiquantitative index (Supplementary Table 1) based on cartilage erosion, subchondral osteosclerosis, synovial/meniscal metaplasia, subchondral osteosclerosis, inflammation, osteophytes and meniscal ectopic mineral deposits^92^. For the K/BxN STA study, hindpaws and forepaws were sectioned sagitally, with knee joints being sectioned coronally; they were also stained with H&E and toluidine blue for scoring purposes. Toluidine blue staining was verified by visualization of mouse skin mast cells and mouse ear cartilage. For the K/BxN serum transfer arthritis model, forepaw, hindpaw and knee-joint sections were also stained with H&E and toluidine blue and were scored using 3 scales adapted as previously described^91,93^ (Supplementary Table 2). Scoring was performed by a histopathologist blinded to the treatment.

### Assessment of siRNA delivery to joint via RNA in situ hybridization

siMMP13<(EG_18_L)_2_ (10 mg kg^−1^) or vehicle (0.9% NaCl) was delivered i.v. to mice that had undergone 1 week of bilateral knee-joint loading as described above. Mice were killed 24 h after injection. Whole knee joints were dissected and fixed in 10% neutral buffered formalin at room temperature for 3 days. Joints were decalcified in 10% tetrasodium EDTA (pH 8) at room temperature under constant agitation for 4 days with a change to fresh EDTA solution after 2 days. Joints were embedded in paraffin, sectioned at 5 μm, mounted on SuperFrost Plus slides (12-550-15, Fisher Scientific) and baked at 60 °C overnight. Slides were deparaffinized through the following solvent series: xylene 10 min, xylene 10 min, 100% ethanol 5 min, 100% ethanol 5 min. Slides were then baked at 60 °C until dry, post fixed in 10% neutral buffered formalin overnight, washed in deionized (DI) water and baked at 60 °C until dry. Tissue sections were incubated with RNAscope hydrogen peroxide (322380, ACD) for 10 min at room temperature, washed in DI water, baked at 60 °C until dry, incubated with custom pretreatment reagent (provided by ACD for bone tissue specimens) for 30 min at 40 °C and washed in DI water. Tissue sections were then incubated as indicated with either positive control probe SR-RNU6-S1 (727871-S1, ACD), negative control probe SR-Scramble-S1 (727881-S1, ACD) or custom miRNAscope Singleplex Target Probe SR-siRNA-Mm-Mmp13 (ACD Sequence 155558, designed against the siMMP13 antisense sequence) for 2 h at 40 °C in a HybEZ II Hybridization System (321710, ACD). Amplification was performed with the miRNAscope HD Detection Kit-RED (324500, ACD) according to manufacturer instructions, with a reduced chromogen development time of 5 min. Slides were counterstained with filtered 50% Gill’s Hematoxylin I (HXGHE1LT, American Master Tech Scientific) for 2 min, washed under running DI water, dipped 5 times in 0.02% ammonia water and baked at 60 °C for 15 min. Slides were briefly dipped in xylene, mounted with Ecomount (EM897L, Biocare Medical) and coverslipped. Whole-slide imaging was performed in the Digital Histology Shared Resource at Vanderbilt University Medical Center, where slides were scanned at ×40 on a Leica SCN400 slide scanner.

### MicroCT

Joints were fixed with formalin and submerged in 100% ethanol during microCT imaging with the ScanCo μCT-50 (Scanco Medical), with reconstructions completed in the ScanCo software (Scanco). Images were collected using 20-μm-thick slices, isotropic 12 μm voxel at 114 mA/70 kVp and 200 ms integration time. Initial contouring encompassed all mineralized joint components. Secondary contouring segmented out mineralization in the soft tissues surrounding cortical bone. Three-dimensional (3D) renderings (sigma: 1.5; support: 3; threshold: 388) were generated using Scanco software and are shown at a consistent density threshold (42.0% of maximum bone density, or 420 per mille). Imaging, contouring and sample measurements were acquired by a treatment-blinded user. Osteophyte length was measured on each sample in femur and tibia. For K/BxN serum recipients, contouring was performed over the calcaneus. Hydroxyapatite calibration phantoms were used to calibrate bone density values (g cm^−3^).

### Guinea pig anterior cruciate ligament model

Dunkin Hartley guinea pigs (3-month-old male) (Charles River Laboratories) underwent left-knee ACLT surgery using procedures adapted from ref. 94. Surgery was performed under direct visualization in anaesthetized animals. A medial longitudinal parapatellar incision over the anterior knee exposed the patellar tendon. The patella was everted, the knee placed in flexion and the ACL incised. After confirming anterior joint laxity, the site was closed with the joint capsule continuously sutured. Ketoprofen was given immediately before and every 24 h post surgery for 3 days.

### Blood chemistry

Whole blood was collected in EDTA-coated tubes or spun down at 1,000 × g for 10 min at 4 °C to isolate serum. Samples were then submitted to the Vanderbilt Translational Pathology Shared Resource for chemistry analyses.

### Animal ethics statement

All animal experiments described herein were carried out according to protocols approved by Vanderbilt University’s Institutional Animal Care and Use Committee, and all studies followed the National Institutes of Health’s guidelines for the care and use of laboratory animals.

### Statistical methods

Data are displayed as mean plus/minus standard deviation (unless stated otherwise). Statistical tests employed either one-way or two-way analysis of variance (ANOVA) with multiple comparisons test or two-tailed Student’s *t*-test between only two groups with *α* = 0.05 unless otherwise indicated. All statistical analyses were performed as described in figure captions using GraphPad Prism software, except for nanoString data analysis with nSolver v.3.0 (Nanostring Technologies).

## Extended Data

## Supplementary Material

si doc

si tables

**Supplementary information** The online version contains supplementary material available at https://doi.org/10.1038/s41551-025-01376-x.

## Data Availability

The main data supporting the results in this study are available within the paper and its Supplementary Information. Raw and normalized nanoString datasets are available at the Gene Expression Omnibus under accession identifier GSE233714. The remaining raw and analysed datasets from the study are available for research purposes from the corresponding author on reasonable request. Source data are provided with this paper.
