# Supplementary material for: siRNA conjugate with high albumin affinity and degradation resistance for delivery and treatment of arthritis in mice and guinea pigs
Source: Nat Biomed Eng. 2025 May 16;9(8):1366–83. doi: 10.1038/s41551-025-01376-x (PMC12354308; doi:10.1038/s41551-025-01376-x)
Supplement: Supplementary file 1 — Supplementary Figs. 1–36. [file 41551_2025_1376_MOESM1_ESM.pdf]

nature biomedical engineering

Supplementary information https://doi.org/10.1038/s41551-025-01376-x

siRNA conjugate with high albumin affinity and degradation resistance for delivery and treatment of arthritis in mice and guinea pigs

In the format provided by the authors and unedited

Table of Contents

Supplementary Fig. 1 | Albumin accumulates in cartilage and synovial tissues of mechanically loaded OA knees to a greater extent than Poly(ethylene glycol).

Supplementary Fig. 2 | Representative samples and processing protocol with standard curve and equation for Evans Blue ng/mg tissue quantification.

Supplementary Fig. 3 | Characterization of albumin delivery/transport in the mechanical loading PTOA model.

Supplementary Fig. 4 | Biodistribution of systemically delivered lipophilic Cy5-si<(EG$_X$L)$_2$ series in mechanically loaded animals (24 hours after 1 mg/kg i.v. injection).

Supplementary Fig. 5 | Albumin-hitchhiking siRNA<(EG$_{18}$L)$_2$ reaches cartilage and synovial tissues within loaded, arthritic knee joints (24 hours after 1 mg/kg i.v. injection).

Supplementary Fig. 6 | Albumin-hitchhiking siRNA<(EG$_{18}$L)$_2$ reaches cartilage, meniscal, and synovial tissues within loaded, arthritic knee joints (24 hours after 10 mg/kg i.v. injection).

Supplementary Fig. 7 | Albumin-hitchhiking siRNA<(EG$_{18}$L)$_2$ accumulate within loaded, arthritic knee joint tissues (24 hours after 1 mg/kg i.v. and i.a. injection).

Supplementary Fig. 8 | Albumin-hitchhiking siRNA<(EG$_{18}$L)$_2$ demonstrates preferential delivery to loaded, arthritic knee joints (24 hours after 1 mg/kg i.v. injection).

Supplementary Fig. 9 | siRNA<(EG$_{18}$L)$_2$, but not free siRNA, binds to albumin in human synovial fluid, with increased binding in specimens from OA- and RA-affected joints.

Supplementary Fig. 10 | In situ hybridization control experiment in PTOA knee joints with siMMP13<(EG$_{18}$L)$_2$.

Supplementary Fig. 11 | siRNA<(EG$_{18}$L)$_2$ exhibits high levels of cellular uptake across several cell types (24 hours after 1 mg/kg i.v. injection).

Supplementary Fig. 12 | Mouse and guinea pig siRNA sequences and modifications used herein.

Supplementary Fig. 13 | MMP13 siRNA sequence screening across species, carrier free activity, and stability.

Supplementary Fig. 14 | Carrier-Free Mediated Cell Uptake in Chondrogenic Murine ATDC5 Cells.

Supplementary Fig. 15 | Mmp13 is upregulated in synovial fibroblasts and myeloid cells.

Supplementary Fig. 16 | Pharmacokinetic and biodistribution analyses of intra-venous vs. intra-articular route.

Supplementary Fig. 17 | Biodistribution and pharmacokinetics of K/BxN STA model.

Supplementary Fig. 18 | Molecular analysis of K/BxN therapeutic study (10 mg/kg).

Supplementary Fig. 19 | Further characterization of K/BxN therapeutic study (10 mg/kg).

Supplementary Fig. 20 | Representative lower magnification knee joint histology for PTOA and K/BxN therapeutic studies.

Supplementary Fig. 21 | Toluidine Blue of forepaw / wrist cartilage of K/BxN serum recipients.

Supplementary Fig. 22 | H&E of forepaw/wrist tissue for the K/BxN therapeutic study.

Supplementary Fig. 23 | Bone loss and ankle joint assessments in K/BxN therapeutic study.

Supplementary Fig. 24 | Systemic toxicity analyses in K/BxN and PTOA model therapeutic studies.

Supplementary Fig. 25 | H&E-stained sections of organs (liver, kidney, lung, heart, and spleen) from mice enrolled in the different treatment groups in the PTOA and K/BxN therapeutic studies.

Supplementary Fig. 26 | LC-MS characterization of control sense siRNA sequence.

Supplementary Fig. 27 | LC-MS characterization of control sense – cholesterol modifier siRNA sequence.

Supplementary Fig. 28 | LC-MS characterization of control sense – <(EG$_{18}$L)$_2$ modifier siRNA sequence. Supplementary Fig. 29 | LC-MS characterization of control antisense – siRNA sequence.

Supplementary Fig. 30 | LC-MS characterization of control antisense – Cy5 modifier siRNA sequence. Supplementary Fig. 31 | LC-MS characterization of mouse MMP13 antisense – siRNA sequence.

Supplementary Fig. 32 | LC-MS characterization of mouse MMP13 sense – siRNA sequence.

Supplementary Fig. 33 | LC-MS characterization of mouse MMP13 sense – <(EG$_{18}$L)$_2$ modifier siRNA sequence.

Supplementary Fig. 34 | LC-MS characterization of guinea pig MMP13 sense siRNA sequence.

Supplementary Fig. 35 | LC-MS characterization of guinea pig MMP13 sense – <(EG$_{18}$L)$_2$ modifier siRNA sequence.

Supplementary Fig. 36 | LC-MS Characterization of Guinea Pig MMP13 Antisense – siRNA Sequence.

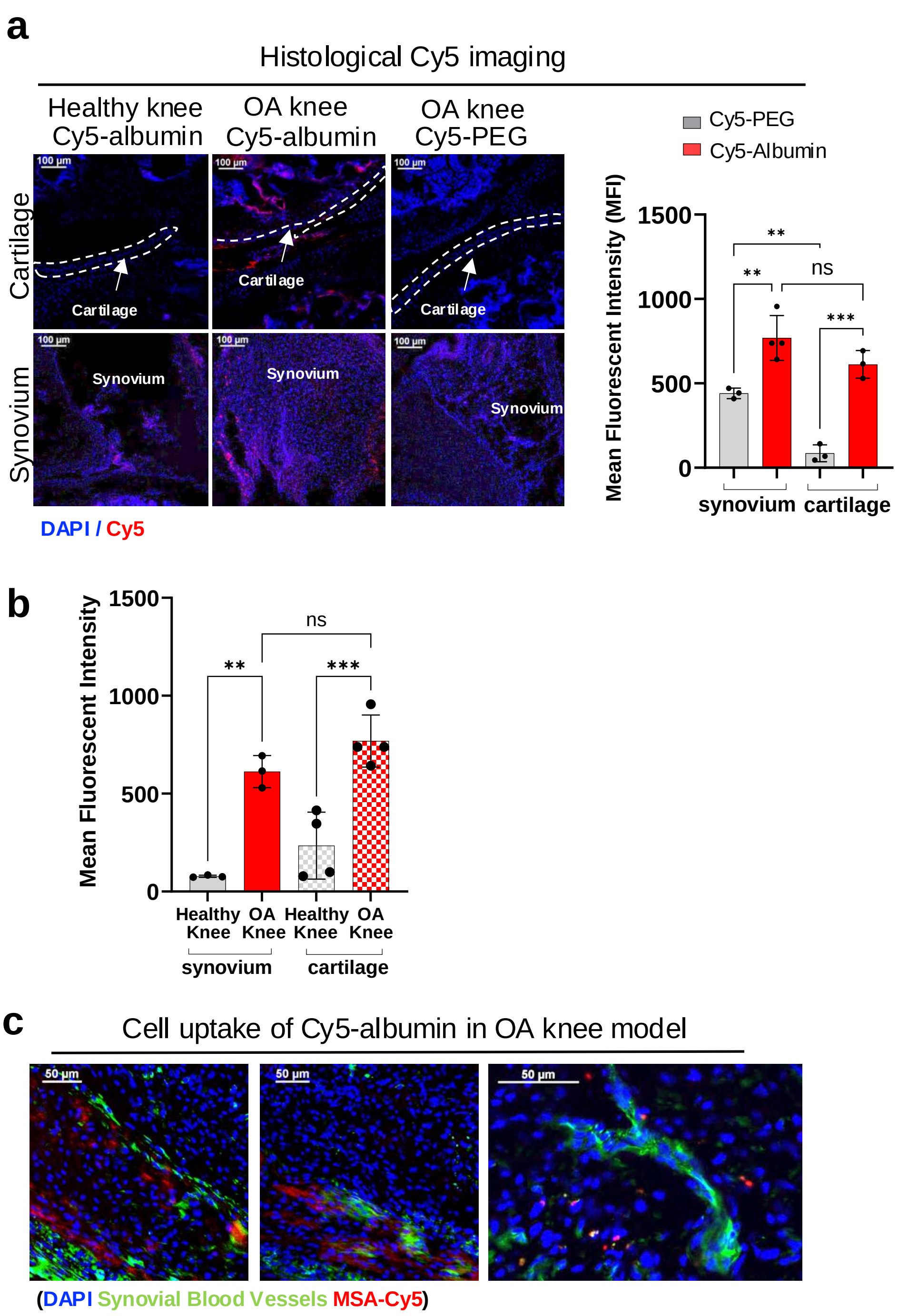

Supplementary Figure 1. Albumin accumulates in cartilage and synovial tissues of mechanically loaded OA knees to a greater extent than Poly(ethylene glycol).

A) Representative sagittal cryosections (20X) of knee joints used for assessing MSA-Cy5 (N=5) and PEG-Cy5 (N=3). Fluorescent signal within cartilage and synovial compartments of PTOA mouse knee joints.

B) Fluorescent signal within cartilage and synovial compartments of PTOA and healthy mouse knee joints treated with MSA-Cy5. Healthy indicates a non-loaded contralateral knee.

C) Representative cryosections (20X) of knees assessing MSA-Cy5 and synovial blood vessels by lectin IHC.

Representative Statistics markers: *P < 0.05, **P < 0.01, ***P < 0.001, ****P < 0.0001.

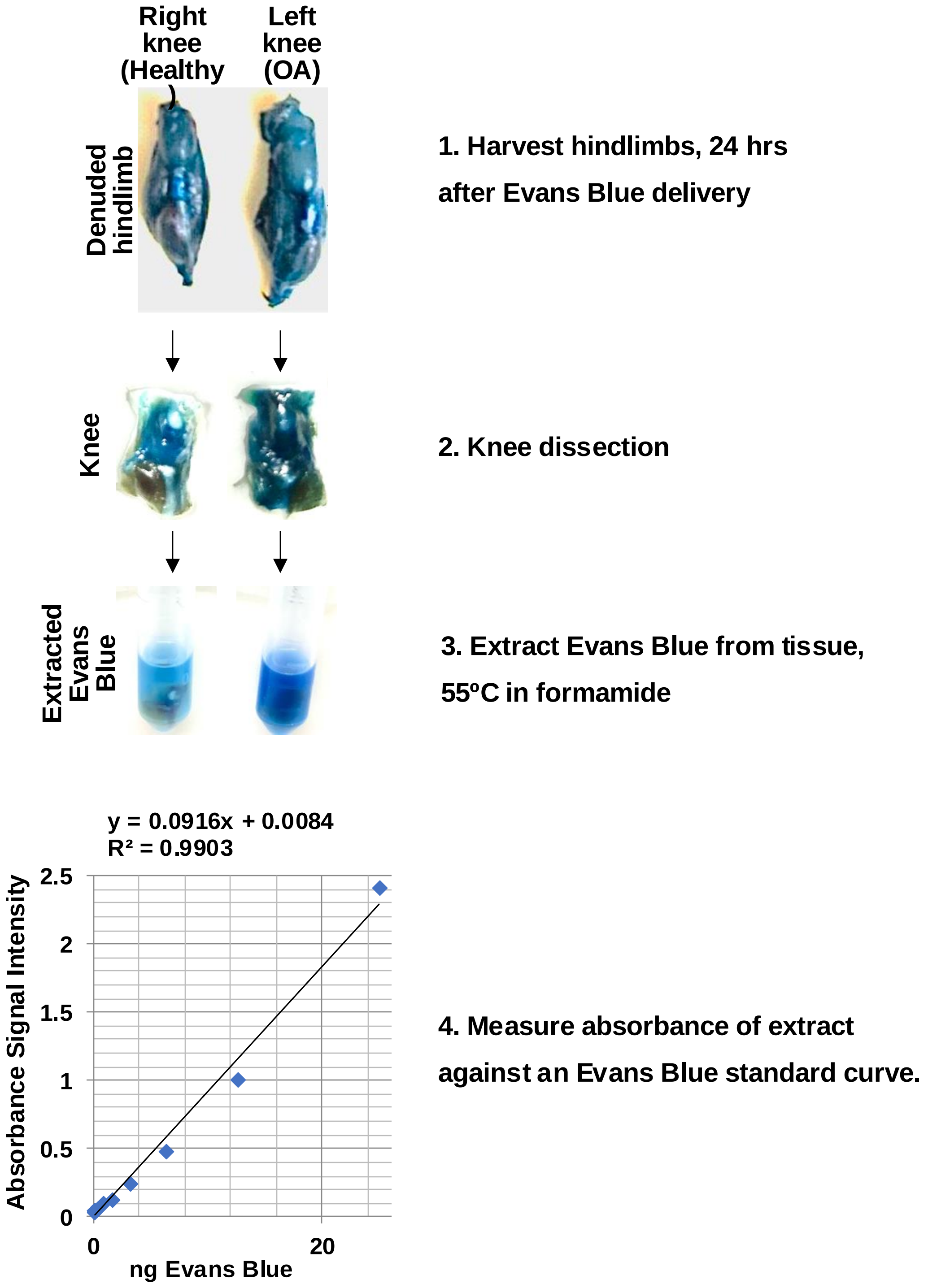

Supplementary Figure 2. Representative samples and processing protocol with standard curve and equation for Evans Blue ng/mg tissue quantification.

Knees labeled healthy indicate a non-loaded contralateral limb (right).

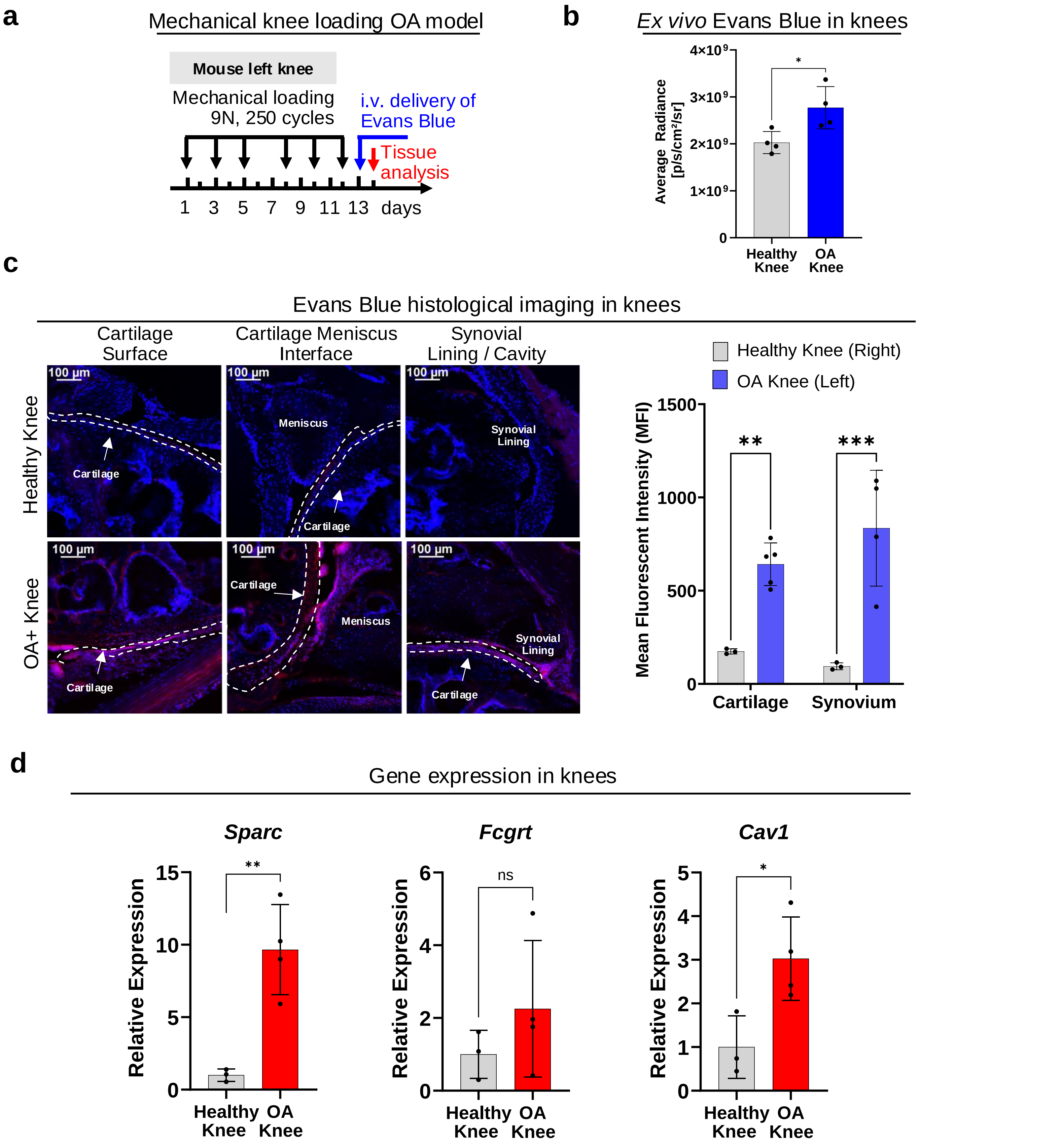

Supplementary Figure 3. Characterization of albumin delivery/transport in the mechanical loading PTOA model.

A) Unilateral (left knee loaded, right knee unloaded) mechanical loading protocol/timeline used and injection protocol for Evans Blue dye (N=4).

B) Intravital (IVIS) quantification of Evans Blue dye.

C) Representative cryohistology images (20X) of sagittal-sectioned knee joints 24 hours after intravenous Evans Blue injection (Blue = DAPI, Red = Evans Blue). On right is quantification of Evans Blue cryohistologyimages with a specific focus on cartilage and synovial tissue (N=3-6).

D) qRT-PCR of cartilage/synovial tissue mix for SPARC, FcRn, Caveolin-1 (N=3-4).

Statistics markers: *P < 0.05, **P < 0.01, ***P < 0.001, ****P < 0.0001. Healthy indicates a non-loaded contralateral knee.

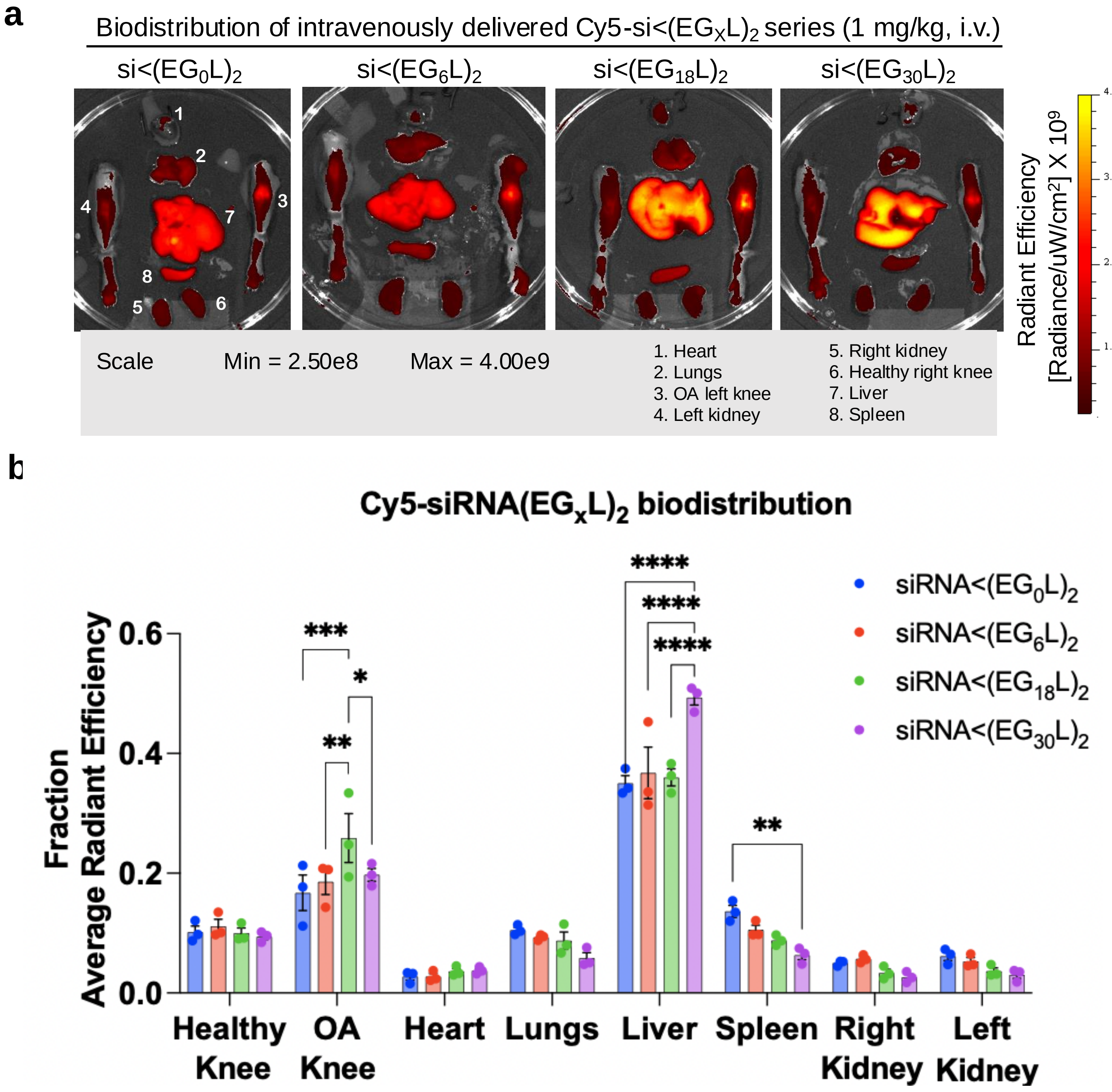

Supplementary Figure 4. Biodistribution of systemically delivered lipophilic Cy5-si<(EG$_X$L)$_2$ series in mechanically loaded animals (24 hours after 1 mg/kg i.v. injection).

A) Representative ex-vivo IVIS images for systemic i.v. Cy5-conjugated siRNA molecules.

B) Organ biodistribution quantification (N=3). Healthy indicates a non-loaded contralateral knee. Statistics markers: *P < 0.05, **P < 0.01, ***P < 0.001, ****P < 0.0001.

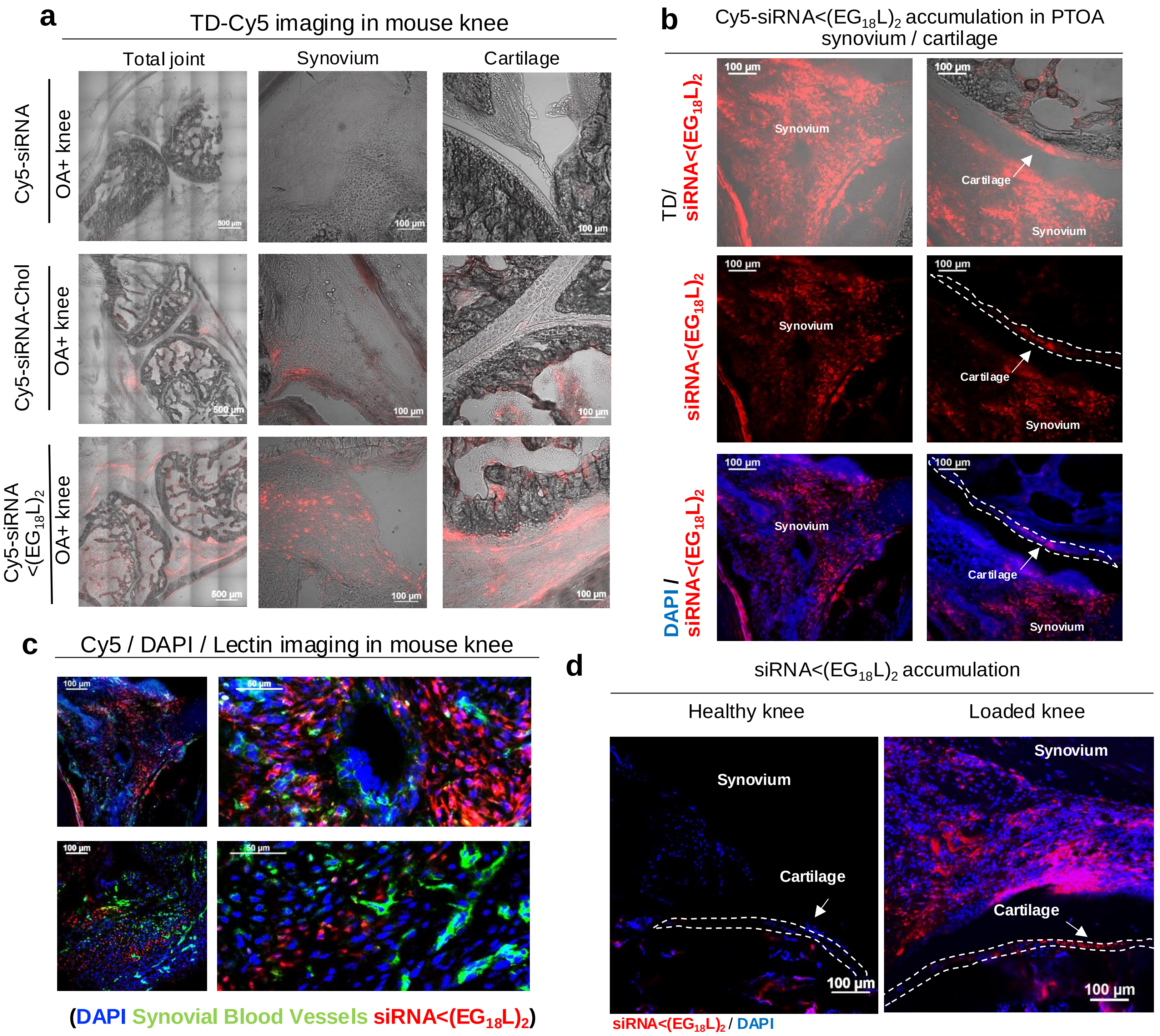

Supplementary Figure 5. Albumin-hitchhiking siRNA<$(EG_{18}L)_2$ reaches cartilage and synovial tissues within loaded, arthritic knee joints (24 hours after 1 mg/kg i.v. injection).

A) Confocal microscopy of knee joint tissues of mice injected with Cy5-conjugated siRNA molecules – siRNA, siChol, and siRNA<$(EG_{18}L)_2$.

B) Additional microscope images of Cy5-siRNA<$(EG_{18}L)_2$ (red) molecules in synovial and cartilage tissues in loaded, arthritic knee joints.

C) Confocal microscopy of Cy5-siRNA<$(EG_{18}L)_2$ (red) in loaded knees stained for blood vessels (indicated by Lectin IHC) in the synovium/extra - articular tissues (green).

D) Microscope images of Cy5-siRNA<$(EG_{18}L)_2$ (red) molecules in synovial and cartilage tissues in loaded, arthritic knee joints vs. healthy, unloaded knee joints.

TD – Transmission Detector. Healthy indicates a non-loaded contralateral knee.

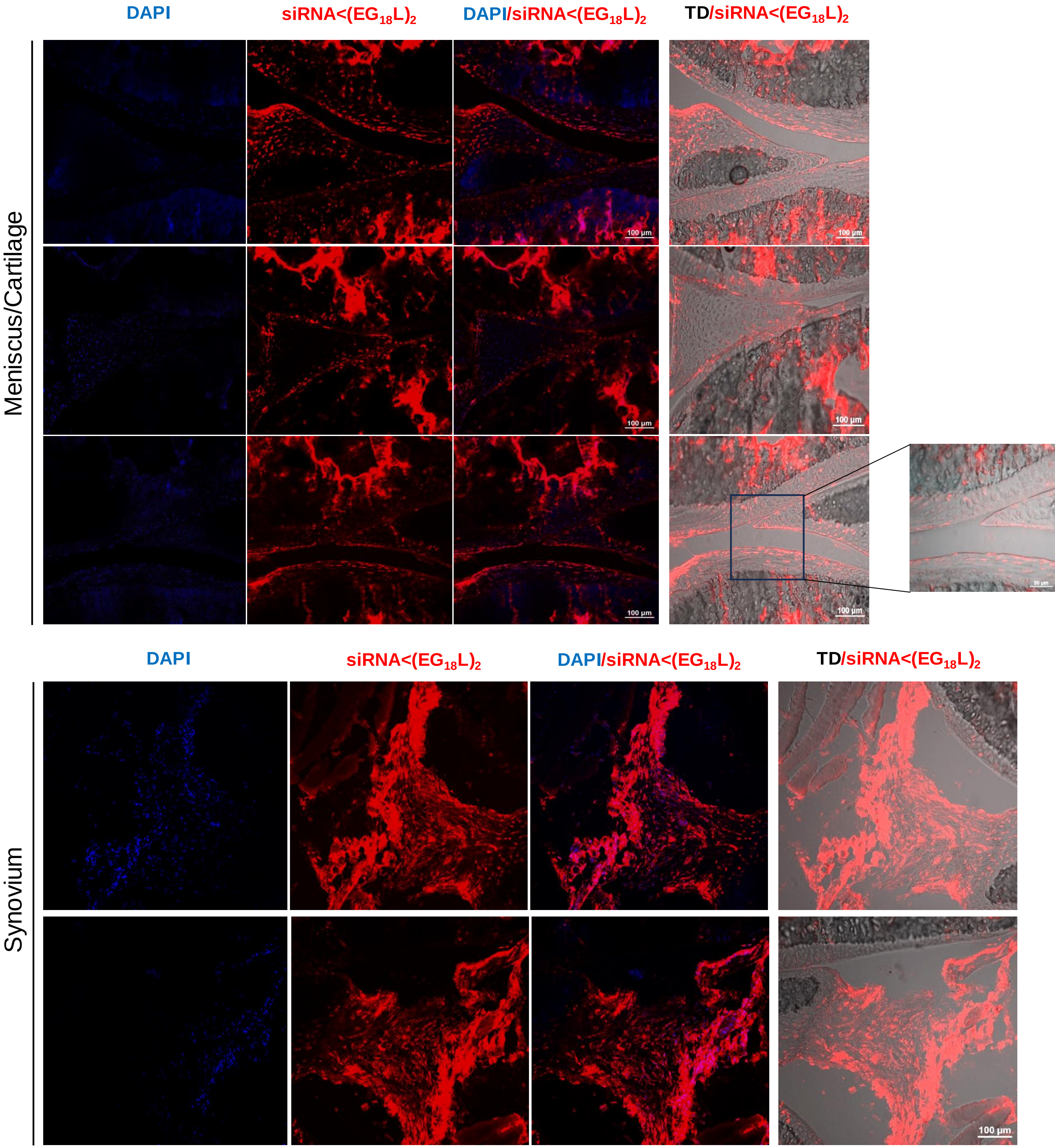

Supplementary Figure 6. Albumin-hitchhiking siRNA<(EG$_{18}$L)$_2$ reaches cartilage, meniscal, and synovial tissues within loaded, arthritic knee joints (24 hours after 10 mg/kg i.v. injection).

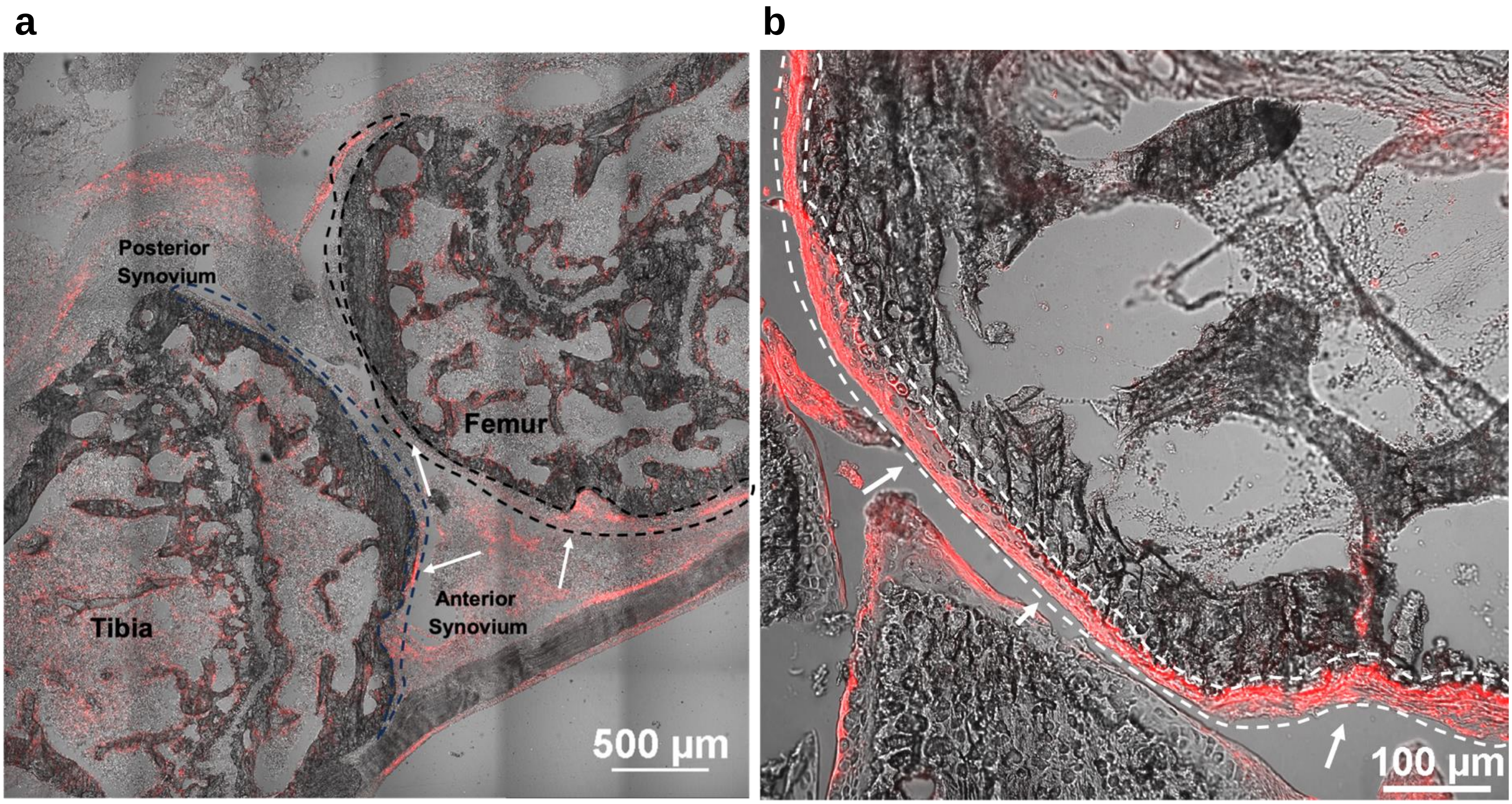

TD/siRNA<(EG$_{18}$L)$_2$

Supplementary Figure 7. Albumin-hitchhiking siRNA<(EG$_{18}$L)$_2$ accumulate within loaded, arthritic knee joint tissues (24 hours after 1 mg/kg i.v. and i.a. injection).

A) Cryosectioned whole knee joint collected 24 hours after i.v. delivery of 1 mg/kg Cy5-siRNA<(EG$_{18}$L)$_2$, the albumin-binding siRNA-lipid conjugate. Cartilage is outlined and arrows indicate Cy5 signal within the cartilage.

B) Cryosectioned knee joint collected 24 hours after i.a. delivery of 1 mg/kg Cy5-siRNA<(EG$_{18}$L)$_2$, the albumin-binding siRNA-lipid conjugate. Cartilage is outlined and arrows indicate Cy5 signal within the cartilage.

TD – Transmission Detector.

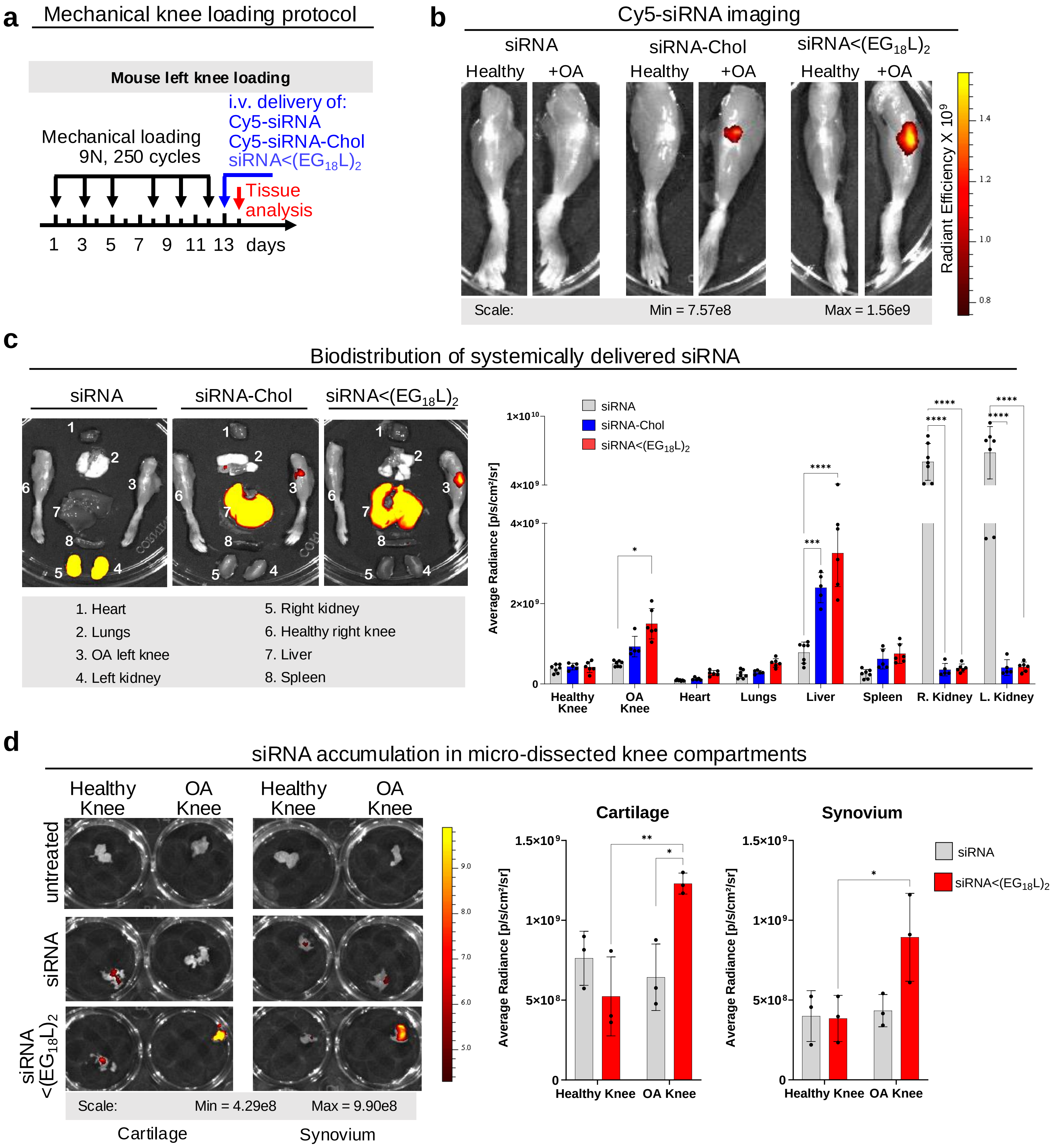

Supplementary Figure 8. Albumin-hitchhiking siRNA<($EG_{18}L)_2$ demonstrates preferential delivery to loaded, arthritic knee joints (24 hours after 1 mg/kg i.v. injection).

A) Unilateral mechanical knee loading protocol/timeline used and injection schedule for systemic delivery of Cy5-conjugated siRNA molecules.

B) Representative ex-vivo IVIS images.

C) Organ biodistribution representative images and quantification (N=5-7).

D) Representative IVIS images of cartilage and synovial tissues dissected out of arthritic and healthy knee joints and quantification of IVIS signal (N=3).

Statistics markers: $^*P < 0.05$, $^{**}P < 0.01$, $^{***}P < 0.001$, $^{****}P < 0.0001$. Healthy indicates a non-loaded contralateral knee.

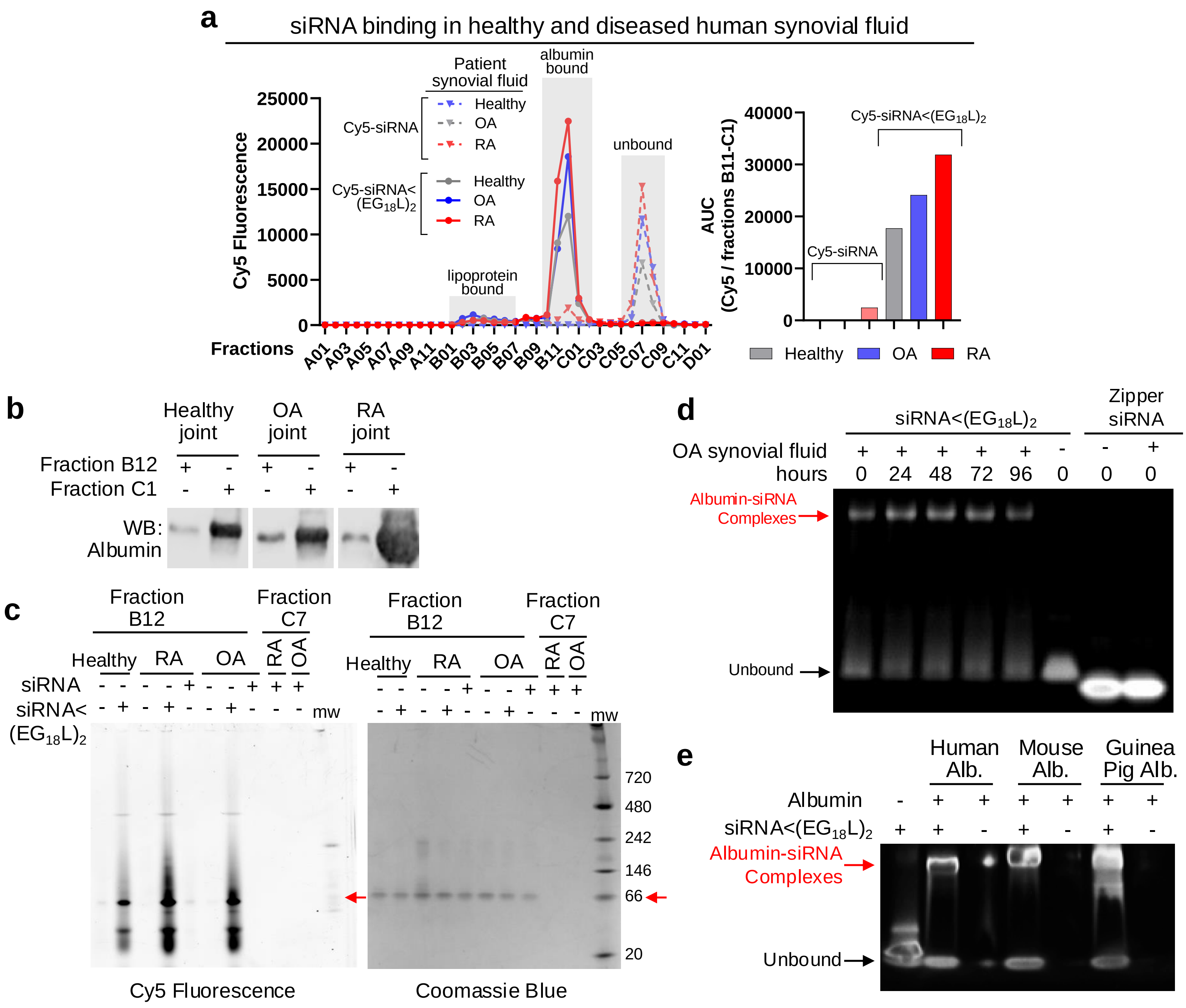

Supplementary Figure 9. siRNA<($EG_{18}L$)$_2$, but not free siRNA, binds to albumin in human synovial fluid, with increased binding in specimens from OA- and RA-affected joints.

A) Chromatographic assessment of binding of Cy5-siRNA and Cy5-siRNA<($EG_{18}L$)$_2$ to different components of human synovial fluid taken from a healthy, osteoarthritis, and rheumatoid arthritis patients. Cy5 fluorescence within the albumin-containing fractions was quantitated as area under the curve (AUC).

B) Western blot for human albumin in B12 and C1 synovial fluid fractions shows increased albumin in OA- and RA-derived specimens.

C) Fractions B12 and C7 were fractionated by SDS-PAGE, assessing both Cy5 fluorescence (left panel) and total proteins (coomassie blue). Fraction C7 was used as a negative control, non-albumin containing fraction where unbound siRNA elutes. Red arrow indicates molecular weight of human albumin (66 kDa).

D) Native PAGE of siRNA<($EG_{18}L$)$_2$ sequences after incubation in synovial fluid collected from an OA patient compared to siRNA<($EG_{18}L$)$_2$ in water and zipper siRNA.

E) Gel electrophoresis mobility shift assay demonstrating tendency of siRNA<($EG_{18}L$)$_2$ to bind/interact with albumin in a species-independent manner.

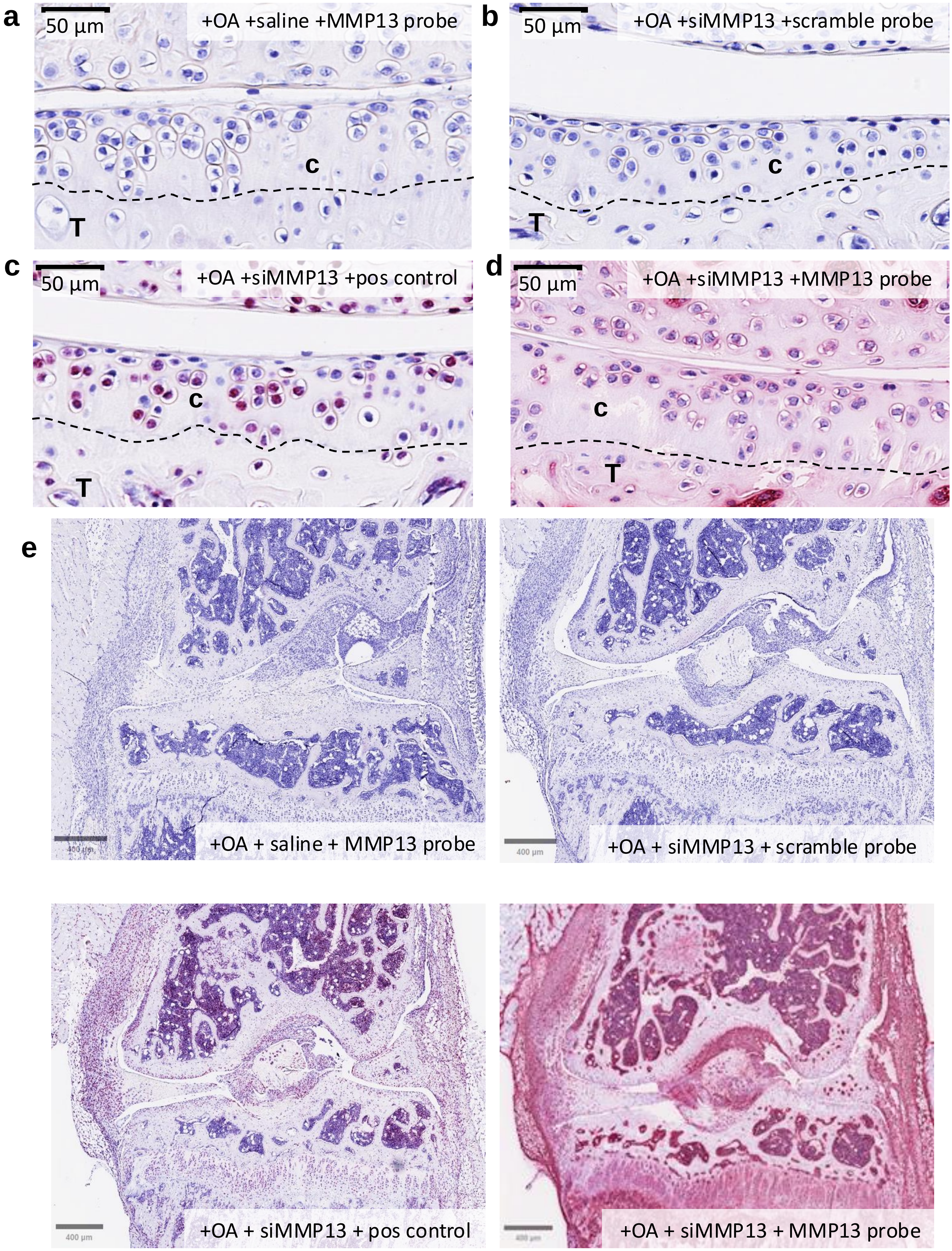

Supplementary Figure 10. In situ hybridization control experiment in PTOA knee joints with siMMP13<($EG_{18}L)_2$.

A) Saline treatment + MMP13 probe showing no staining.

B) siMMP13<($EG_{18}L)_2$ treatment + scramble probe showing no staining.

C) siMMP13<($EG_{18}L)_2$ treatment + positive control probe (RNU6) showing punctate nuclear staining.

D) siMMP13<($EG_{18}L)_2$ treatment + MMP13 probe showing diffuse signal with punctate staining around chondrocytes.

E) Whole joint specimens.

*C = cartilage, T = tibia.

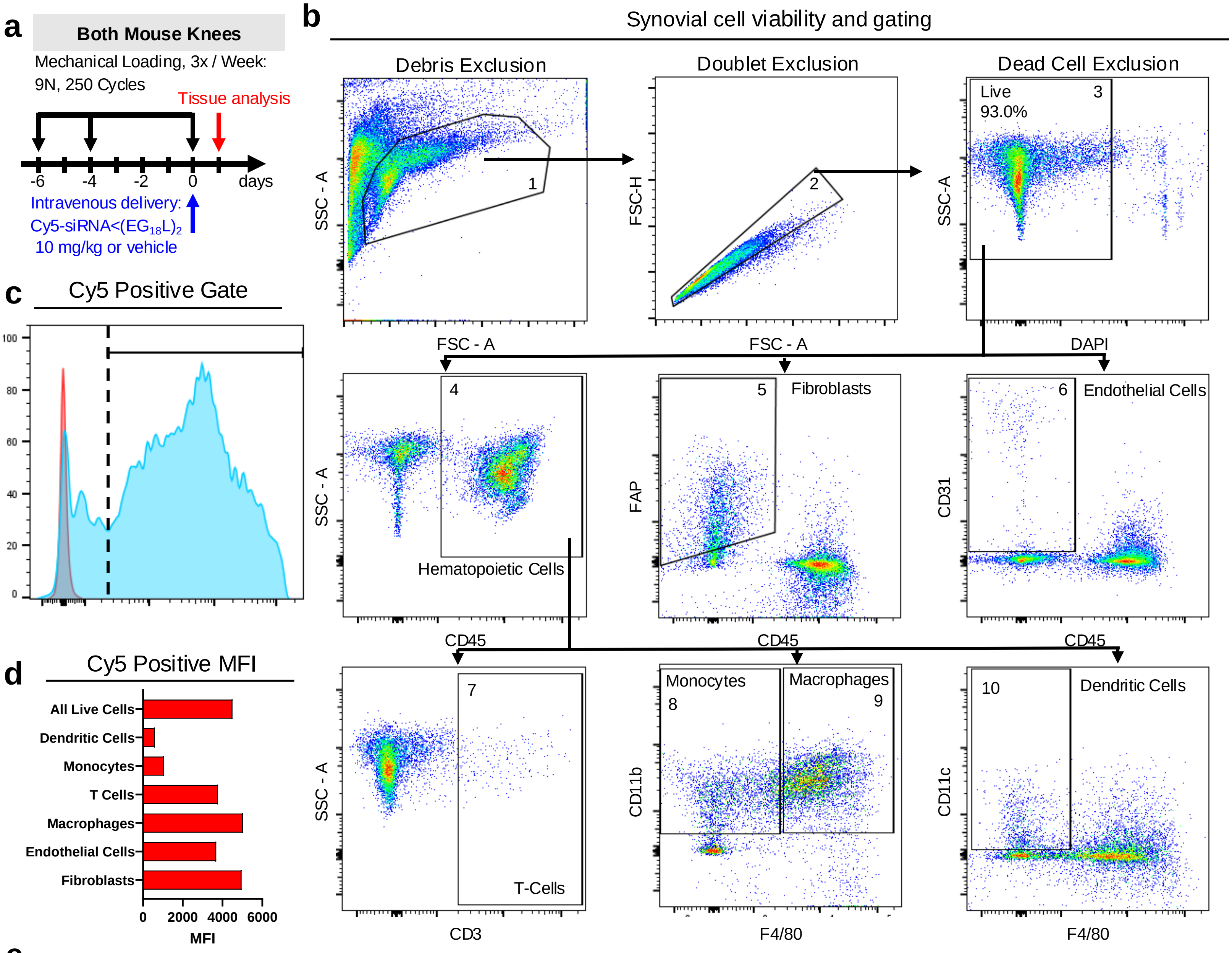

e

Antigen	Clone	Fluorophore	Cat#
Viability	-	DAPI	
CD45	QA17A26	PerCP-Cy5.5	Biolegend 157611
FAP	983802	AF488	R&D Systems FAB9727G
CD31	390	PE	Invitrogen 12-0311-82
CD3	KT3	Starbright Violet 515	Bio-Rad MCA500SBV515
CD11b	M1/70	APC-Cy7	Biolegend 101226
CD11c	N418	BV605	Biolegend 117333
F4/80	BM8	PE-Cy7	Biolegend 123114

Supplementary Figure 11. siRNA<(EG$_{18}$L)$_2$ exhibits high levels of cellular uptake across several cell types (24 hours after 10 mg/kg i.v. injection).

A) Bilateral mechanical knee loading protocol/timeline used and injection schedule for systemic delivery of Cy5-siRNA<(EG$_{18}$L)$_2$.

B) Flow cytometry gating strategies of synovial cells to assess viability and separate major cell populations. Cells were gated on SSC-A and FSC-A for small debris exclusion (1), then single cells gated on FSC-A and FSC-H (2). Dead cell exclusion (3, including percentage of live cells) was performed, and the resulting live single cells were analyzed for cell type. Cells were first gated into hematopoietic cells (4, CD45+), fibroblasts (5, FAP+, CD45-) and endothelial cells (6, CD31+, CD45-). The hematopoietic population (4) was then refined to T-cells (7, CD3+, CD45+), monocytes (8, CD11b+, F4/80-, CD45+), macrophages (9, CD11b+, F4/80+, CD45+) and dendritic cells (10, CD11c+, F4/80-, CD45+).

C) All single live cells gated on Cy5, indicating siRNA<(EG$_{18}$L)$_2$ uptake.

D) Mean fluorescence intensity (MFI) of Cy5 positive cells.

E) Table of antibody clones and fluorophores used in flow cytometry experiment.

Mouse Sequence Name	Antisense Strand	Sense Strand
siMMP13.1 (Lead Sequence Candidate)	5'UUUCUCAUGAUGUCUAAGGUU3'	5'CCUUAGACAUCAUGAGAAAUU3'
siMMP13.1 (with 19-mer Zipper Chemisty) PHO: Phosphate, *: Phosphorothioate, f: 2'fluoro, Me: 2'O-methyl	5'(PHO)(MeU)*(fU)*(MeU) (fC)(MeU)(fC) (MeA)(fU)(MeG) (fA)(MeU)(fG) (MeU)(fC)(MeU) (fA)(MeA)*(fG)*(MeG)3' MW = 6303 g/mole	5' (fC)*(MeC)*(fU) (MeU)(fA)(MeG) (fA)(MeC)(fA) (MeU)(fC)(MeA) (fU)(MeG)(fA) (MeG)(fA)*(MeA)*(fA) 3' MW = 6263.1 g/mole
siMMP13.2	5'UCACUGUAGACUUCUUCAGUU3'	5'CUGAAGAAGUCUACAGUGAUU3'
siMMP13.3	5'UUUCUCGGAGUCUAUCAACUU3'	5'GUUGAUAGACUCCGAGAAAUU3'
siMMP13.4	5'AAACUCAAGAUCUUCCUCAUU3'	5'UGAGGAAGAUCUUGAGUUUUU3'
siMMP13.5	5'UUUCUCGGAGUCUAUCAACUU3'	5'GUUGAUAGACUCCGAGAAAUU3'
siMMP13.6	5'AAACUCAAGAUCUUCCUCAUU3'	5'UGAGGAAGAUCUUGAGUUUUU3'
siMMP13.7	5'UAAAGAUCAUGGUUUCUCCUU3'	5'GGAGAAACCAUGAUCUUUAUU3'
siControl (Targeting firefly luciferase)	5'UUCAUUAUCAGUGCAAUUG3'	5'CAAUUGCACUGAUAAUGAA3'
siLUC used as a siNEG sequence → Targets Firefly Luciferase (Not in the mouse genome) *For Fluorescent Cy5 Version, switch 5'PHO for 5'Cy5	5'(PHO)(MeU)*(fU)*(MeC)(fA)(MeU)(fU) (MeA)(fU)(MeC)(fA)(MeG)(fU)(MeG)(fC) (MeA)(fA)(MeU)*(fU)*(MeG)3' MW = 6287 g/mole	5'(fC)*(MeA)*(fA)(MeU)(fU)(MeG)(fC) (MeA)(fC)(MeU)(fG)(MeA)(fU)(MeA) (fA)(MeU)(fG)*(MeA)*(fA)3' MW = 6264.1 g/mole

Guinea Pig Sequence Name	Antisense Strand	Sense Strand
siMMP13.1	5'UAUCUGUCCAGUAUUCAUCUU3'	5'GAUGAAUACUGGACAGAUAUU3'
siMMP13.2 (Lead Sequence Candidate)	5'UUAAUGUCAUCAUAUCUCCUU3'	5'GGAGAUAUGAUGACAUUAAUU3'
siMMP13.2 (19-mer Zipper Chemistry used for Therapeutic Studies) PHO = Phosphate, *= Phosphorothioate, f = 2'fluoro, Me = 2'O-methyl	5'(PHO)(MeU)*(fU)*(MeA)(fA)(MeU)(fG)(MeU)(fC)(MeA)(fU)(MeC)(fA)(MeU)(fA)(MeU)(fC)(MeU)*(fC)*(MeC)3' MW = 6206.9 g/mole	5'(fG)*(MeG)*(fA)(MeG)(fA)(MeU)(fA)(MeU)(fG)(MeA)(fU)(MeG)(fA)(MeC)(fA)(MeU)(fU)*(MeA)*(fA)3' MW = 6344.2 g/mole
siMMP13.3	5'AUGCCCGCAAGAUUUACUGUU3'	5'CAGUAAAUCUUGCGGCAUUU3'
siMMP13.4	5'UUGCCUGUCACCUCUAAGCUU3'	5'GCUUAGAGGUGACAGGCAAUU3'

Supplementary Figure 12. Mouse and guinea pig siRNA sequences and modifications used herein.

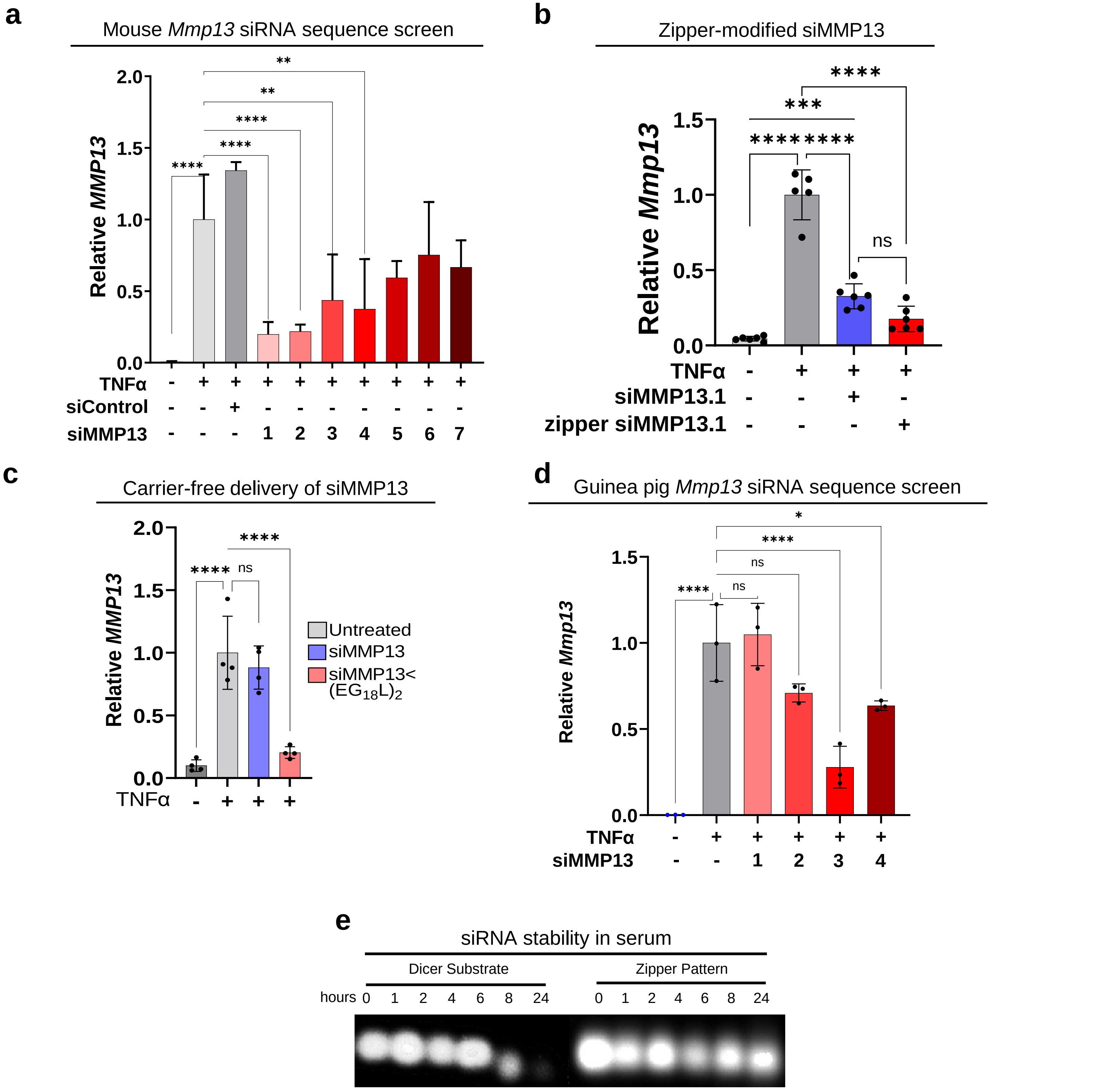

Supplementary Figure 13. MMP13 siRNA sequence screening across species, carrier free activity, and stability.

A-C) *Mmp13* mRNA was measured in TNFα-stimulated murine chondrogenic ATDC5 cells by RT-qPCR following transfection of candidate murine MMP13 siRNA sequences (**A**); Lipofectamine-mediated delivery of zipper-modified relative to unmodified siMMP13 sequences (**B**); Carrier-free silencing activity of siMMP13<$(EG_{18}L)_2$ (**C**) (N=3-4).

D) *Mmp13* mRNA was measured in TNFα-stimulated primary guinea pig chondrocytes by RT-qPCR following lipofectamine-mediated delivery of candidate guinea pig MMP13 siRNA sequences.

E) Serum stability (60% FBS, fetal bovine serum) at 37°C of 'zipper' modified or Dicer substrate mouse siMMP13.

Statistics markers: *P < 0.05, **P < 0.01, ***P < 0.001, ****P < 0.0001.

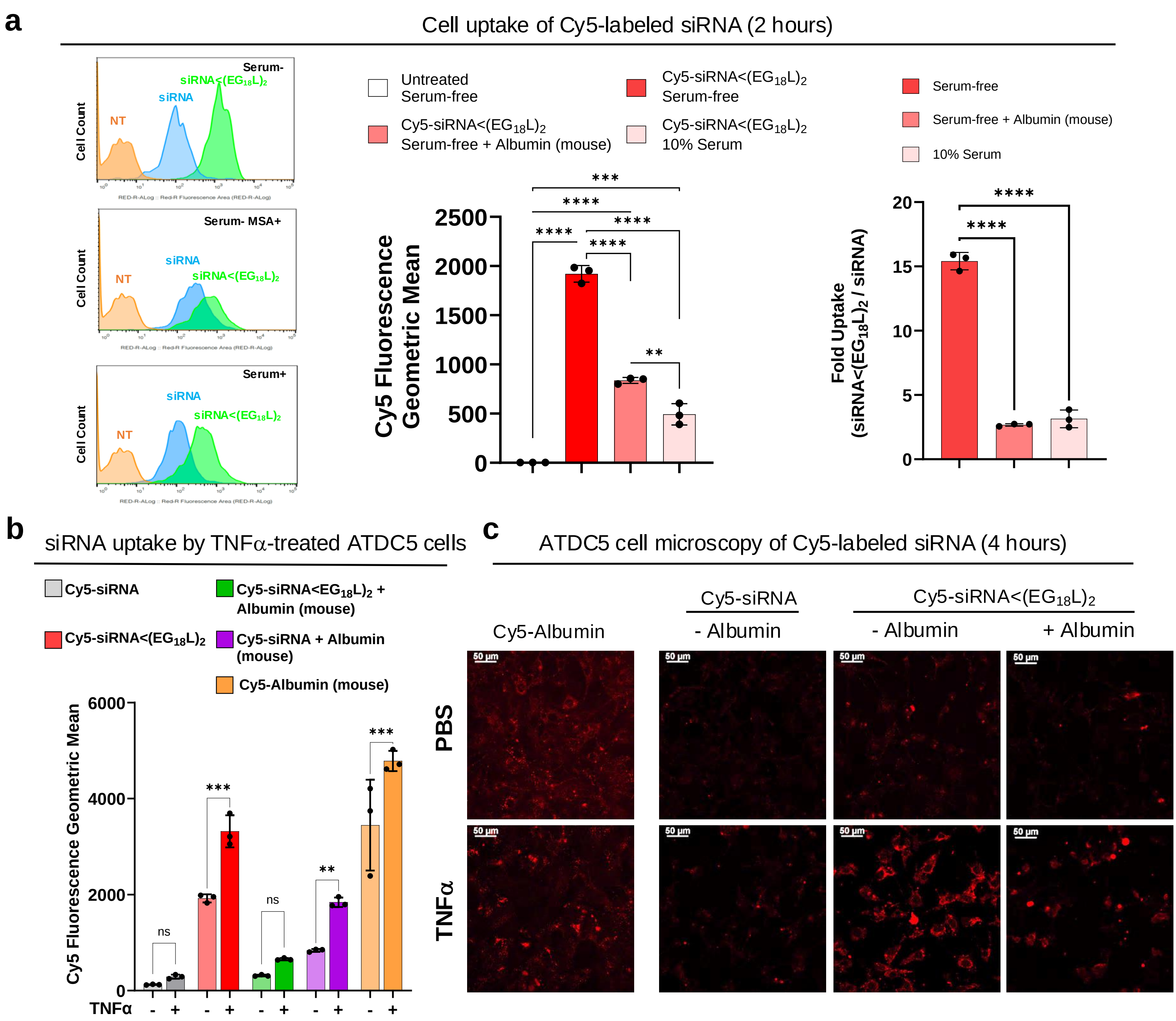

Supplementary Figure 14. Carrier-Free Mediated Cell Uptake in Chondrogenic Murine ATDC5 Cells.

A) Flow cytometry histograms of cellular uptake 2 hours after carrier-free treatment of Cy5-conjugated siRNA or siRNA<$(EG_{18}L)_2$ at a concentration of 100 nM in serum-free, serum-free + supplemented mouse serum albumin (10x, 1 μM), or serum-containing media. Data are quantified as fold change of siRNA<$(EG_{18}L)_2$/siRNA cell uptake in differing media conditions (N=3).

B) Quantification of cellular uptake by flow cytometry in ATDC5 cells treated with or without murine TNFα (20 ng/mL) (N=3).

C) Representative confocal microscopy images of ATDC5 cellular uptake (Cy5 fluorescence) after 4 hours of treatment with or without murine TNFα (20 ng/mL).

Statistics markers: *P < 0.05, **P < 0.01, ***P < 0.001, ****P < 0.0001.

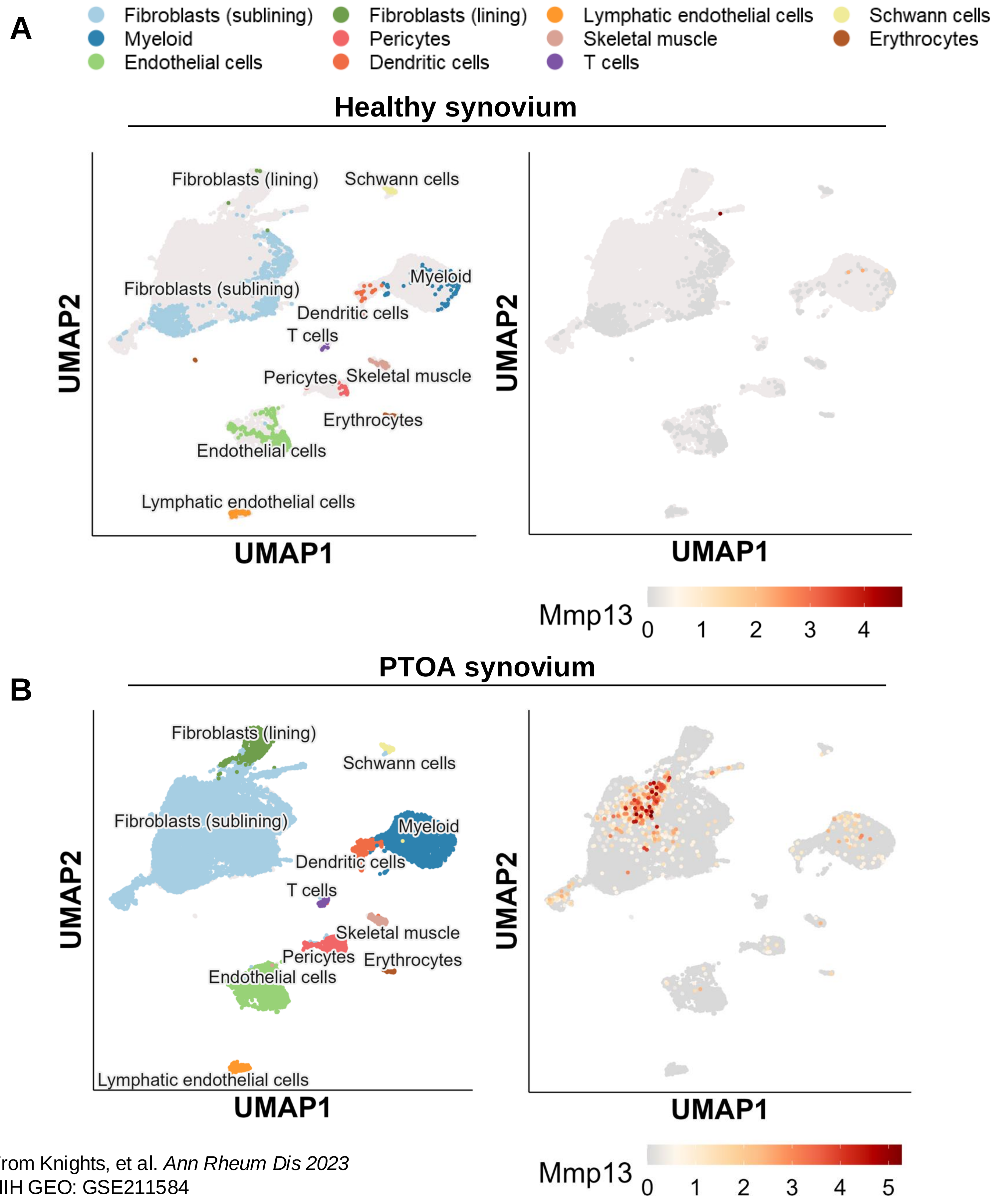

From Knights, et al. *Ann Rheum Dis 2023*
NIH GEO: GSE211584

Supplementary Figure 15. Mmp13 is upregulated in synovial fibroblasts and myeloid cells.
A) Distribution of cell populations in a healthy mouse synovium (left) with low levels of Mmp13 expression (right)
B) Distribution of cell populations in a noninvasive ACL rupture model PTOA mouse synovium (left) with increased Mmp13 expression (right)

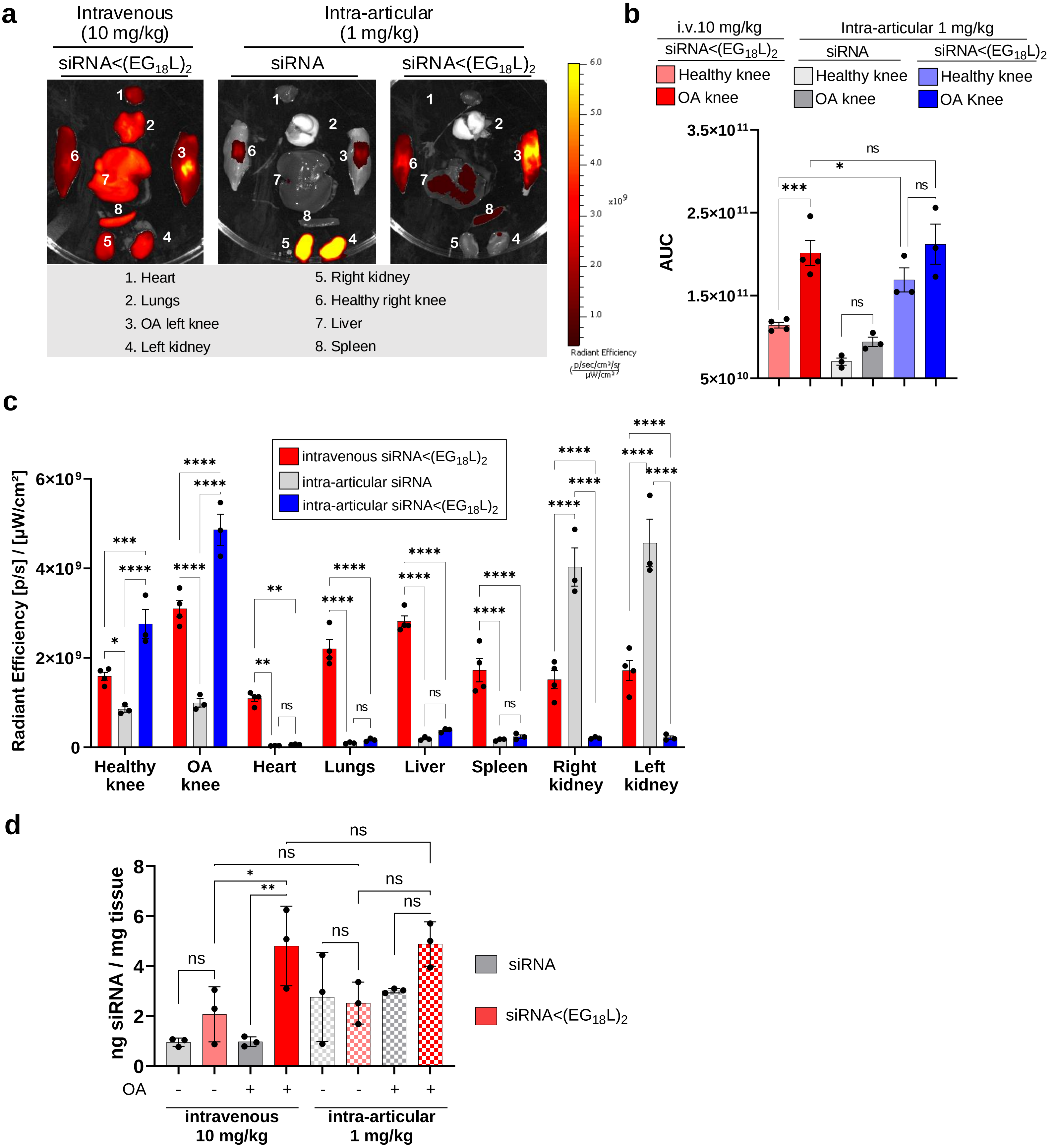

Supplementary Figure 16. Pharmacokinetic and biodistribution analyses of intra-venous vs. intra-articular routes.

A) Representative IVIS organ biodistribution images of all 3 groups at the 30-day endpoint.

B) AUC analysis for i.v. siRNA<($EG_{18}L$)$_2$ (10 mg/kg), intra-articular siRNA (1 mg/kg), and intra-articular siRNA<($EG_{18}L$)$_2$ (1 mg/kg) throughout the entire 30-day intravital IVIS retention study (N=3-4).

C) Quantification of organ biodistribution from images in A (N=3-4).

D) PNA hybridization assay showing ng/mg antisense strand of the siRNA per mg of knee joint tissue at 24 hrs (i.v.) or 48 hrs (i.a.) (N=3).

Statistics markers: *P < 0.05, **P < 0.01, ***P < 0.001, ****P < 0.0001. Healthy indicates a non-loaded contralateral knee.

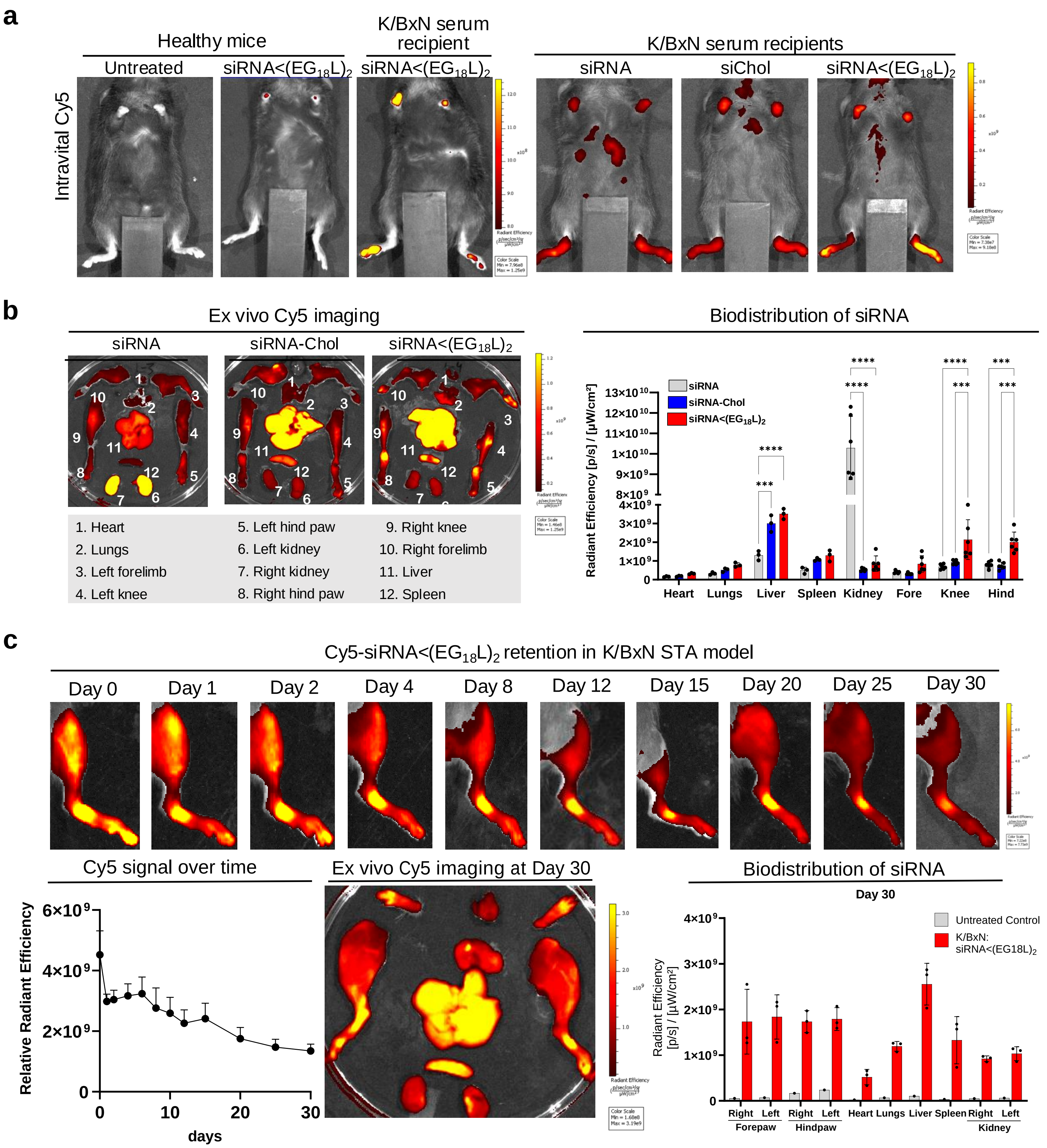

Supplementary Figure 17. Biodistribution and pharmacokinetics of K/BxN STA model.
A-B) Cy5-siRNA<$(EG_{18}L)_2$, Cy5-siRNA-Chol, or Cy5-siRNA was delivered i.v. (1 mg/kg) to healthy and K/BxN STA mice and assessed 24 hrs later. Intravital Cy5 measurements in healthy and K/BxN STA mice in each treatment group (A). Organ Cy5 biodistribution was measured ex vivo (B) (N=3 mice).
C) Longevity of Cy5-siRNA<$(EG_{18}L)_2$ retention in K/BxN knees following single dose of Cy5-siRNA<$(EG_{18}L)_2$ (10mg/kg, i.v.) was assessed by longitudinal intravital imaging, and endpoint imaging of knees and organs done ex vivo on day 30 (N=3 mice). Radiant efficiency over time displayed as mean + SEM.
Statistics markers: *$P < 0.05$, **$P < 0.01$, ***$P < 0.001$, ****$P < 0.0001$.

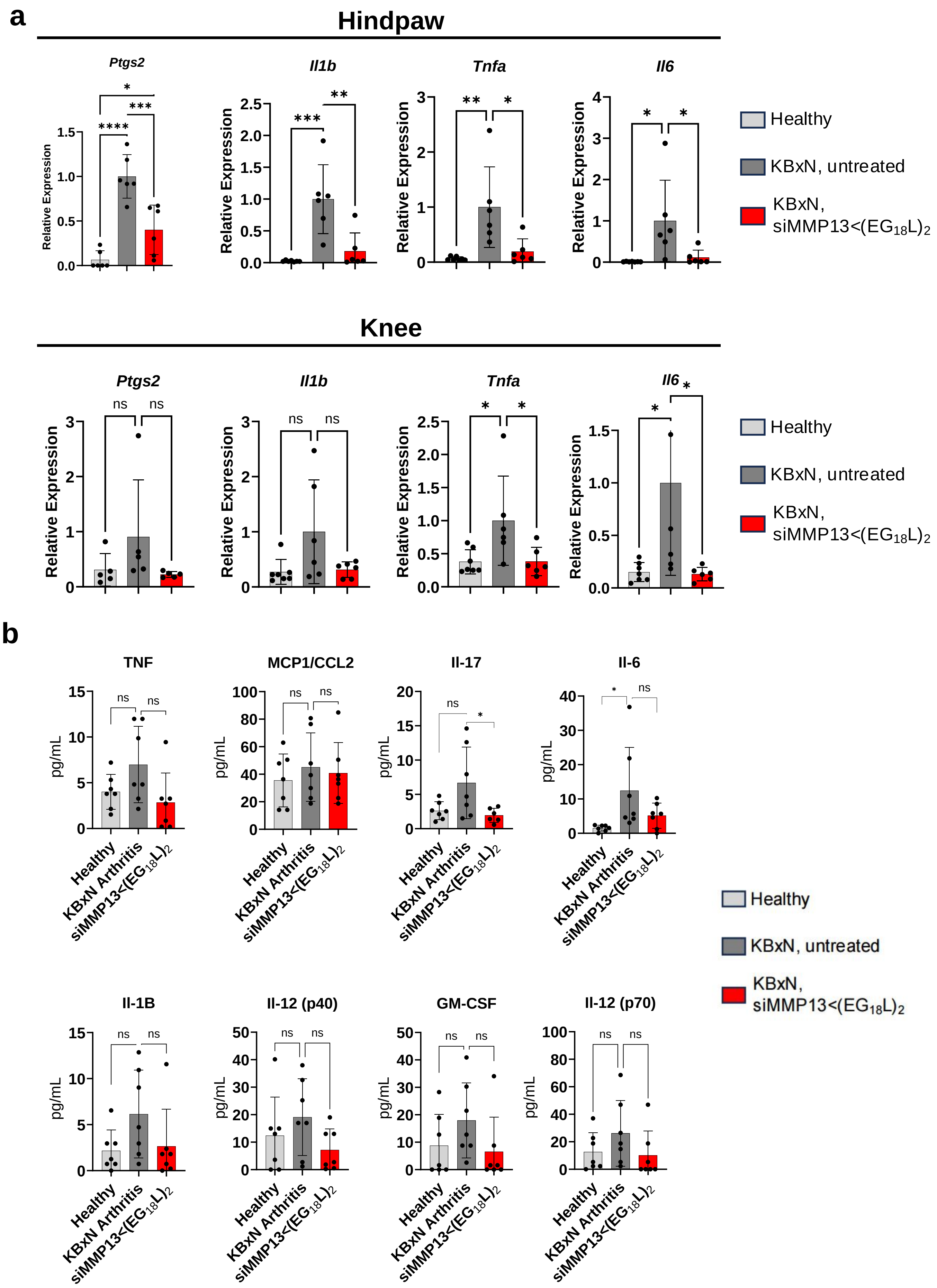

Supplementary Figure 18. Molecular analysis of K/BxN therapeutic study (10 mg/kg).

A) qRT-PCR of genes of importance in rheumatoid arthritis in the K/BxN therapeutic study at the day 10 endpoint (N=5-7).

B) Multiplex Luminex analyses for analytes in serum in the K/BxN therapeutic study at the day 10 endpoint (N=5-7).

Statistics markers: *P < 0.05, **P < 0.01, ***P < 0.001, ****P < 0.0001.

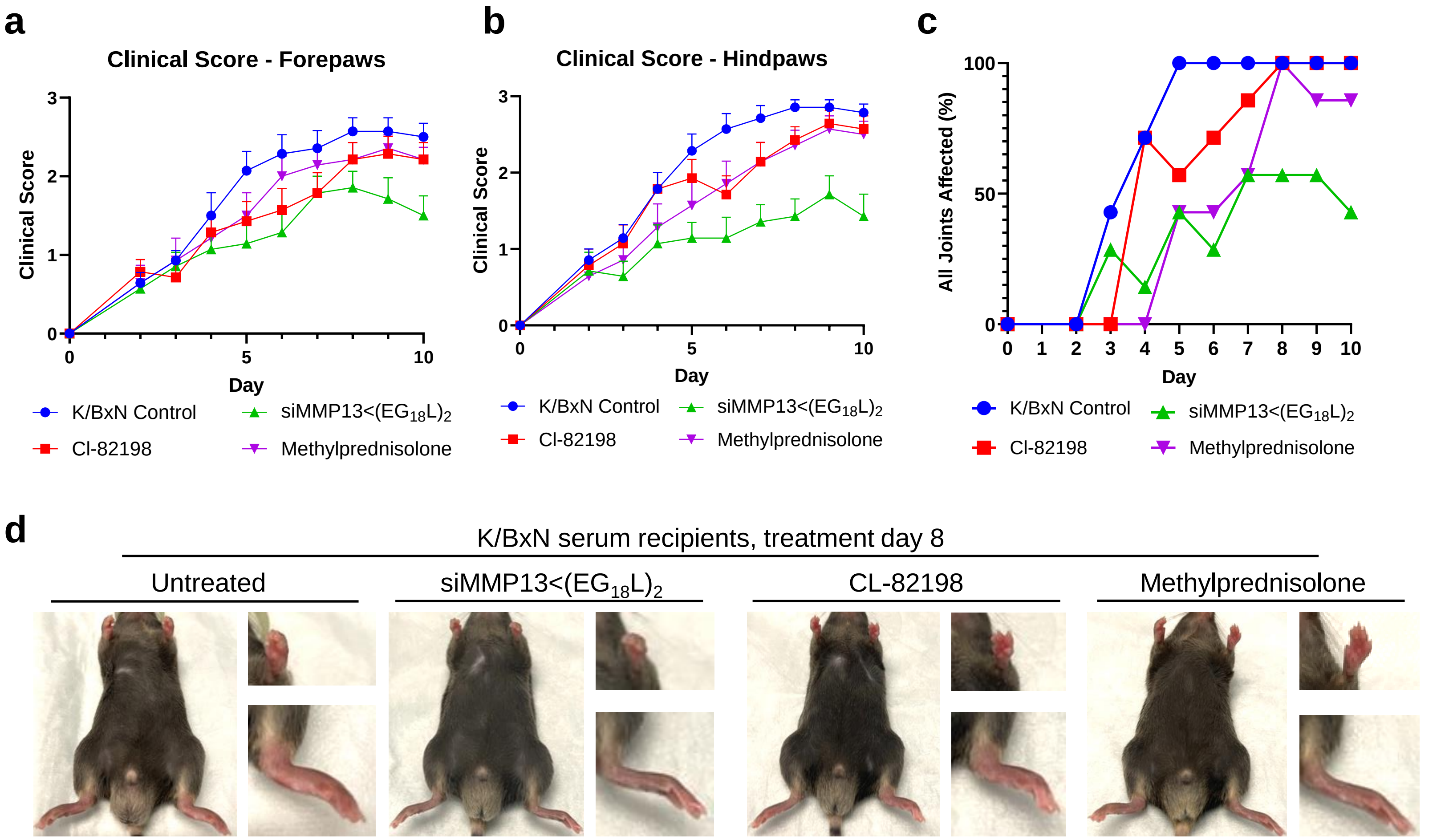

Supplementary Figure 19. Further characterization of K/BxN therapeutic study.
A-B) Clinical score of forepaws (A) and hindpaws (B) (N=7 mice). Data displayed as mean + SEM.
C) Percentage of mice exhibiting affliction of all paw joints following K/BxN serum transfer (N=7 mice).
D) Representative images showing inflammation / swelling in limbs of K/BxN serum recipients on treatment day 8 (10 days after serum transfer). Insets show higher power image of forepaws (upper inset) and hind paws (lower inset). Images were used for clinical scoring, with a maximal clinical score of 3 per paw, total score of 12 per mouse.

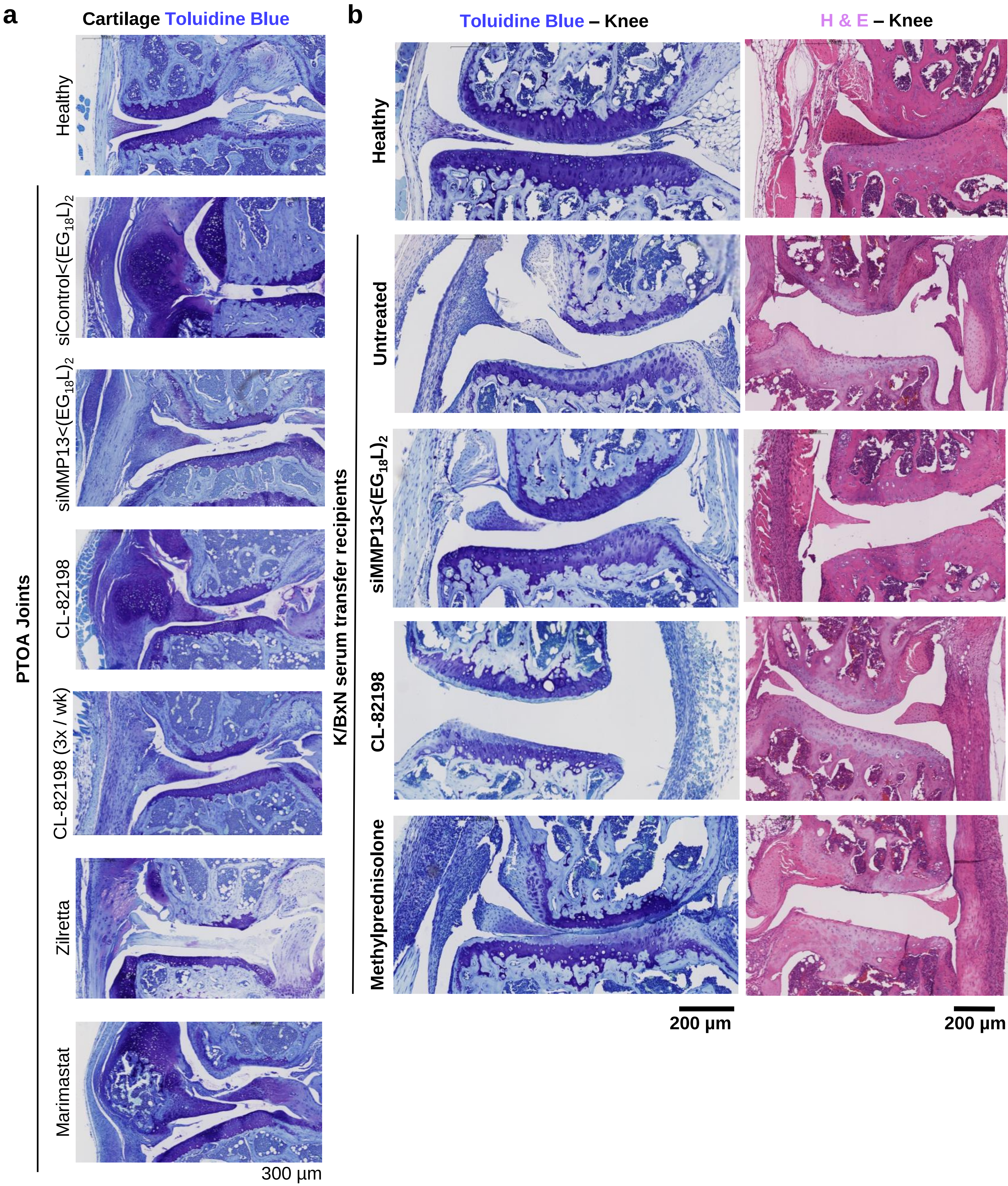

Supplementary Figure 20. Representative lower magnification knee joint histology for PTOA and K/BxN therapeutic studies.

A) Lower magnification of medial joint toluidine blue histology for the PTOA therapeutic study. Healthy indicates a non -loaded contralateral knee.

B) Toluidine blue and H&E of knee joint tissue for the K/BxN therapeutic study.

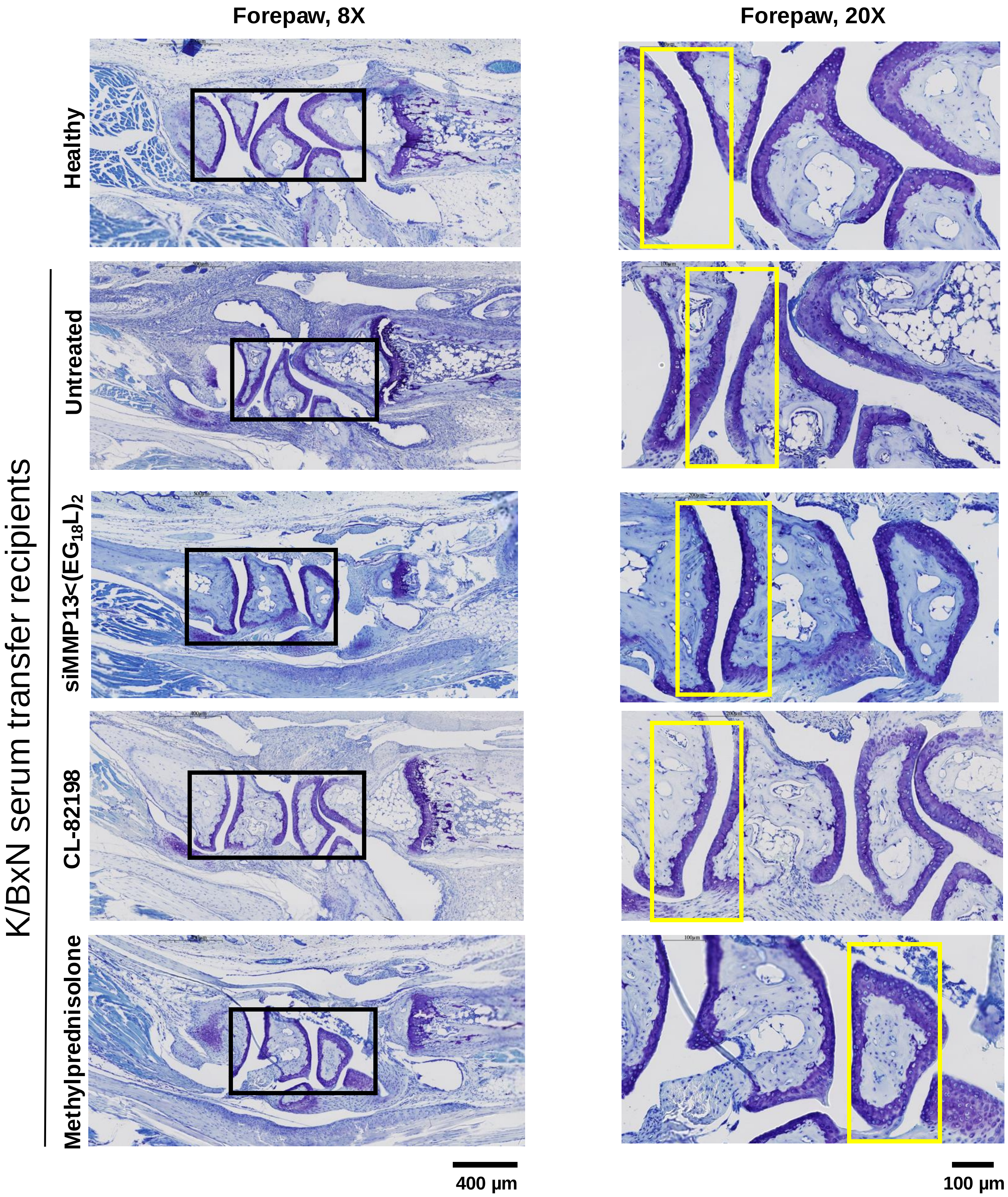

Supplementary Figure 21. Toluidine Blue of forepaw / wrist cartilage of K/BxN serum recipients. Panels on left show 8X magnification. Black rectangles in left panels are the areas shown at higher magnification (20X) in right panels. Yellow rectangles in right panels indicate the areas of higher magnification shown in extended data figures.

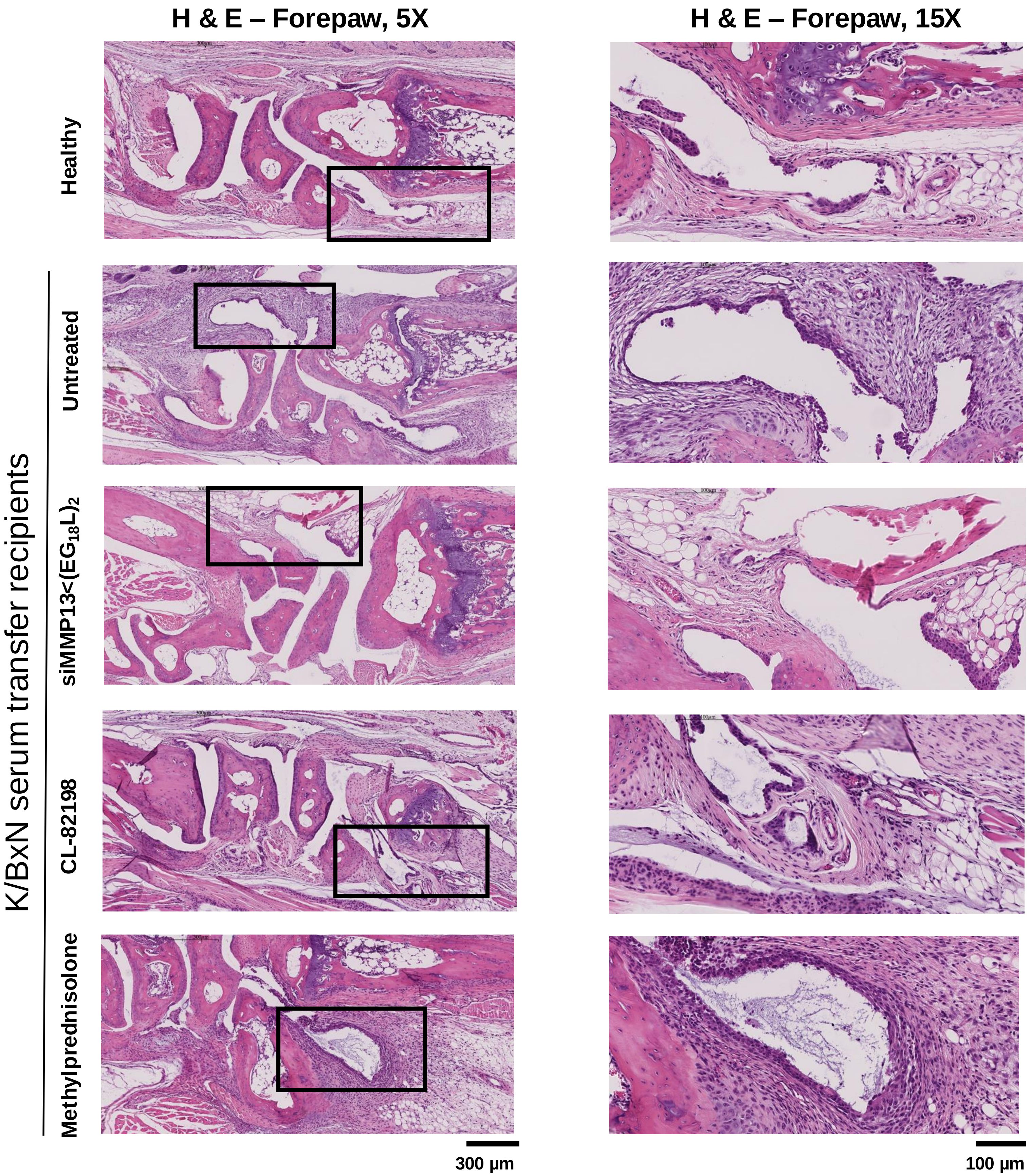

Supplementary Figure 22. H&E of forepaw/wrist tissue for the K/BxN therapeutic study.

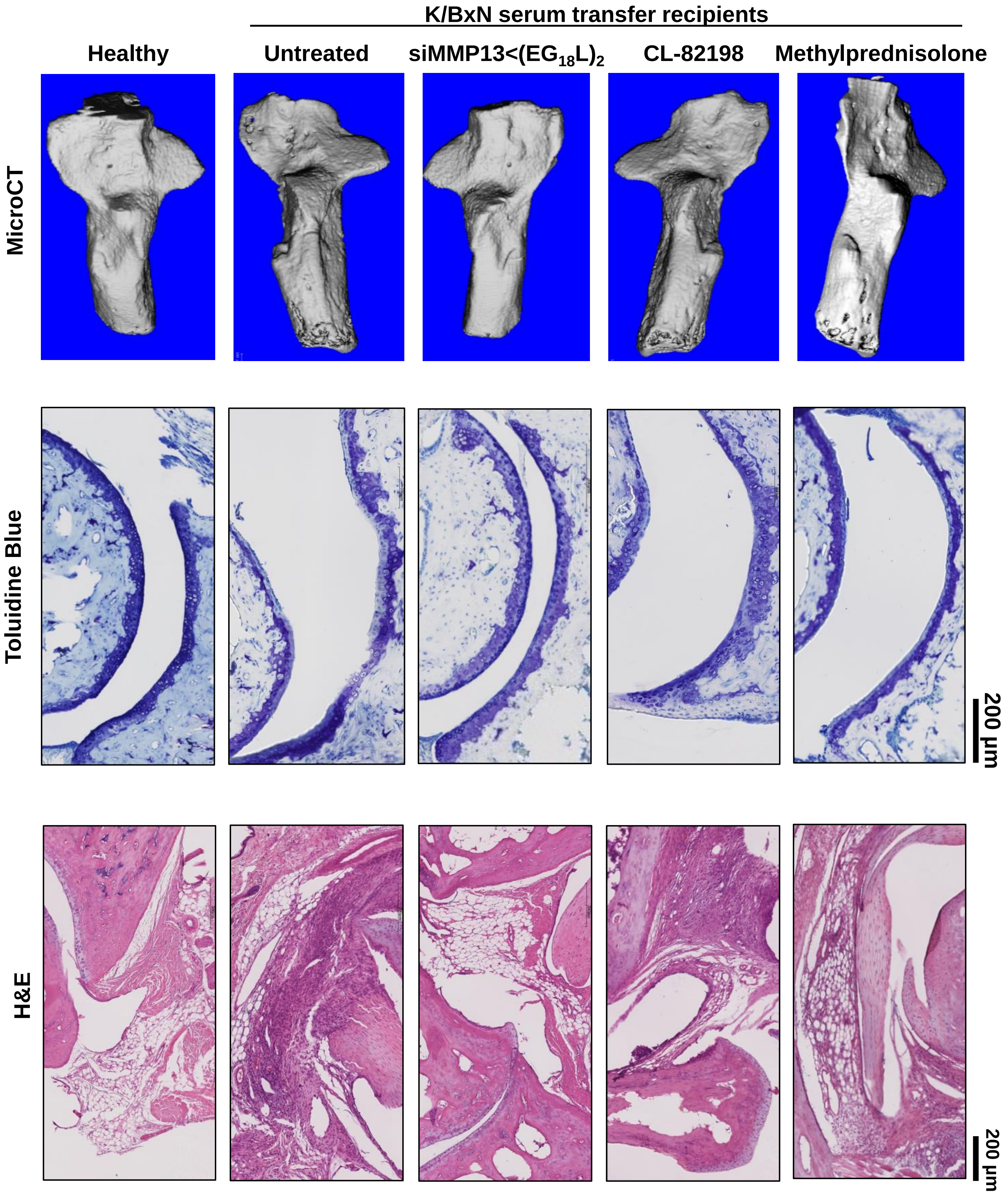

Supplementary Figure 23. Bone loss and ankle joint assessments in K/BxN therapeutic study. Top: 3D reconstructions of the calcaneus bone (used for microCT parameter quantification). Middle: lower magnification Toluidine Blue of hindpaw/ankle cartilage. Bottom: lower magnification H&E of hindpaw/ankle tissue.

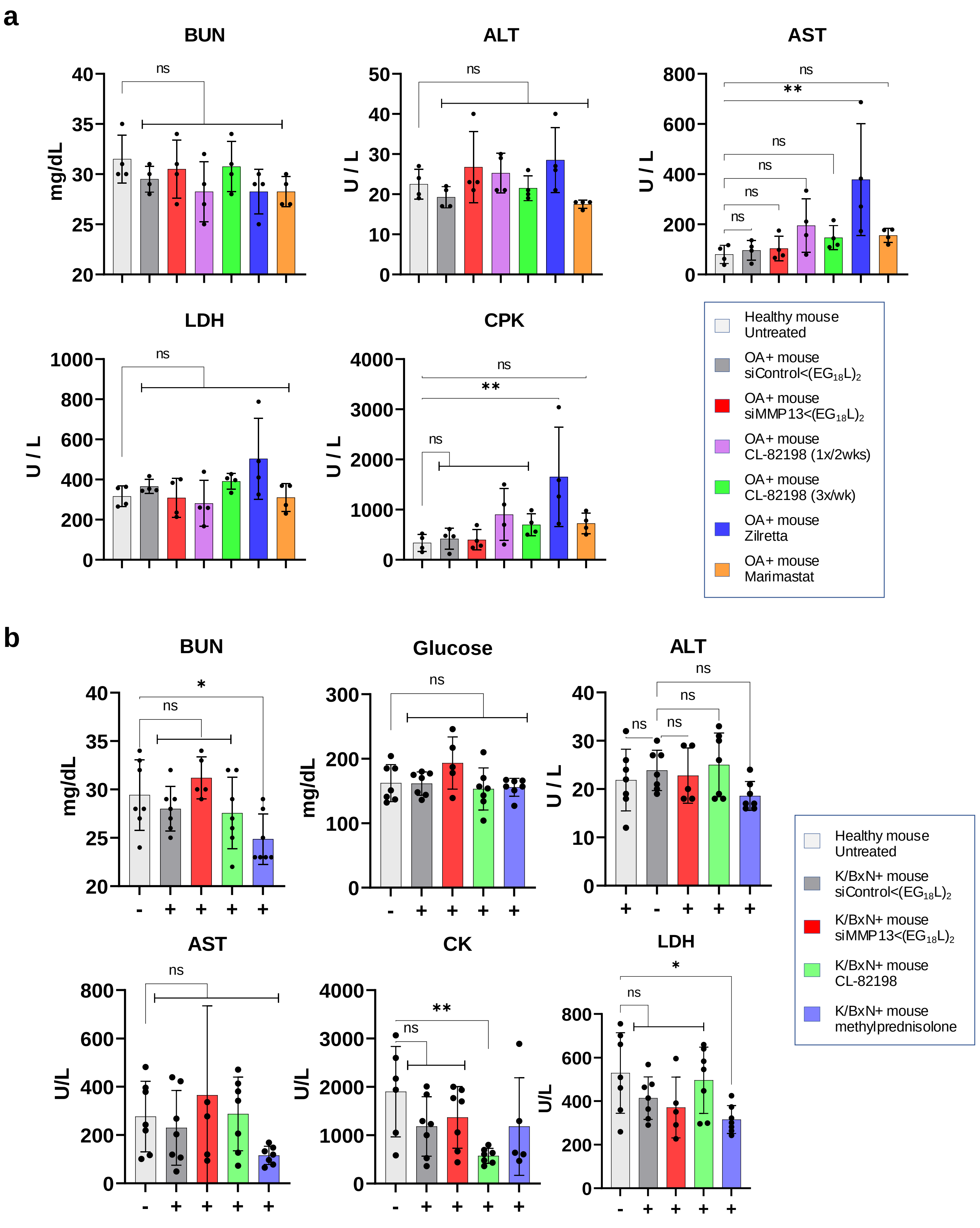

Supplementary Figure 24. Systemic toxicity analyses in K/BxN and PTOA model therapeutic studies.
A) Toxicology analyses in serum in the PTOA therapeutic study at the endpoint (N=4 mice).
B) Toxicology analyses in serum in the K/BxN therapeutic study at the endpoint (N=7 mice).
Statistics markers: *P < 0.05, **P < 0.01, ***P < 0.001, ****P < 0.0001.

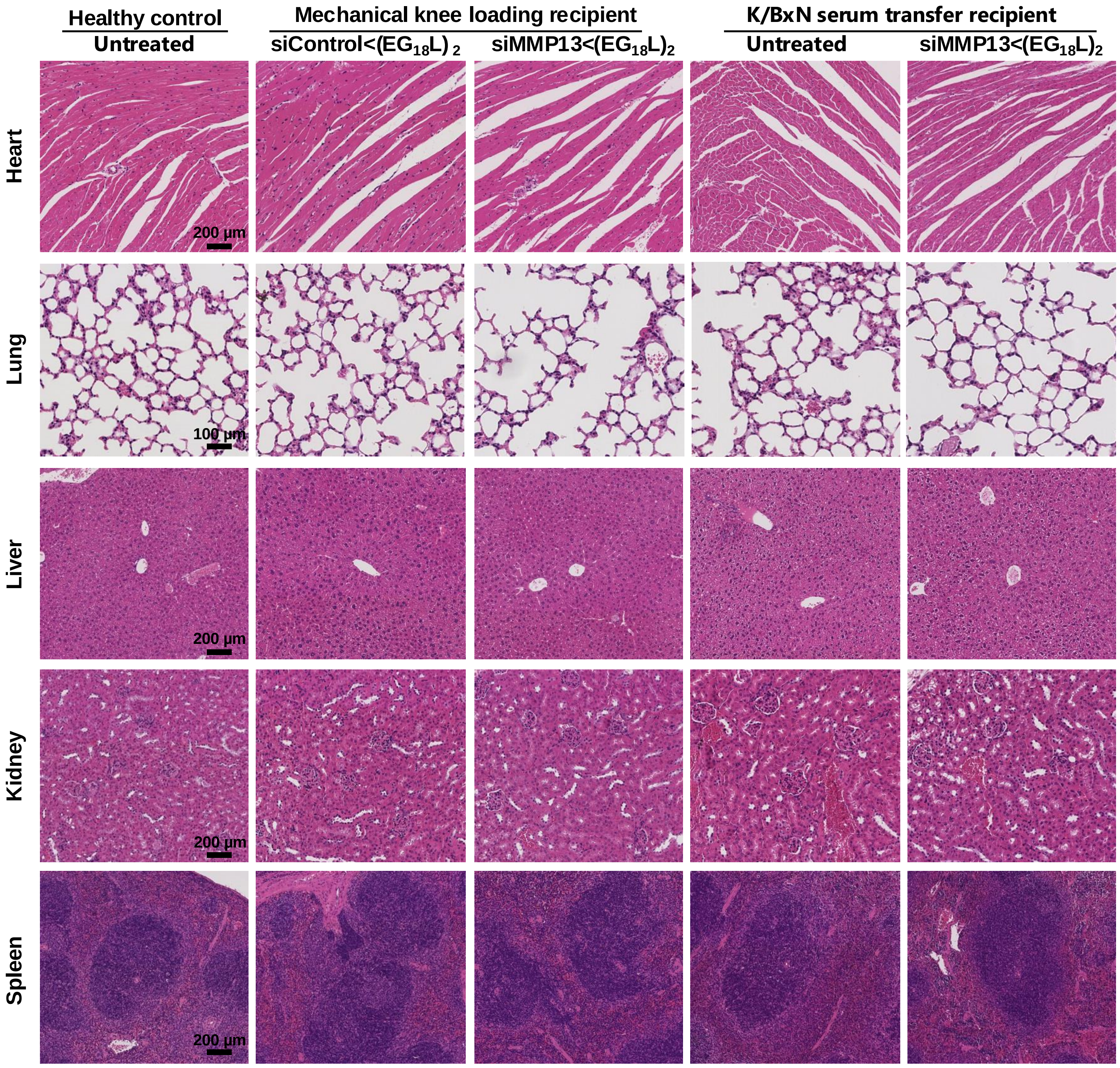

Supplementary Figure 25. H&E-stained sections of organs (liver, kidney, lung, heart, and spleen) from mice enrolled in the different treatment groups in the PTOA and K/BxN therapeutic studies. Organs were harvested at the end of the study for analysis, and a healthy (no disease, no treatment) mouse is included as a control.

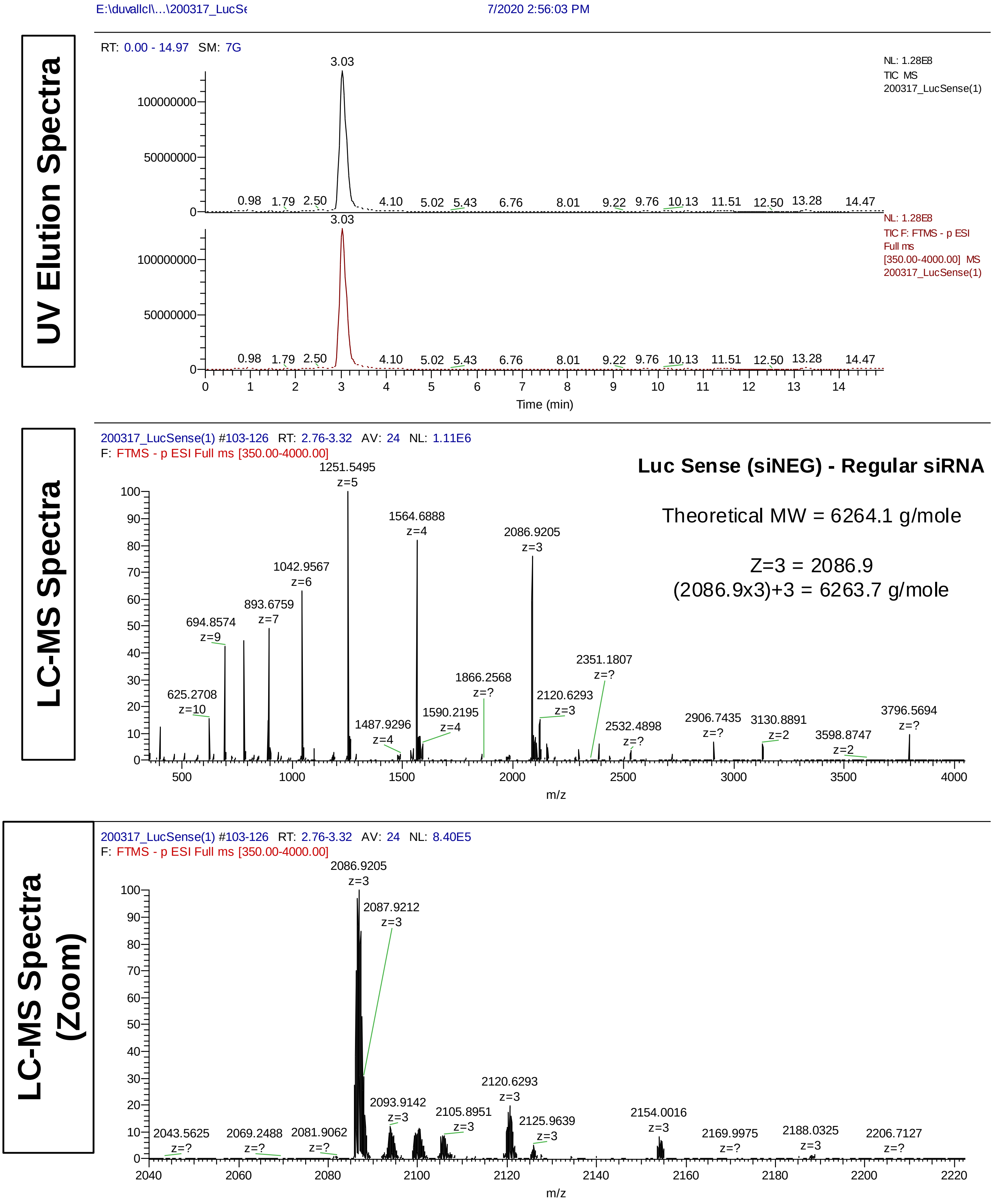

Supplementary Figure 26. LC-MS characterization of control sense siRNA sequence.

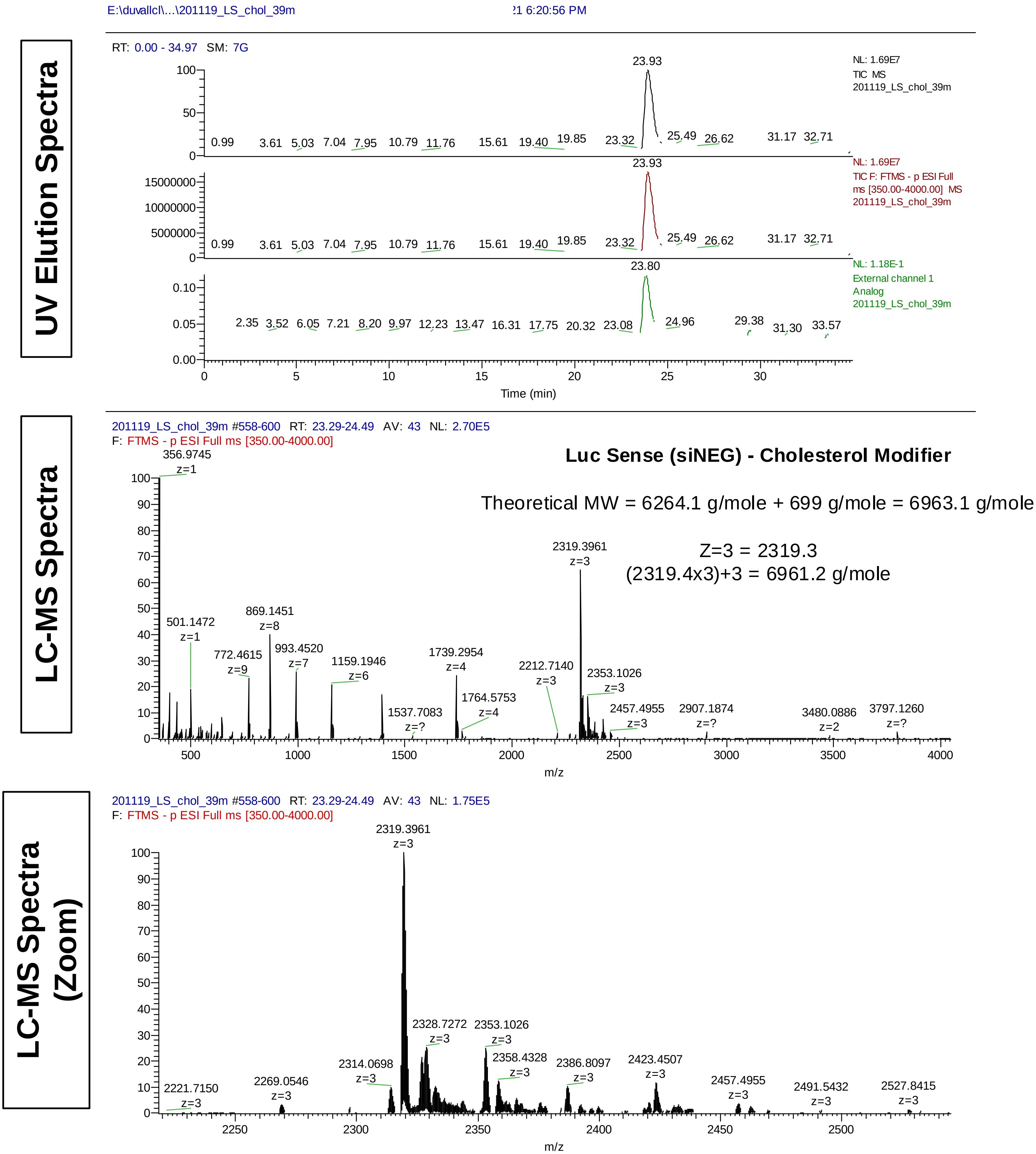

Supplementary Figure 27. LC-MS characterization of control sense – cholesterol modifier siRNA sequence.

UV Elution Spectra

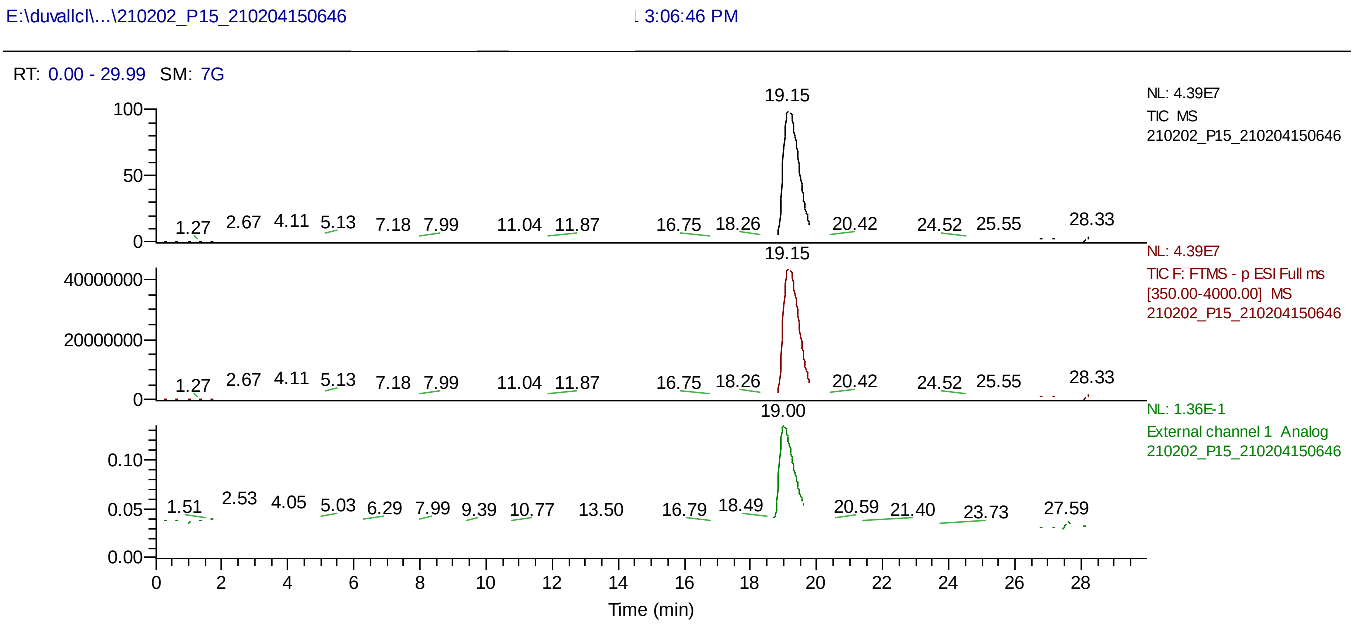

LC-MS Spectra

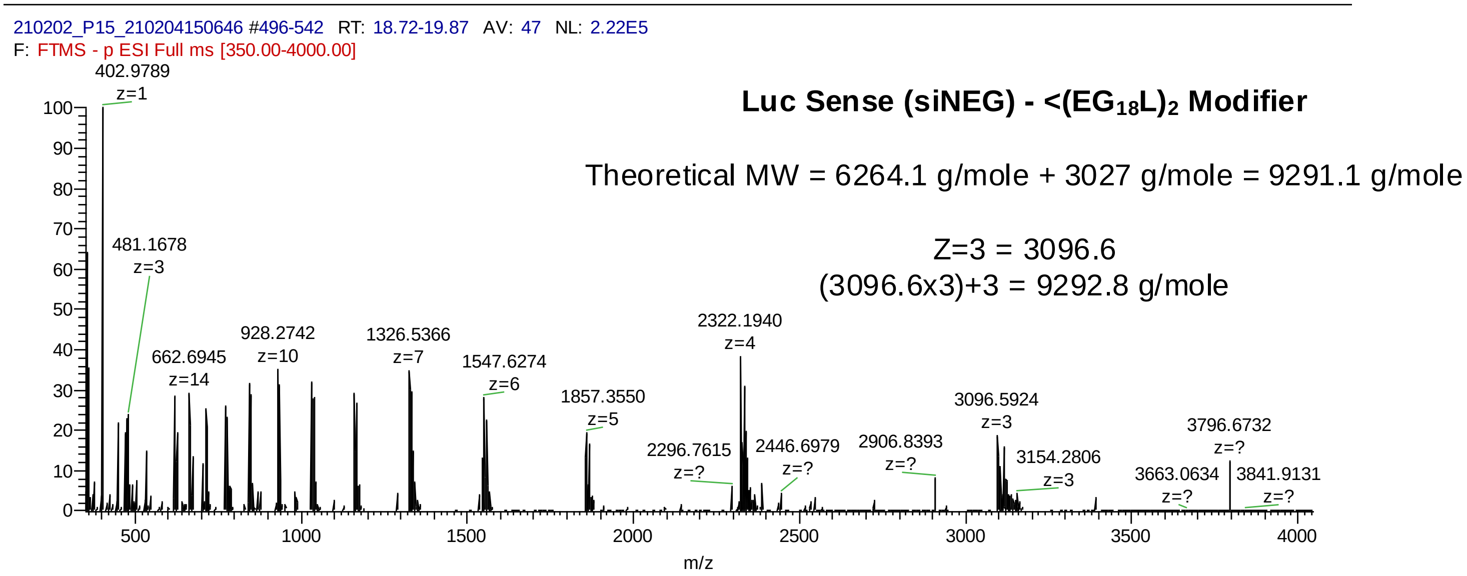

LC-MS Spectra (Zoom)

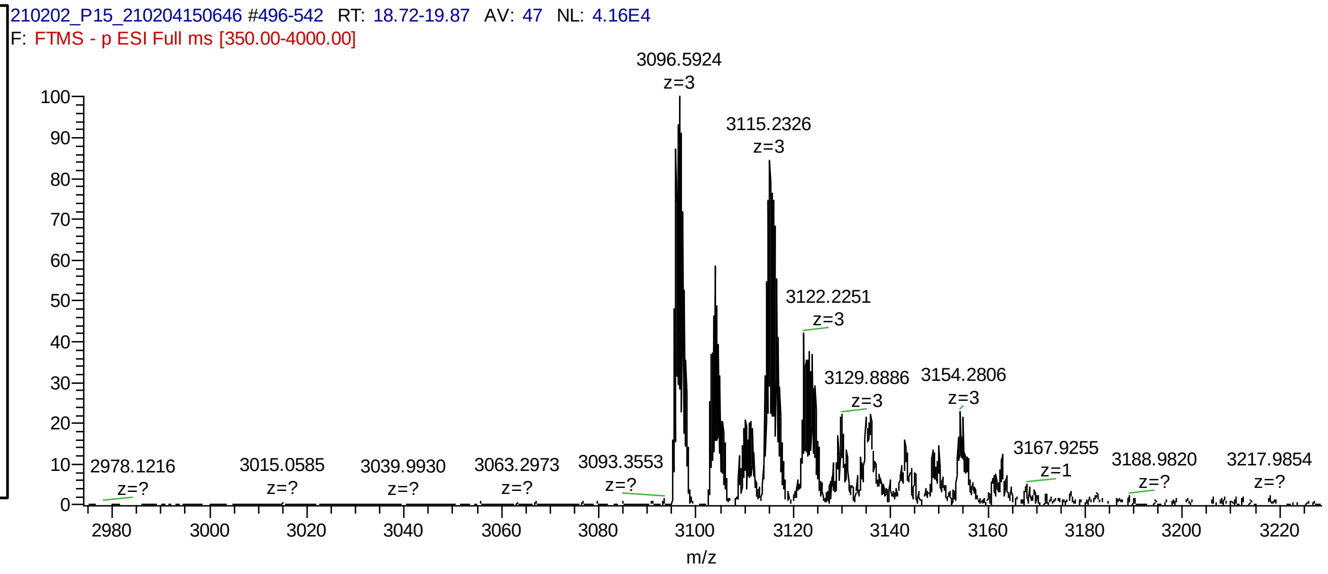

Supplementary Figure 28. LC-MS characterization of control sense – <($EG_{18}L$)$_2$ modifier siRNA sequence.

UV Elution Spectra

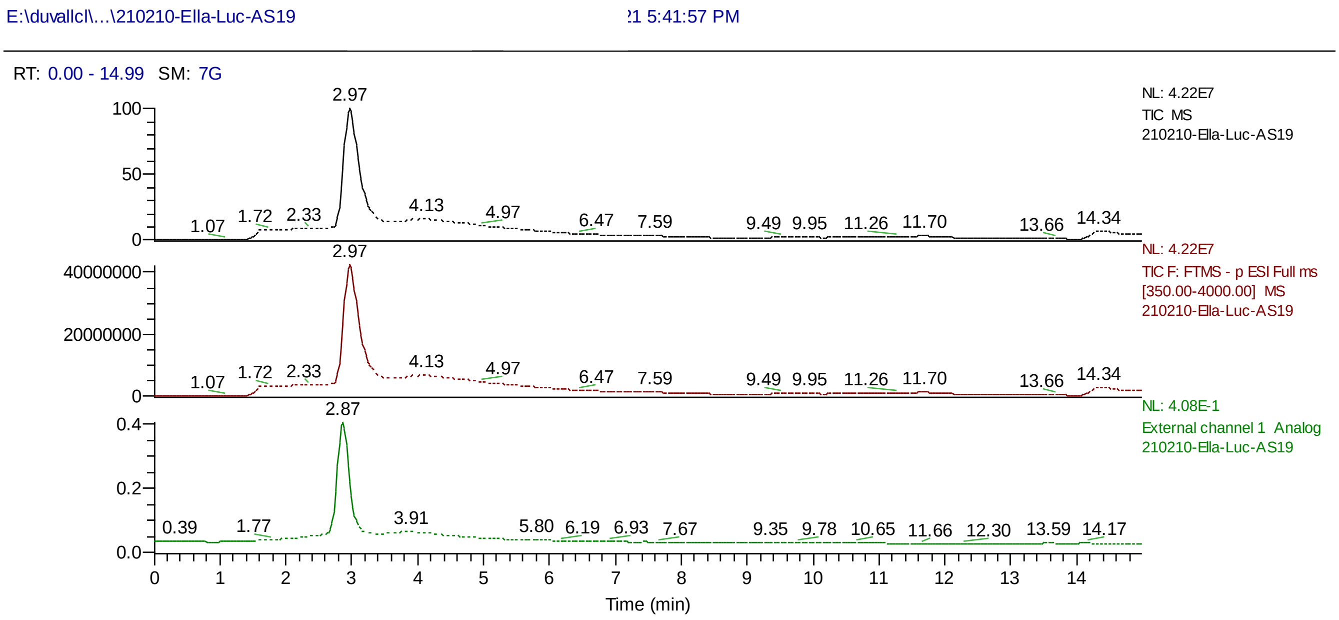

LC-MS Spectra

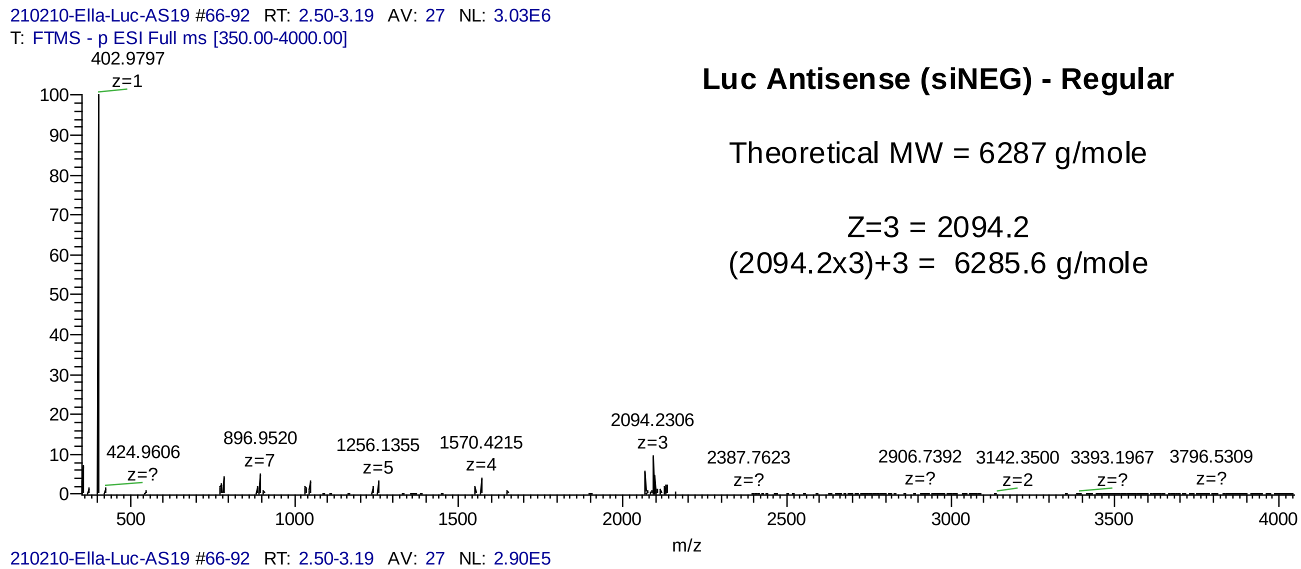

LC-MS Spectra (Zoom)

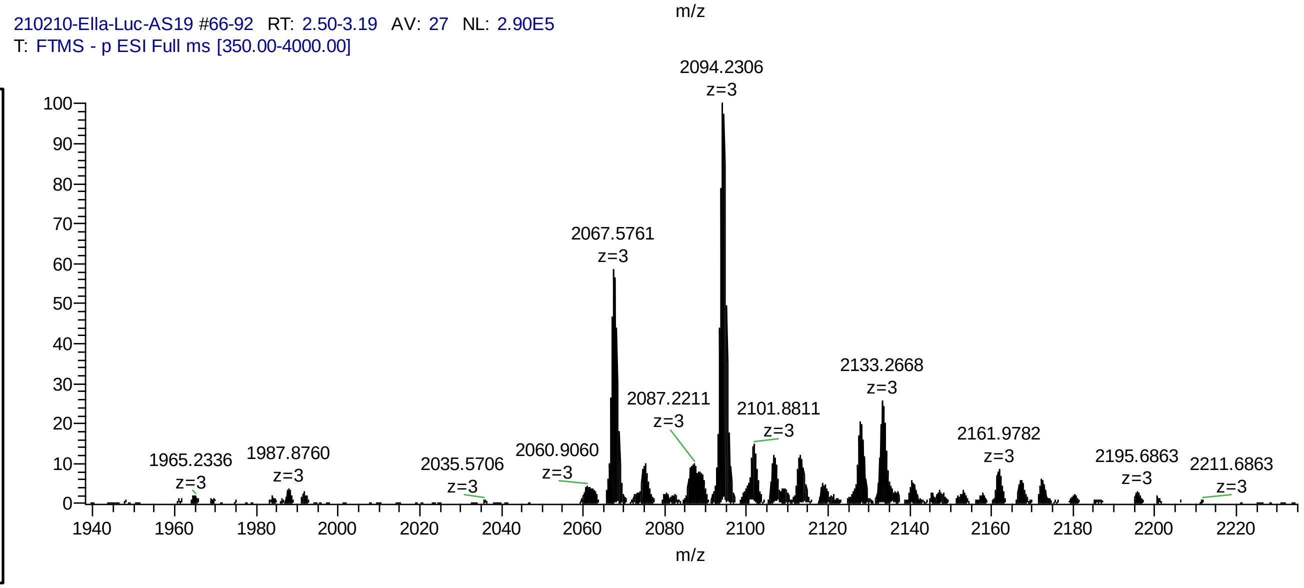

Supplementary Figure 29. LC-MS characterization of control antisense – siRNA sequence.

UV Elution Spectra

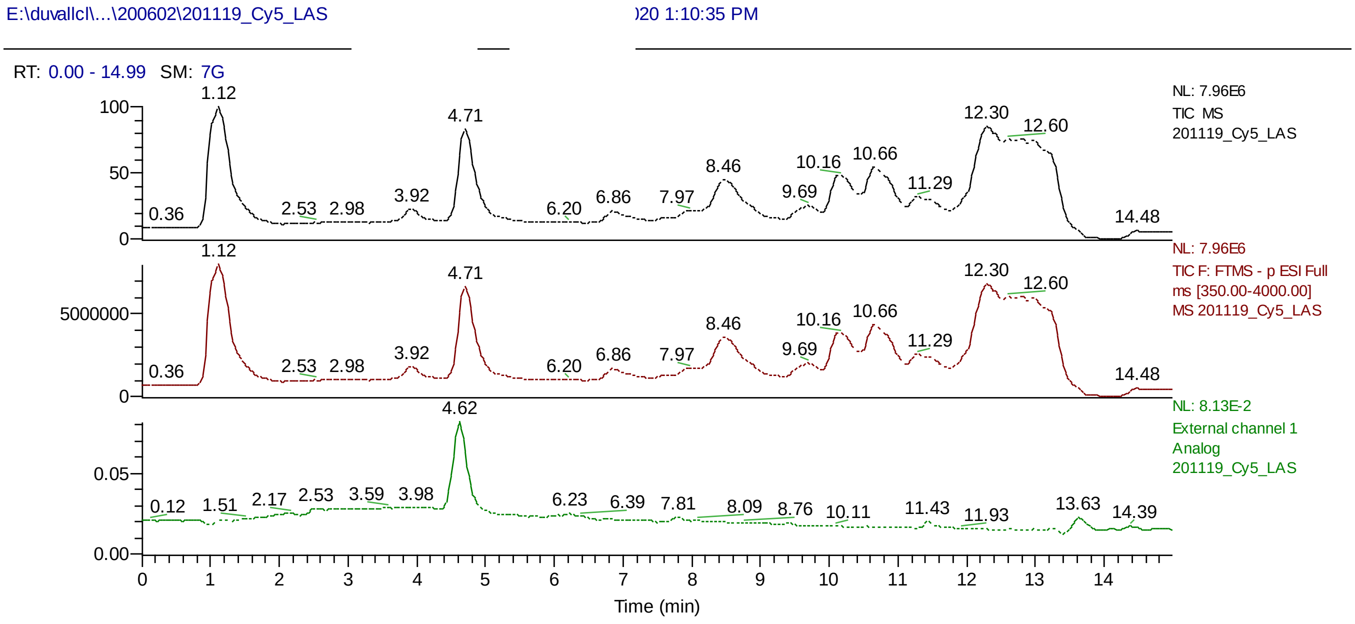

LC-MS Spectra

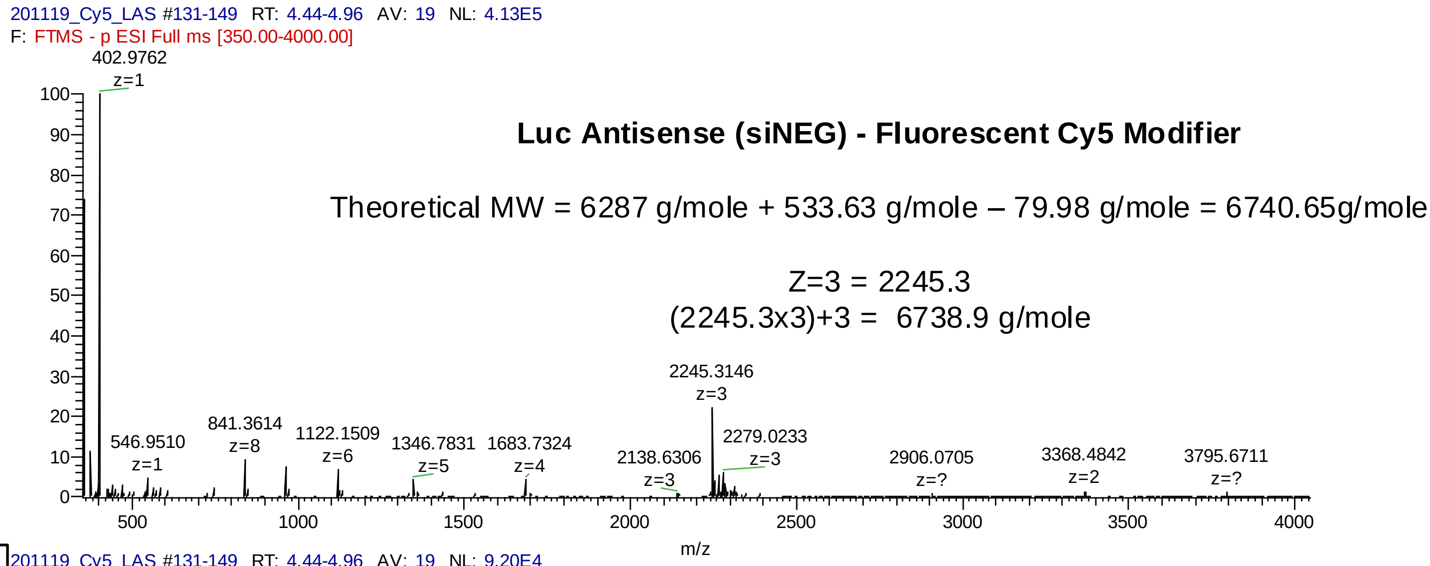

LC-MS Spectra (Zoom)

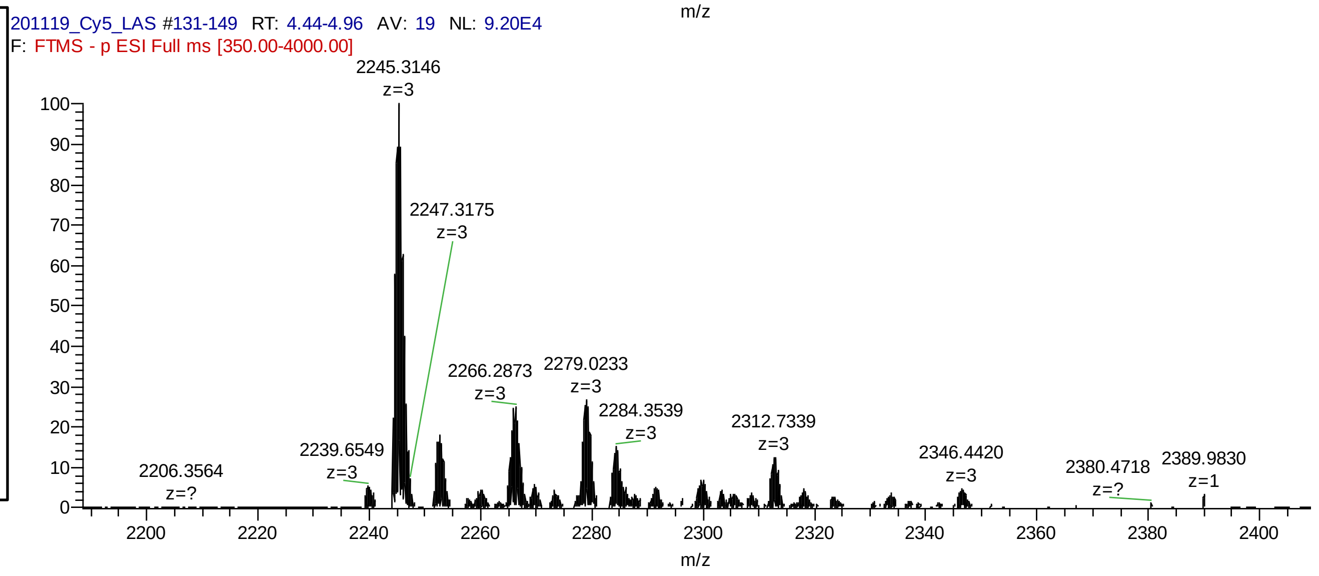

Supplementary Figure 30. LC-MS characterization of control antisense – Cy5 modifier siRNA sequence.

UV Elution Spectra

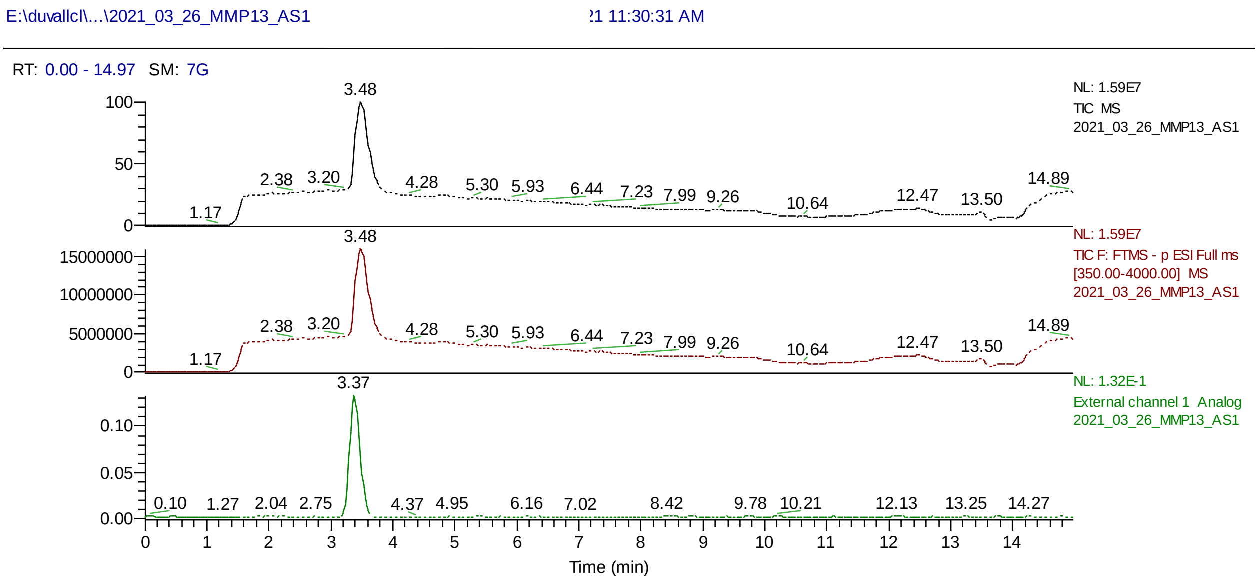

LC-MS Spectra

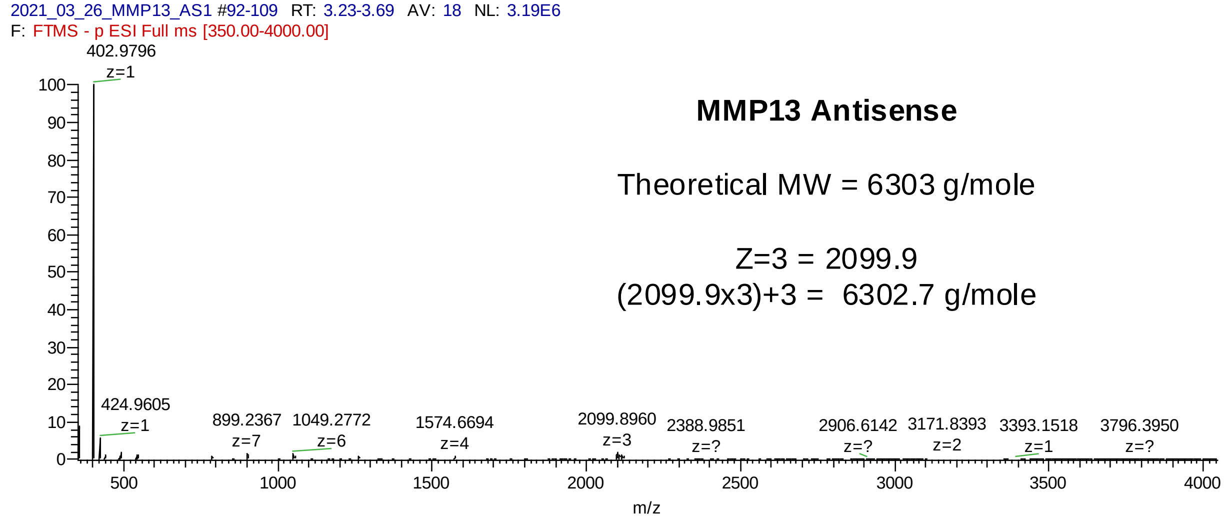

LC-MS Spectra (Zoom)

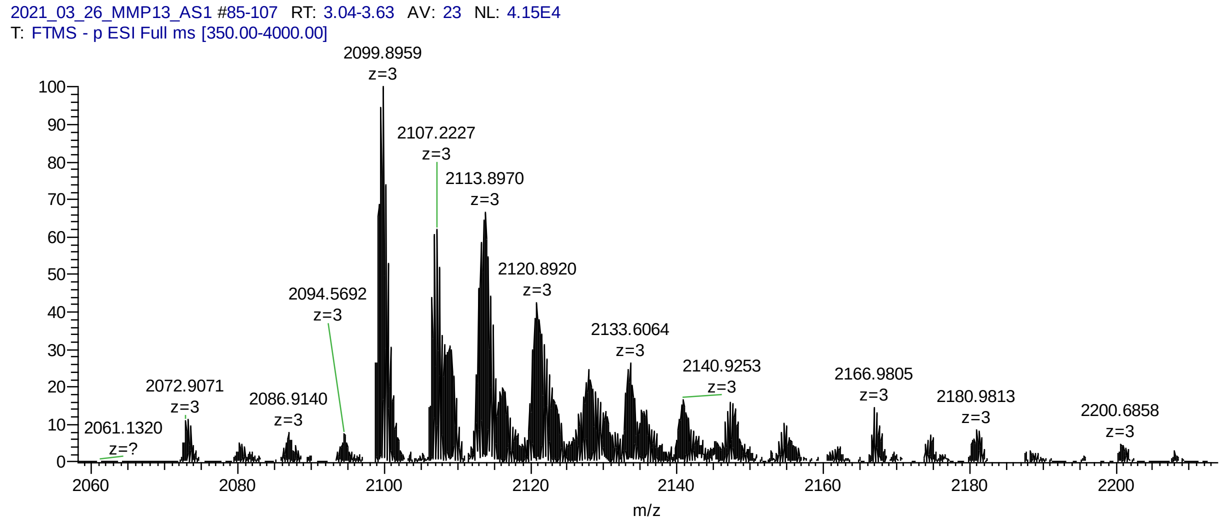

Supplementary Figure 31. LC-MS characterization of mouse MMP13 antisense – siRNA sequence.

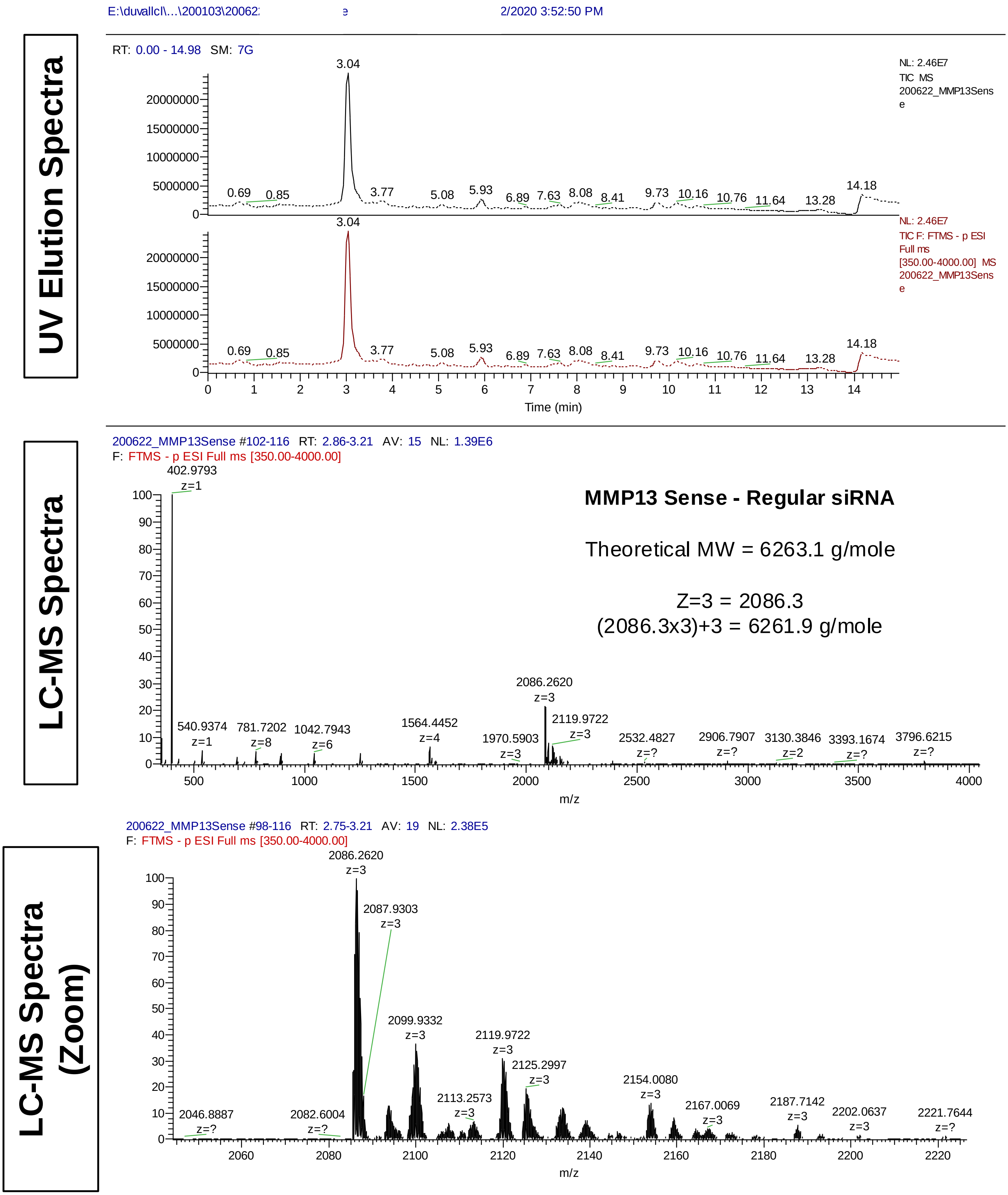

Supplementary Figure 32. LC-MS characterization of mouse MMP13 sense – siRNA sequence.

UV Elution Spectra

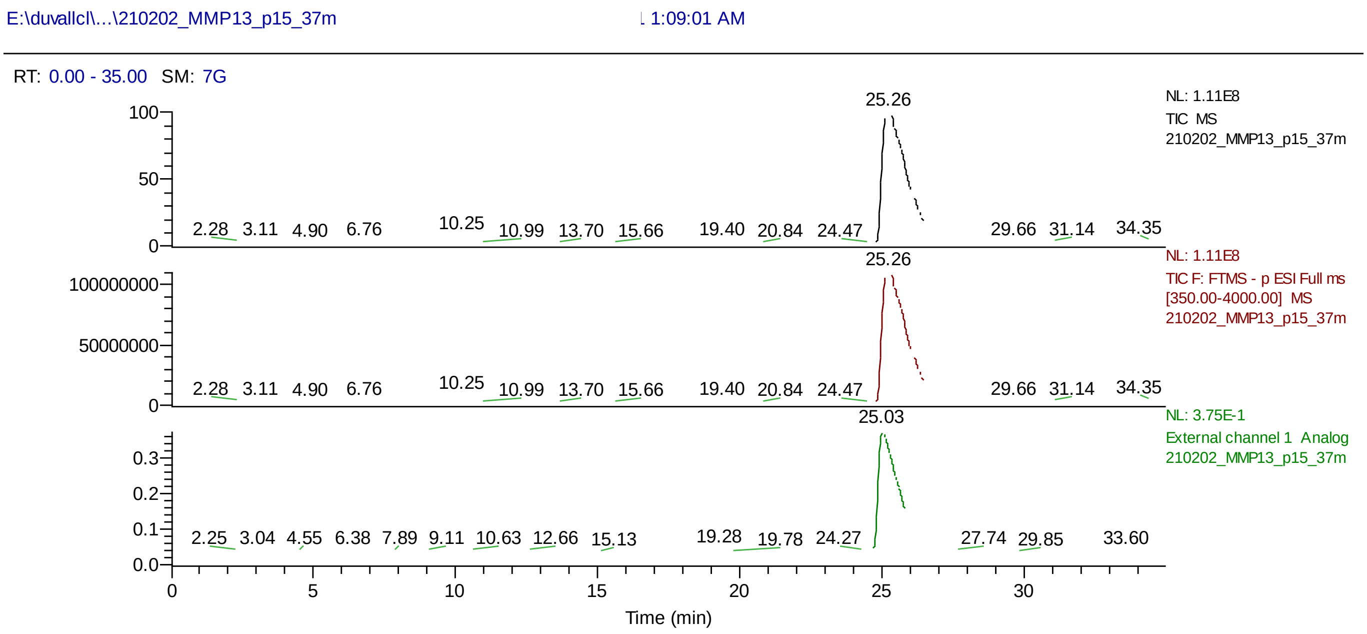

LC-MS Spectra

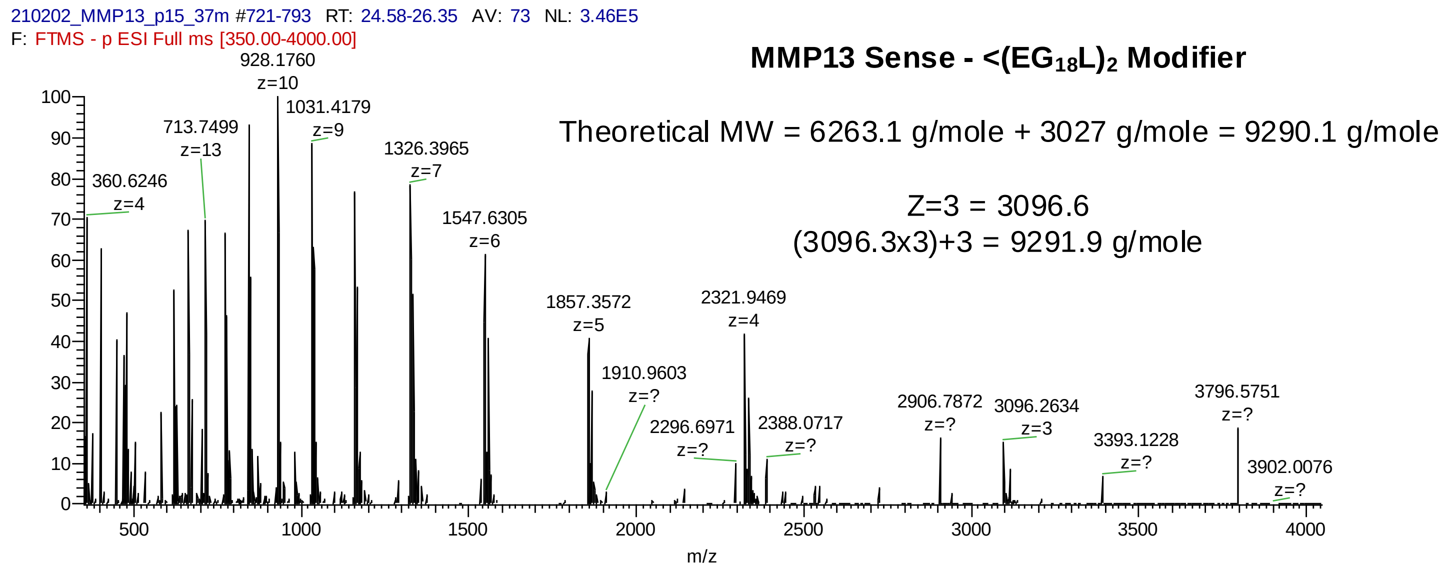

LC-MS Spectra (Zoom)

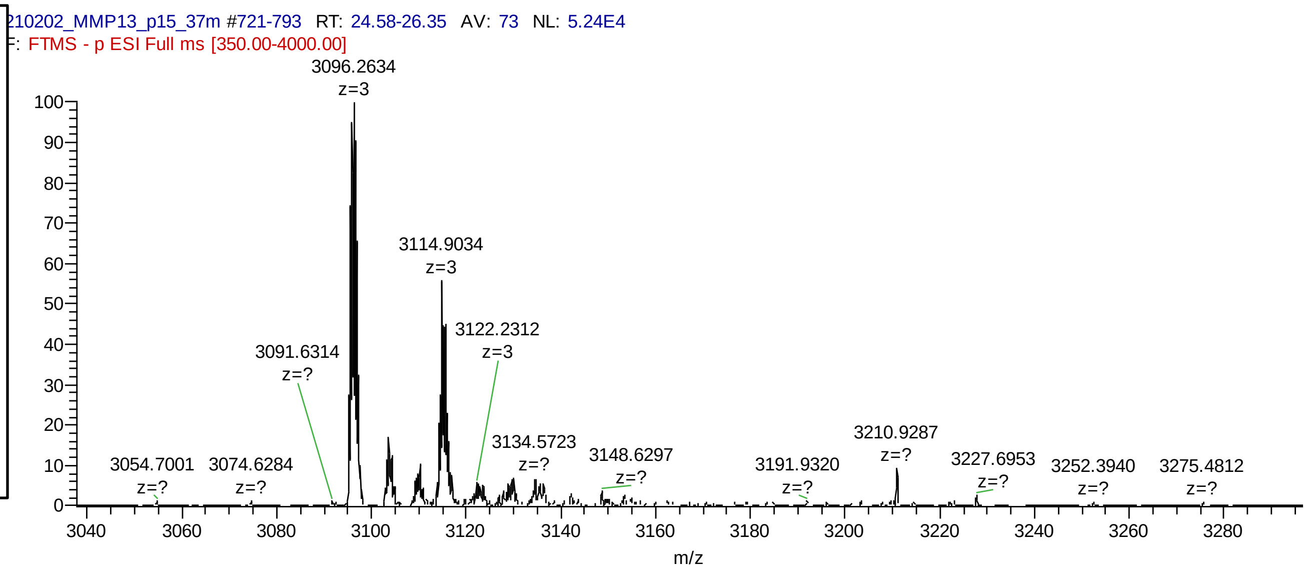

Supplementary Figure 33. LC-MS characterization of mouse MMP13 sense – <(EG$_{18}$L)$_2$ modifier siRNA sequence

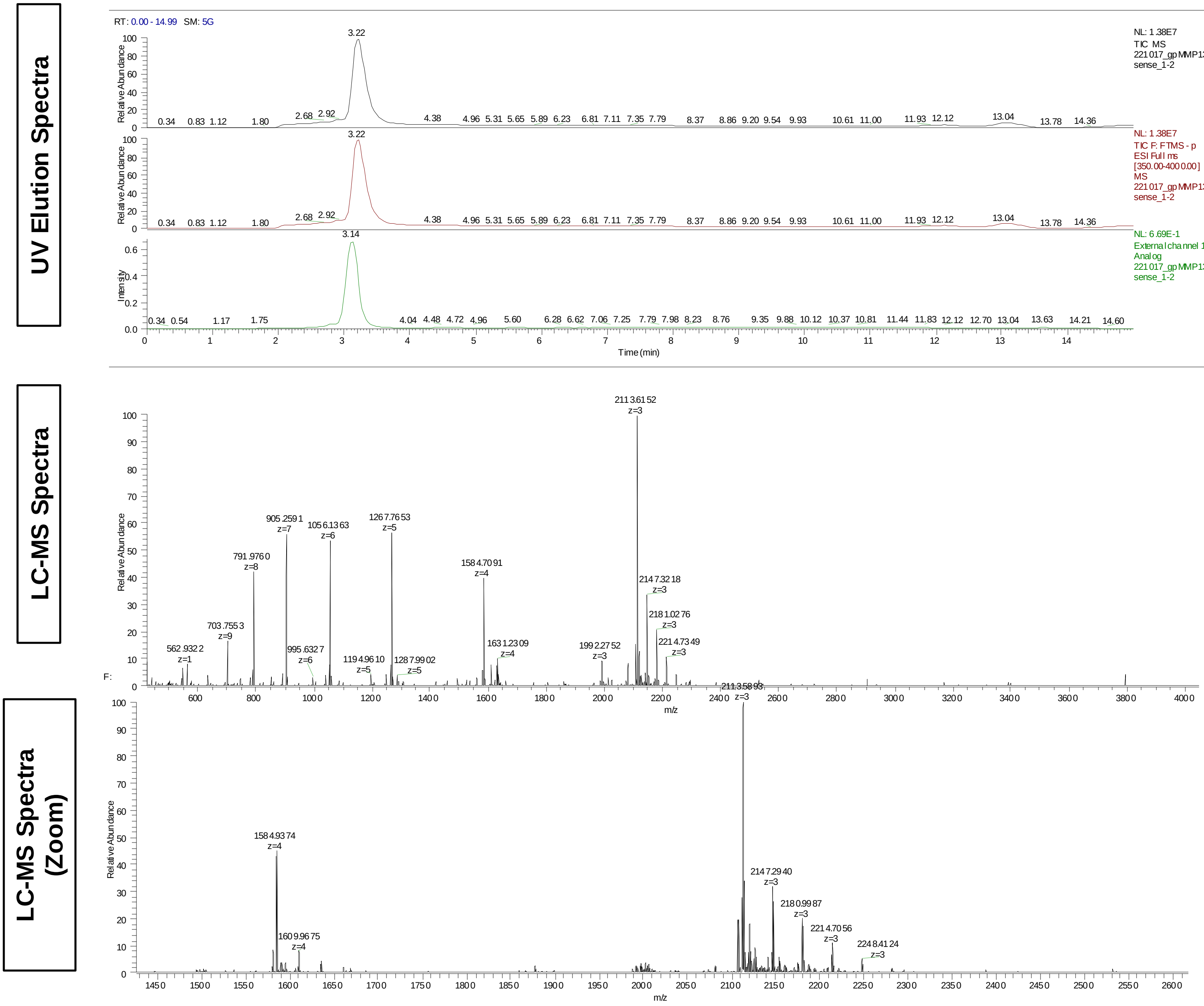

Supplementary Figure 34. LC-MS characterization of guinea pig MMP13 sense siRNA sequence.

UV Elution Spectra

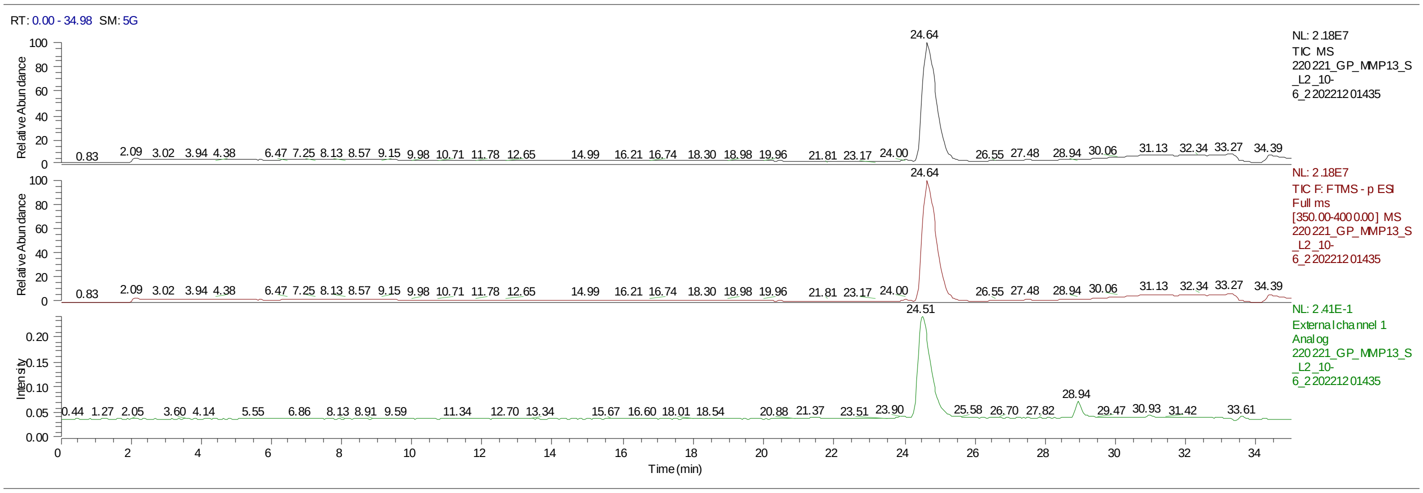

LC-MS Spectra

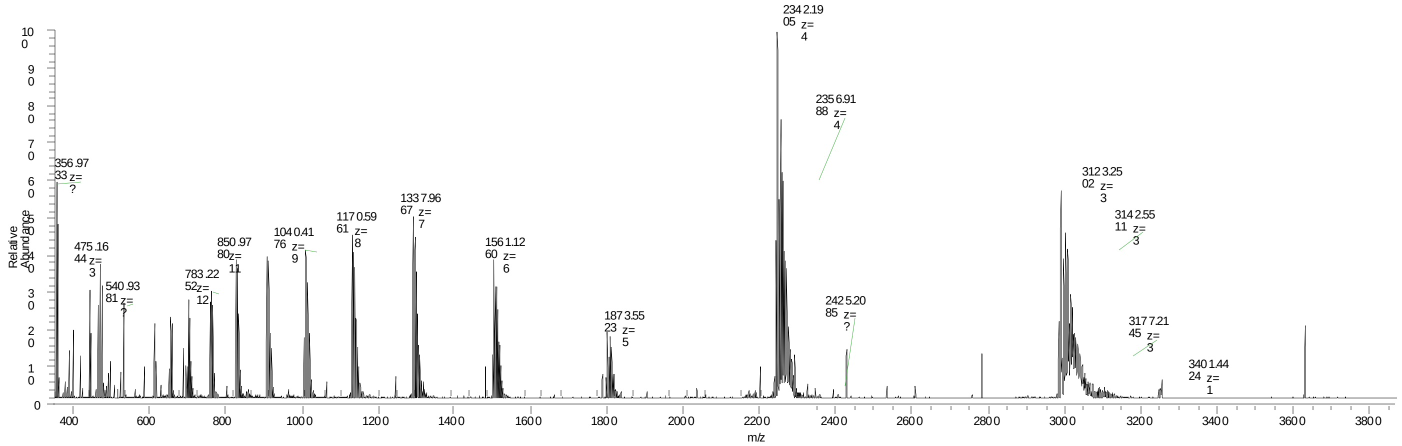

LC-MS Spectra (Zoom)

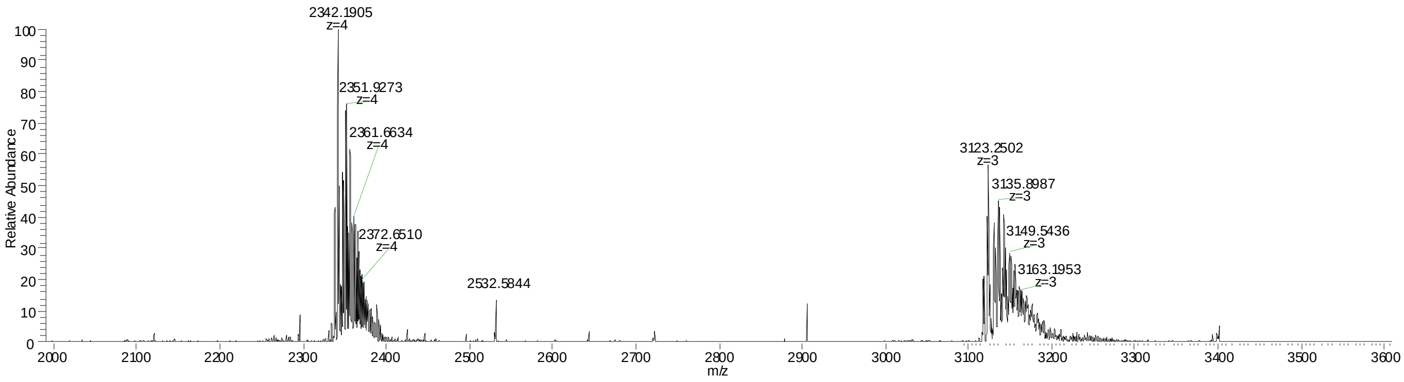

Supplementary Figure 35. LC-MS characterization of guinea pig MMP13 sense – <$(EG_{18}L)_2$ modifier siRNA sequence.

UV Elution Spectra

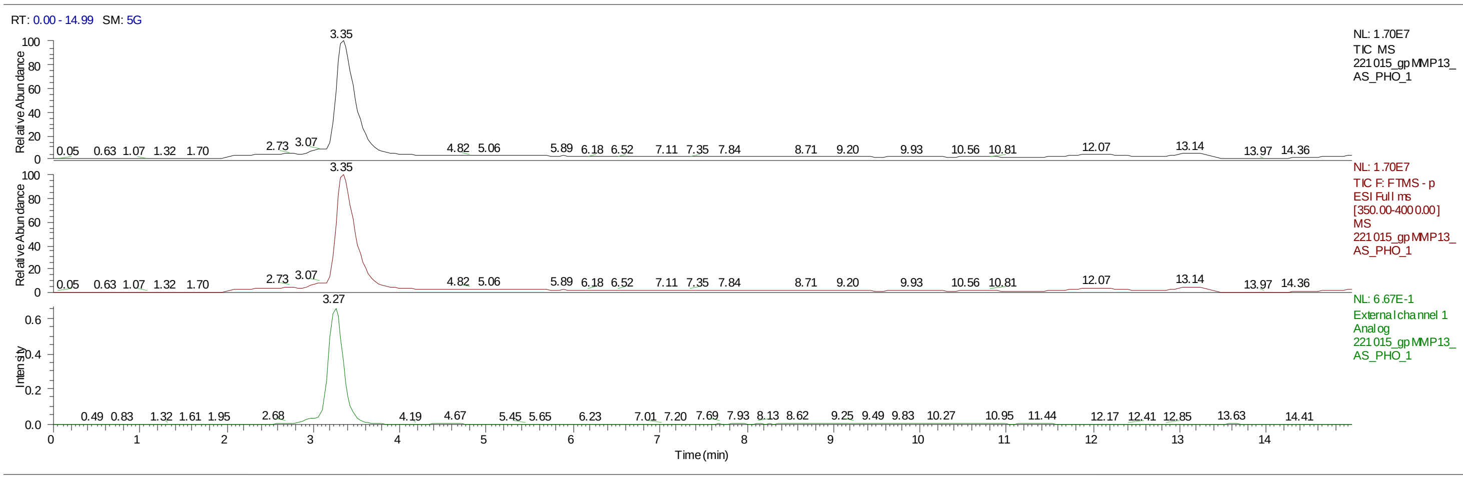

LC-MS Spectra

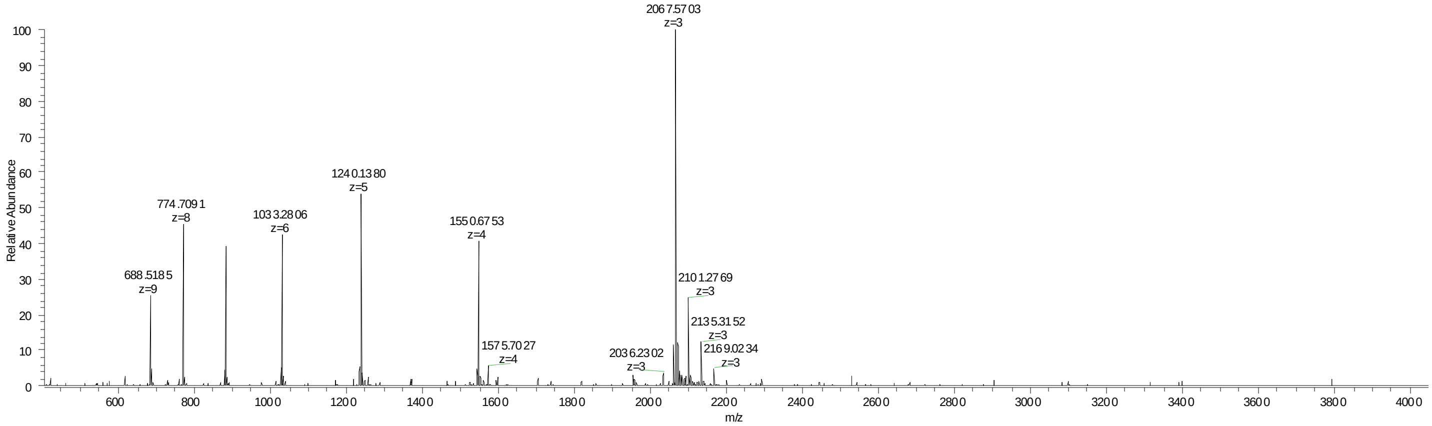

LC-MS Spectra (Zoom)

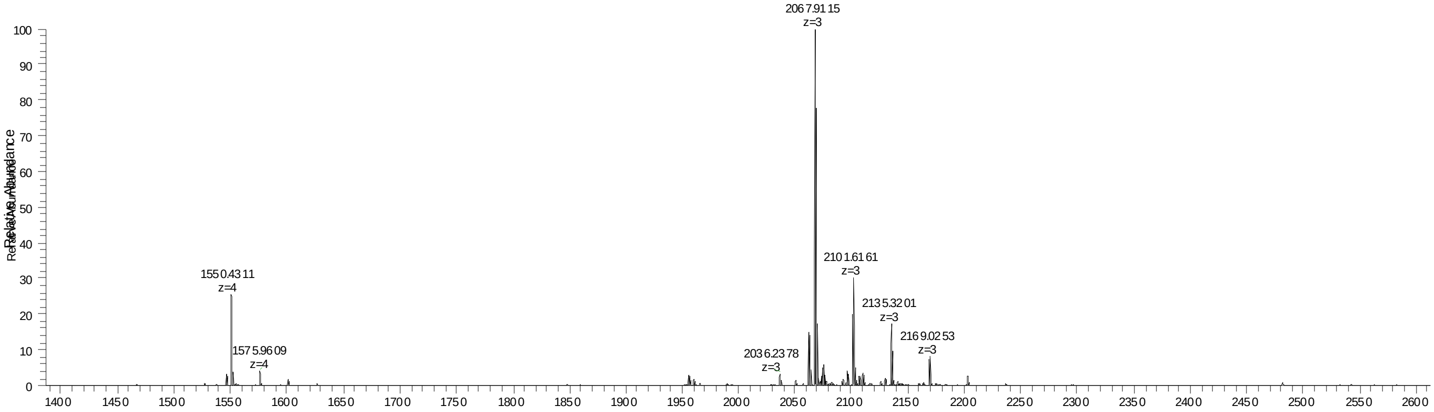

Supplementary Figure 36. LC-MS Characterization of Guinea Pig MMP13 Antisense – siRNA Sequence.
