# Supplementary material for: siRNA conjugate with high albumin affinity and degradation resistance for delivery and treatment of arthritis in mice and guinea pigs
Source: Nat Biomed Eng. 2025 May 16;9(8):1366–83. doi: 10.1038/s41551-025-01376-x (PMC12354308; doi:10.1038/s41551-025-01376-x)
Supplement: Supplementary file 2 — Reporting Summary [file 41551_2025_1376_MOESM2_ESM.pdf]

nature portfolio

Corresponding author(s): Craig L. Duvall

Last updated by author(s): 6/3/2024

Reporting Summary

Nature Portfolio wishes to improve the reproducibility of the work that we publish. This form provides structure for consistency and transparency in reporting. For further information on Nature Portfolio policies, see our Editorial Policies and the Editorial Policy Checklist.

Statistics

For all statistical analyses, confirm that the following items are present in the figure legend, table legend, main text, or Methods section.

n/a	Confirmed	
☐	☒	The exact sample size (*n*) for each experimental group/condition, given as a discrete number and unit of measurement
☐	☒	A statement on whether measurements were taken from distinct samples or whether the same sample was measured repeatedly
☐	☒	The statistical test(s) used AND whether they are one- or two-sided<br>*Only common tests should be described solely by name; describe more complex techniques in the Methods section.*
☒	☐	A description of all covariates tested
☐	☒	A description of any assumptions or corrections, such as tests of normality and adjustment for multiple comparisons
☐	☒	A full description of the statistical parameters including central tendency (e.g. means) or other basic estimates (e.g. regression coefficient) AND variation (e.g. standard deviation) or associated estimates of uncertainty (e.g. confidence intervals)
☐	☒	For null hypothesis testing, the test statistic (e.g. *F*, *t*, *r*) with confidence intervals, effect sizes, degrees of freedom and *P* value noted<br>*Give P values as exact values whenever suitable.*
☒	☐	For Bayesian analysis, information on the choice of priors and Markov chain Monte Carlo settings
☒	☐	For hierarchical and complex designs, identification of the appropriate level for tests and full reporting of outcomes
☒	☐	Estimates of effect sizes (e.g. Cohen's *d*, Pearson's *r*), indicating how they were calculated

Our web collection on statistics for biologists contains articles on many of the points above.

Software and code

Policy information about availability of computer code

Data collection	Living Image IVIS Software V4.4, SCANCO Medical microCT software suite V6.0, and FACSDiva software (BD Biosciences).
Data analysis	Graphpad Prism V9, Nikon NIS-Elements AR V4.30.01, Microsoft Excel Microsoft 365, SCANCO Medical microCT software suite V6.0, nSolver V3.0, and FlowJo v10 (TreeStar/BD Biosciences).

For manuscripts utilizing custom algorithms or software that are central to the research but not yet described in published literature, software must be made available to editors and reviewers. We strongly encourage code deposition in a community repository (e.g. GitHub). See the Nature Portfolio guidelines for submitting code & software for further information.

Data

Policy information about availability of data

All manuscripts must include a data availability statement. This statement should provide the following information, where applicable:

- Accession codes, unique identifiers, or web links for publicly available datasets
- A description of any restrictions on data availability
- For clinical datasets or third party data, please ensure that the statement adheres to our policy

The main data supporting the results in this study are available within the paper and its Supplementary Information. Raw and normalized nanoString datasets are available at the Gene Expression Omnibus under accession identifier XXX. The remaining raw and analysed datasets from the study are too large to be publicly shared, but they are available for research purposes from the corresponding author on reasonable request.

Research involving human participants, their data, or biological material

Policy information about studies with human participants or human data. See also policy information about sex, gender (identity/presentation), and sexual orientation and race, ethnicity and racism.

Reporting on sex and gender	*Use the terms sex (biological attribute) and gender (shaped by social and cultural circumstances) carefully in order to avoid confusing both terms. Indicate if findings apply to only one sex or gender; describe whether sex and gender were considered in study design; whether sex and/or gender was determined based on self-reporting or assigned and methods used. Provide in the source data disaggregated sex and gender data, where this information has been collected, and if consent has been obtained for sharing of individual-level data; provide overall numbers in this Reporting Summary. Please state if this information has not been collected. Report sex- and gender-based analyses where performed, justify reasons for lack of sex- and gender-based analysis.*
Reporting on race, ethnicity, or other socially relevant groupings	*Please specify the socially constructed or socially relevant categorization variable(s) used in your manuscript and explain why they were used. Please note that such variables should not be used as proxies for other socially constructed/relevant variables (for example, race or ethnicity should not be used as a proxy for socioeconomic status). Provide clear definitions of the relevant terms used, how they were provided (by the participants/respondents, the researchers, or third parties), and the method(s) used to classify people into the different categories (e.g. self-report, census or administrative data, social media data, etc.) Please provide details about how you controlled for confounding variables in your analyses.*
Population characteristics	*Describe the covariate-relevant population characteristics of the human research participants (e.g. age, genotypic information, past and current diagnosis and treatment categories). If you filled out the behavioural & social sciences study design questions and have nothing to add here, write "See above."*
Recruitment	*Describe how participants were recruited. Outline any potential self-selection bias or other biases that may be present and how these are likely to impact results.*
Ethics oversight	*Identify the organization(s) that approved the study protocol.*

Note that full information on the approval of the study protocol must also be provided in the manuscript.

Field-specific reporting

Please select the one below that is the best fit for your research. If you are not sure, read the appropriate sections before making your selection.

☒ Life sciences ☐ Behavioural & social sciences ☐ Ecological, evolutionary & environmental sciences

For a reference copy of the document with all sections, see nature.com/documents/nr-reporting-summary-flat.pdf

Life sciences study design

All studies must disclose on these points even when the disclosure is negative.

Sample size	In vitro studies were performed with at least 3 technical replicates. In the process of piloting the mechanical-overload osteoarthritis model, the differences between mice subjected to the model and healthy mice were used to educate a power analysis for sample-size selection. Not all tailvein and/or intra-articular injections were successfully administered, resulting in some reported variation in sample size between the in vivo treatment groups.
Data exclusions	No data were excluded from the analyses.
Replication	All in vitro experiments were performed with at least three technical replicates on more than one occasion to ensure reproducibility across experiments, and all in vivo experiments were performed with sufficient technical replicates to ensure reproducibility. All in vivo studies were done in one cohort, with the indicated sample sizes. All internal attempts at replication were successful.
Randomization	For the in vivo studies, the animals were randomly assigned to treatment groups at the outset of the study. Mice were assigned consecutive numbers across cages, and the groups were clustered consecutive numbers at random. All animals were age-matched, with no observed differences or selection criteria for a specific treatment group.
Blinding	The researcher who performed the mechanical joint loading and tail vein and/or intra-articular injections was not involved in the preparation of the injected treatments and was not involved in the design of the study groups. The histopathologist who completed the scoring of OA and RA severity by the DJD and OARSI metrics was blinded to the treatment given to the animals and was also not involved in the overall design of the study.

Reporting for specific materials, systems and methods

We require information from authors about some types of materials, experimental systems and methods used in many studies. Here, indicate whether each material, system or method listed is relevant to your study. If you are not sure if a list item applies to your research, read the appropriate section before selecting a response.

Materials & experimental systems

n/a	Involved in the study
☐	☒ Antibodies
☐	☒ Eukaryotic cell lines
☒	☐ Palaeontology and archaeology
☐	☒ Animals and other organisms
☒	☐ Clinical data
☒	☐ Dual use research of concern
☒	☐ Plants

Methods

n/a	Involved in the study
☒	☐ ChIP-seq
☐	☒ Flow cytometry
☒	☐ MRI-based neuroimaging

Antibodies

Antibodies used

Anti-mouse MMP13 antibody (ab39012, Abcam)
Anti-guinea pig MMP13 (1:100, ARP56350_P050, Aviva Systems Biology)
Goat anti-rabbit Alexa Fluor® 488 (1:500, ab150077, Abcam)
Lycopersicon Esculentum (Tomato) Lectin (LEL, TL) DyLight™ 488 (1:100, DL-1174-1)
C1,2C (Col 2 3/4Cshort) Polyclonal Rabbit Antibody (1:500, IBEX Pharmaceuticals 50-1035)
Anti-human albumin antibody (ab19180, Abcam)
Mouse Seroblock FcR (Bio-Rad)
CD45-PerCp/Cy5.5 (Biolegend)
FAP-AF488 (R&D Systems)
CD31-PE (Invitrogen)
CD3-SBV515 (Bio-Rad)
CD11b-APC/Cy7 (Biolegend)
F4/80-PE/Cy7 (Biolegend)
CD11c-BV605 (Biolegend)

Validation

For immunohistochemical staining for MMP13, slides were incubated with anti-MMP13 antibody for 1 hour at a 1:750 dilution. The antibody was validated by the commercial supplier for human species, and has been validated in multiple species in published studies, including mice (citation: Tsubosaka M et al. Gelatin hydrogels with eicosapentaenoic acid can prevent osteoarthritis progression in vivo in a mouse model. J Orthop Res N/A:N/A (2020)). The supplier confirms the reactivity of certain forms of MMP13 in the following statement: "ab39012 recognizes the latent proenzyme, at 60 Kd, as well as the active form at 48 Kd, and intermediate activation forms. It does not cross react with the other MMP family members. ab39012 recognizes the Hinge region of MMP13". It was validated on mouse small intestine.

Anti-guinea pig MMP13 (1:100, ARP56350_P050, Aviva Systems Biology) was validated on guinea pig small intestine.

C1,2C (Col 2 3/4Cshort) Polyclonal Rabbit Antibody (1:500, IBEX Pharmaceuticals 50-1035) was validated in mouse and human tracheal cartilage.

Anti-human albumin antibody (ab19180, Abcam) was validated with human plasma.

Mouse Seroblock FcR (Bio-Rad), CD45-PerCp/Cy5.5 (Biolegend), FAP-AF488 (R&D Systems) , CD31-PE (Invitrogen), CD3-SBV515 (Bio-Rad), CD11b-APC/Cy7 (Biolegend), F4/80-PE/Cy7 (Biolegend), CD11c-BV605 (Biolegend) were validated on synovial tissue harvest from healthy and osteoarthritis mouse knee joints.

Eukaryotic cell lines

Policy information about cell lines and Sex and Gender in Research

Cell line source(s)

ATDC5 (ECACC 99072806) cells (Millipore Sigma Inc., Burlington, MA). Cells were validated and maintained according to the instructions of the supplier: Culture Medium: DMEM: Ham's F12 (1:1) + 2mM Glutamine + 5% Foetal Bovine Serum (FBS). Subculture routine: Split sub-confluent cultures (70-80%) i.e. seeding at 1-3 x 10,000 cells/cm² using 0.25% trypsin or trypsin/EDTA; CO_2; 37°C subculture every 2–3 days.
Primary Guinea Pig Knee Chondrocytes: Harvested from guinea pig knee joints. Culture Medium: DMEM + 2mM Glutamine +10% Foetal Bovine Serum (FBS).

Authentication

Cells were authenticated according to the standards of the European Collection of Authenticated Cell Cultures, and cultured within the advised passage limits.

Mycoplasma contamination

All cells tested negative for mycoplasma, and were expanded in media containing a mycoplasma prophylactic (plasmocin).

Commonly misidentified lines
(See ICLAC register)

No commonly misidentified cell lines were used.

Animals and other research organisms

Policy information about studies involving animals; ARRIVE guidelines recommended for reporting animal research, and Sex and Gender in Research

Laboratory animals	C57Bl/6 mice were sourced from Jackson Laboratories. Dunkin Hartley guinea pigs were sourced from Charles River Laboratories.
Wild animals	The study did not involve wild animals.
Reporting on sex	Only males were used.
Field-collected samples	The study did not involve samples collected from the field.
Ethics oversight	All animal studies were performed in accordance with protocols reviewed and approved by the Vanderbilt University IACUC Office.

Note that full information on the approval of the study protocol must also be provided in the manuscript.

Flow Cytometry

Plots

Confirm that:

☒ The axis labels state the marker and fluorochrome used (e.g. CD4-FITC).

☒ The axis scales are clearly visible. Include numbers along axes only for bottom left plot of group (a 'group' is an analysis of identical markers).

☒ All plots are contour plots with outliers or pseudocolor plots.

☒ A numerical value for number of cells or percentage (with statistics) is provided.

Methodology

Sample preparation	Two synovia were digested together in a volume of 1.5 mL digestion media (DMEM with 400 µg/mL collagenase IV, liberase, and DNaseI). Synovia were digested for 40 minutes at 37°C with intermittent vortexing at 0, 15, 30, 35 and 40 minutes, and then pelleted at 500 x g for 5 minutes at 4°C. Each treatment group (vehicle and Cy5-siRNA<(EG18L)2) was comprised of 5 male C57/B6 mice, which were pooled and divided into the controls and samples. Cold FACS buffer (PBS containing 1% FBS, 2 mM EDTA) was used for all antibody staining and wash steps. Non-specific binding was blocked using Mouse Seroblock FcR (Bio-Rad) for 5 minutes.
Instrument	BD LSRFortessa cytometer
Software	FACSDiva software (BD Biosciences), then data compensation and analysis was performed using FlowJo v10 (TreeStar/BD Biosciences).
Cell population abundance	We identified synovial fibroblasts, endothelial cells, macrophages, monocytes, dendritic cells, and T-cells. There was a high total percentage of Cy5 positive cells in the synovium (83.1% of all synovial cells). Fibroblasts, macrophages, and endothelial cells exhibited the highest proportion of Cy5-positive cells.
Gating strategy	All cell populations were gated off fluorescence-minus-one (FMO) controls. The Cy5 gate was established using the vehicle mouse sample and applied to each cell population and a %Cy5 positive proportion and median fluorescence intensity (MFI) was calculated for all synovial cells and each cell type separately.

☒ Tick this box to confirm that a figure exemplifying the gating strategy is provided in the Supplementary Information.
